# Reverse thiophosphorylase activity of a glycoside phosphorylase in the synthesis of an unnatural Manβ1,4GlcNAc library†

**DOI:** 10.1039/d3sc04169g

**Published:** 2023-09-29

**Authors:** Tessa Keenan, Natasha E. Hatton, Jack Porter, Jean-Baptiste Vendeville, David E. Wheatley, Mattia Ghirardello, Alice. J. C. Wahart, Sanaz Ahmadipour, Julia Walton, M. Carmen Galan, Bruno Linclau, Gavin J. Miller, Martin A. Fascione

**Affiliations:** a Department of Chemistry, University of York Heslington York YO10 5DD UK martin.fascione@york.ac.uk; b School of Chemical and Physical Sciences and Centre for Glycosciences, Keele University Keele, Staffordshire ST5 5BG UK g.j.miller@keele.ac.uk; c School of Chemistry, University of Southampton Highfield Southampton SO17 1BJ UK; d School of Chemistry, University of Bristol Cantock's Close Bristol BS8 1TS UK; e Department of Organic and Macromolecular Chemistry, Ghent University Campus Sterre, Krijgslaan 281-S4 Ghent 9000 Belgium

## Abstract

β-Mannosides are ubiquitous in nature, with diverse roles in many biological processes. Notably, Manβ1,4GlcNAc a constituent of the core *N*-glycan in eukaryotes was recently identified as an immune activator, highlighting its potential for use in immunotherapy. Despite their biological significance, the synthesis of β-mannosidic linkages remains one of the major challenges in glycoscience. Here we present a chemoenzymatic strategy that affords a series of novel unnatural Manβ1,4GlcNAc analogues using the β-1,4-d-mannosyl-*N*-acetyl-d-glucosamine phosphorylase, BT1033. We show that the presence of fluorine in the GlcNAc acceptor facilitates the formation of longer β-mannan-like glycans. We also pioneer a “reverse thiophosphorylase” enzymatic activity, favouring the synthesis of longer glycans by catalysing the formation of a phosphorolysis-stable thioglycoside linkage, an approach that may be generally applicable to other phosphorylases.

## Abstract 2

A carbohydrate phosphorylase is utilised in the synthesis of unnatural Manβ1,4-GlcNAc and longer β-mannan like glycans, including formation of phosphorolysis-stable thioglycoside linkages *via* novel "reverse thiophosphorylase" enzymatic activity.

## Graphical abstract

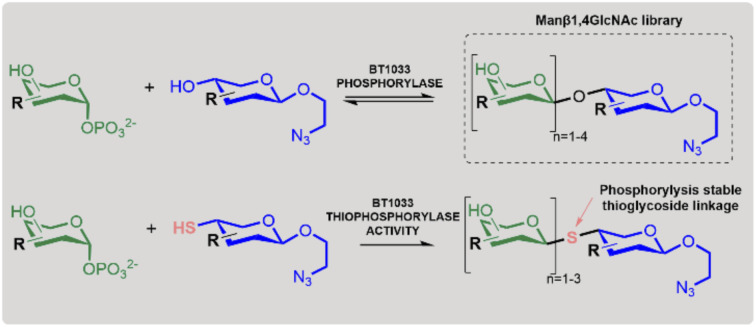

Glycoside phosphorylases (GPs) naturally catalyse the breakdown of glycosidic bonds between glycans (phosphorolysis).^1^ However, these useful biocatalysts can also be harnessed in a synthetic “reverse phosphorolysis” direction (Fig. 1A) requiring only simple sugar-1-phosphate donors for the synthesis of diverse glycosides.^2,3^ Yet the inherent reversibility of GPs can limit their utility in the synthetic direction. Herein we explore the use of unnatural substrates to favour “reverse phosphorolysis” using a GP active on β-mannosides and in the process pioneer “reverse thiophosphorylase” enzymatic activity, wherein formation of a phosphorolysis-stable thioglycoside linkage (Fig. 1B) facilitates the synthesis of longer glycans.

**Fig. 1:**
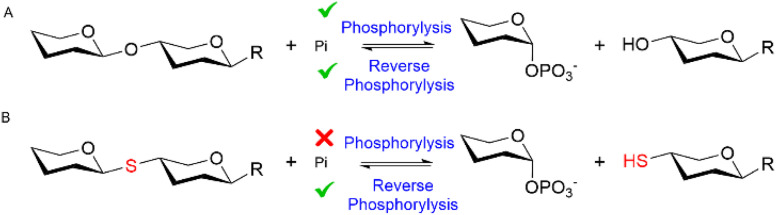
The reversible GP catalyzed reaction (A). Proposed irreversible “reverse thiophosphorylase” activity with a 4SH-thiol acceptor (B).

β-Mannosides are highly prevalent in nature,^4^ with diverse roles in biological processes including energy storage^5^ and cell wall biosynthesis.^6^ Notably the ubiquitous ManGlcNAc_2_ motif within eukaryotic *N*-glycans^7^ contains a Manβ1,4GlcNAc disaccharide, which was recently identified as a novel immune modulator in autoimmune disease.^8,9^ Manβ1,4GlcNAc has shown potential as a new activator of STING (stimulator of interferon genes pathway) triggering a broad immune response in macrophages.^8^ STING is a component of the innate immune system and a key mediator of inflammation.^10^ Therefore small molecule activators are emerging as a promising strategy in cancer immunotherapy.^11^ Despite its striking biological significance and recent advances in the chemical synthesis of such linkages,^12^ the efficient assembly of β-mannosides still remains one of the major challenges in glycoscience.

Herein we utilize a GP-mediated chemoenzymatic approach^13^ for the synthesis of β-mannosides in the form of an unnatural library of Manβ1,4GlcNAc-based glycans, including a number of extended glycans. We incorporate unnatural functionality into the enzymatic building blocks through chemical synthesis and show that when a 4-SH nucleophile or 6F group are present in the GlcNAc acceptor, this facilitates the extension of Manβ1,4GlcNAc producing longer β-mannan like glycans. This approach not only affords access to a series of novel, unnatural Manβ1,4-GlcNAcs which may have potential in immunotherapy, but also represents a benchmark for the utility of GPs for thioglycoside synthesis. With more than 190 GPs that have been characterized to date, if this “reverse thiophosphorylase” activity was observed more broadly in other GPs, it could provide straightforward access to a wide range of thioglycosides.^14^

For the synthesis of our Manβ1,4GlcNAc analogues we investigated the inverting β-1,4-d-mannosyl-*N*-acetyl-d-glucosamine phosphorylase from *Bacteroides thetaiotaomicron* (BT1033).^13^ BT1033 is a GH130 family phosphorylase, previously shown to catalyse the transfer of Man from α-d-mannose-1-phosphate (Man1-P) onto *N*-acetyl-d-glucosamine (GlcNAc) to produce Manβ1,4GlcNAc by reverse phosphorolysis. To investigate the substrate promiscuity of BT1033, we screened a series of chemically synthesised Man1-P donors (3–10) and GlcNAc acceptors (11–14) (Fig. 2). The GlcNAc acceptors were designed with an azido-propyl handle to provide an accessible point for bioconjugation and this was exploited in our glycan detection methodology (Fig. 2A). Imidazolium-based ionic liquid tags (ITags) are highly sensitive mass spectrometry (MS) probes that enable low detection limits, due to their dominant ionizability by MS.^16^ To facilitate the semi-quantitative detection of the Manβ1,4GlcNAc products in our reactions, as well as any unreacted acceptor, the reaction products were labelled with an alkyne-functionalised ITag 1 using a copper-catalysed alkyne–azide cycloaddition (CuAAC) reaction and analysed by liquid-chromatography coupled to mass spectrometry (LC-MS). The relative conversion of starting material to product was determined by comparing the ionisation intensities of the unreacted azido-propyl linked GlcNAc (GlcNAc-N_3_) acceptor to the azido-propyl linked Manβ1,4GlcNAc products (Fig. 1B and ESI Section 5†). First, we assessed the suitability of GlcNAc-N_3_11 as an acceptor mimic for BT1033, with Man1-P 2 as a donor. In preliminary studies (data not shown) we observed some enzyme-mediated hydrolysis of Man1-P 2. Therefore, in reactions containing Man1-P 2, the donor was supplied in excess (5–10 eq.) relative to the acceptor. Additionally, the donor was supplied in significant excess (5 × 10^4^–1 × 10^5^ eq.) relative to the enzyme to drive the reaction in favour of the synthetic “reverse phosphorolysis” reaction. LC-MS analysis showed an ion consistent with the mass of the Manβ1,4GlcNAc-ITag disaccharide (*m*/*z* 764) as expected (Fig. S11†). Additionally, we observed an ion consistent with the mass of the Manβ1,4GlcNAc-ITag disaccharide + 162 Da (*m*/*z* 926). BT1033 was previously shown to have weak synthetic activity with d-mannose as an acceptor when using Man-1P as a donor^13^ whilst able to use chitobiose as an acceptor, demonstrating that it is capable of producing longer-glycans. Therefore, we proposed that the product at *m*/*z* 926 was a Man_2_β1,4GlcNAc-ITag trisaccharide. Overall, we observed 74% conversion to disaccharide 15 and 4% to trisaccharide 16 (Fig. 2B). Next, we screened BT1033 for activity towards eight unnatural Man-1P analogues (3–10) with acceptor 11 (Fig. 2B and S3–S10†). C6-Chloro Man-1P 4 was best tolerated by BT1033, with 61% conversion to disaccharide observed after 24 h (Fig. S3†). Moderate conversions of C5-methyl Man-1P 3 and C6-methyl Man-1P 5 to disaccharide were also observed at 51% and 44%, respectively (Fig. S4 and S5†). Conversion of C6-fluoro Man-1P 6 and C6-azido Man-1P 7 to disaccharide were lower at 16% and 11% respectively (Fig. S6 and S8†), suggesting that these were poor substrates for the enzyme. No conversion of C6-*gem*-difluoro Man-1P 8 was observed, which was not surprising considering the poor turnover of 6. Additionally, no turnover of C6-hydroxamic acid Man-1P 9 or C6-amine Man-1P 10 were observed (Fig. S7, S9 and S10†). There was no evidence of longer glycan chain formation when using any of the unnatural Man-1Ps. In summary, the results of the unnatural Man-1P substrate screen suggest that BT1033 has little or no activity towards C6-modified analogues with groups larger than the native CH_2_OH. Whilst poor turnover of C6-azido 7 and C6-amine 10 Man-1Ps was observed, the chlorine in disaccharide 18 could allow for further derivatization at the C6-position to an azide or amine.

**Fig. 2:**
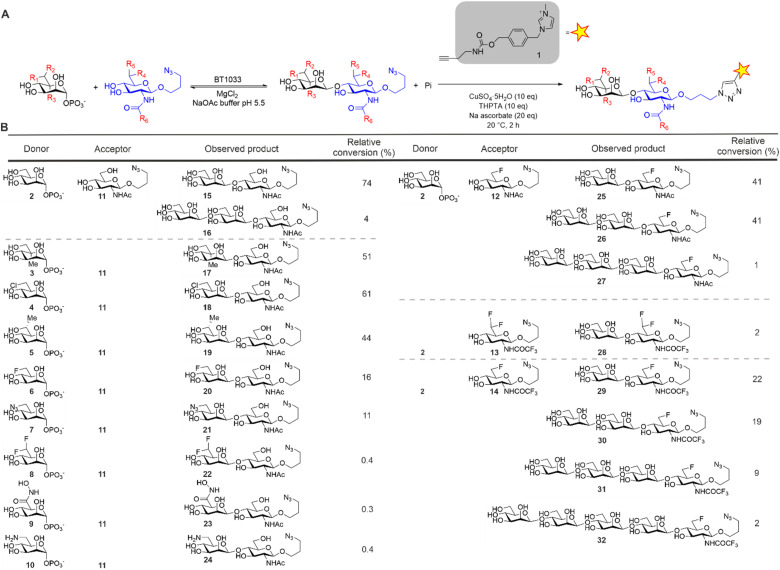
(A) ITag screening methodology for BT1033 reactions. Reaction mechanism depicted in reverse phosphorolysis direction. (B) BT1033 activity towards unnatural donors and acceptors.^3,15^

Next, we screened for activity towards fluorinated GlcNAc-N_3_ acceptors 12–14, with Man-1P 2 (Fig. 2B). Fluorination, whilst having little effect on the overall conformation of a glycan,^17^ is known to affect stereo-electronic properties and can therefore modulate biological function.^18^ 6F-GlcNAc-N_3_12 and 6F-GlcNTFA-N_3_14 were tolerated by the enzyme, producing 83% and 52% total conversion to product respectively. For both acceptors, not only were the anticipated disaccharide products observed at conversions of 41% (12) and 22% (14) respectively, but also masses consistent with the production of longer Mannan-type glycans. For example, with 12 we observed products consistent with disaccharide (*m*/*z* 766, Manβ1,4-6F-GlcNAc-ITag, 41%), trisaccharide (*m*/*z* 928, Man_2_β1,4-6F-GlcNAc-ITag, 41%) and tetrasaccharide (*m*/*z* 1090, Man_3_β1,4-6F-GlcNAc-ITag, 1%) formation (Fig. S12†). With 14, in addition to the expected disaccharide (*m*/*z* 820, Manβ1,4-6F-GlcNTFA-ITag, 22%) we observed trisaccharide (*m*/*z* 982, Man_2_β1,4-6F-GlcNTFA-ITag, 19%), tetrasaccharide (*m*/*z* 1144, Man_3_β1,4-6F-GlcNTFA-ITag, 9%) and pentasaccharide (*m*/*z* 1306, Man_4_β1,4-6F-GlcNTFA-ITag, 2%, Fig. S14†). In contrast, only low levels of conversion of 6,6-diF-GlcNTFA 13 to disaccharide (2%) was observed (Fig. S13†). Taken together, this data indicates BT1033 can tolerate acceptors with fluorination at C6 position and within the NAc substituent. Increasing the number of fluorines at C6 in the acceptor resulted in poorer turnover by BT1033, with such presence in carbohydrate substrates previously shown to reduce the catalytic efficiency of some enzymes.^19^ However, the presence of a single fluorine in the acceptor interestingly appeared to facilitate the formation of longer glycans by BT1033, when compared to GlcNAc-N_3_11. We hypothesized that fluorination in the acceptors may reduce the rate of the competing phosphorolysis reaction, altering the reaction equilibrium and resulting in an accumulation of the reverse phosphorolysis disaccharide product, which could subsequently serve as an acceptor for further mannosylation using 2. To investigate this further, we tested BT1033 for activity with a 4-SH-GlcNAc-N_3_ analogue 33 and compared this to its activity towards 11 under the same conditions (Fig. 3). We anticipated that the reaction would yield a Manβ1,4-*S*-GlcNAc-N_3_34 thioglycoside (Fig. 3A). Thioglycosides are carbohydrate mimetics that are often resistant to hydrolysis and have elicited significant interest in recent years as probes for structural and biological studies, and as enzyme inhibitors.^20,21^ We hypothesized that if BT1033 was able to use a thiol as an acceptor with 2 (in the synthetic direction) the reaction may become irreversible due to the stability of the resultant thioglycoside to phosphorolysis. Following LC-MS analysis of reactions with 33 under disulfide reducing conditions, we observed masses consistent with the expected disaccharide (*m*/*z* 780, Manβ1,4-*S*-GlcNAc-ITag), as well as trisaccharide (*m*/*z* 942, Man_2_β1,4-*S*-GlcNAc-ITag) and tetrasaccharide (*m*/*z* 1104, Man_3_β1,4-*S*-GlcNAc-ITag) formation (Fig. 3B). Overall, there was a greater proportion of reverse phosphorolysis product at the end of the reaction using 33, compared with 11 (Fig. 3C).

**Fig. 3:**
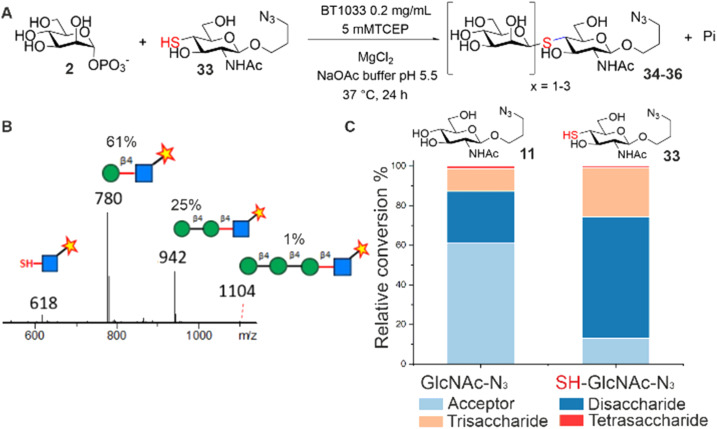
(A) BT1033 turnover of thio-GlcNAc-N_3_33 to produce the Manβ1,4-*S*-GlcNAc-N_3_34 thioglycoside. Reaction mechanism depicted in the reverse phosphorolysis direction. (B) LC-MS analysis showing di-, tri- and tetrasaccharide thioglycoside formation. (C) Comparative product distribution in BT1033 reactions with 11 and 33.

Using 11, we observed mostly acceptor (61%), some disaccharide (26%) and trisaccharide (11%), and low-level tetrasaccharide (1%). Comparatively, for 33 the majority of the product observed was disaccharide (61%), with some trisaccharide (25%) and tetrasaccharide (1%). These findings are consistent with the accumulation of phosphorolysis resistant Manβ1,4-*S*-GlcNAc-N_3_34. Although the specific activity of BT1033 towards GlcNAc-N_3_11 (3.20 μmol min^−1^ mg^−1^) was ∼2-fold higher than towards SH-GlcNAc-N_3_33 (1.59 μmol min^−1^ mg^−1^), enzyme titration curves with the respective acceptors highlight the beneficial effect that the thiol has on the final conversion to product in the competing phosphorolysis reaction, with near full conversion of SH-GlcNAc-N_3_33 achieved in 30 min, with only a 3-fold excess of Man-1P donor relative to acceptor and 0.33 mg mL^−1^ of enzyme (Fig. S35A†). In contrast, under the same conditions only ∼20% conversion of GlcNAc-N_3_11 was observed, while ∼ 40% was observed with 0.04 mg mL^−1^ of enzyme (Fig. S35B†).

To further showcase the utility of BT1033 for chemoenzymatic β-mannosylation we assembled a library of unnatural azidopropyl-linked Manβ1,4-GlcNAc glycans on a semi-preparative scale, including thioglycoside di, tri and tetrasaccharides (34–36) and fluorinated di, tri, tetra and pentasaccharides (25–27, 37), in isolated yields ranging from 5% to 68%, (Table 1, ESI Section 8†). The structures of synthesised glycans were validated by 1D and 2D NMR and HRMS (ESI Sections 8 and 9†), with β-glycosidic linkages confirmed using IPAP HSQC, which measures each anomeric carbons ^1^*J*_CH_ coupling constant (Tables S3 and S4†).^22^ Although similar trends were observed the isolated product yields differed from the relative conversions measured by MS, which is likely a reflection of the change in scale and challenges associated with purification of longer oligosaccharides. To validate BT1033 was able to operate irreversibly as a “reverse thiophosphorylase” we investigated the stability of our purified glycan library to BT1033 catalysed phosphorolysis (Fig. 4). As expected Manβ1,4-GlcNAc 15 underwent rapid phosphorolysis, with ∼50% breakdown to acceptor 11 observed after 2 min and ∼65% after 24 h. Intriguingly, although Manβ1,4-6F-GlcNAc 25 showed a greater proportion of phosphorolysis over 24 h compared to 15 (78% *vs.* 70% breakdown to acceptor respectively), a lower amount of phosphorolysis was observed at 2 min (15% *vs.* 50% breakdown to acceptor respectively). This slower rate of phosphorolysis may therefore account for the observed formation of C6-fluorinated tri-, tetra-, and pentasaccharide by reverse phosphorolysis. The presence of the 6F-GlcNAc moiety appeared to have minimal effect on breakdown of fluorinated trisaccharide 26 to disaccharide 25, when compared to trisaccharide 16, which contains the natural GlcNAc moiety. Notably, 6Cl-Man β1,4-GlcNAc 18 also showed a much lower proportion (∼20%) of phosphorolysis-mediated product after 24 h compared to 15 (∼65%). Again, potentially accounting for the accumulation of 18 in the reverse phosphorolysis reaction when using 4. As hypothesised Manβ1,4-*S*-GlcNAc 34 proved resistant to phosphorolysis, with no cleavage of the thioglycoside observed after 24 h indicating that the replacement of the alcohol nucleophile with a thiol in the acceptor does enable the phosphorolase to operate irreversibly in the synthetic direction. However, the presence of the Manβ1,4-*S*-GlcNAc thioglycoside linkage appears to have no effect on the extent of phosphorolysis of trisaccharide 35 to thioglycoside 34, compared to the natural trisaccharide 16, similar to observations for 26. Tetrasaccharides containing the 6F functionality 27 and the thioglycoside linkage 36 respectively, were subjected to phosphorolysis and showed the expected breakdown to trisaccharide after 24 h. Whilst the phosphorolysis of 27 afforded a distribution of products (from acceptor to even longer glycans, indicating reverse phosphorolysis was occurring), the reaction with 36 halted as disaccharide accumulated due to the stability of the thioglycoside linkage. Finally, the 6F pentasaccharide 37, similarly to 27, afforded a distribution of products from acceptor to hexasaccharide, indicative of reverse phosphorolysis having occurred.

**Table 1:** Manβ1,4-GlcNAc-N_3_ analogues produced on scale

Donor	Acceptor	Product	Yield (%)	Amount (mg)
4	11	6Cl-Manβ1,4-GlcNAc 18	68	2.6
2	11	Manβ1,4-GlcNAc-N_3_15	56	6.5
		Manβ1,4-Manβ1,4-GlcNAc-N_3_16	15	2.4
2	12	Manβ1,4-6F-GlcNAc-N_3_25	12	1.4
		Manβ1,4-Manβ1,4-6F-GlcNAc-N_3_26	13	2.1
		Manβ1,4-Manβ1,4-Manβ1,4-6F-GlcNAc-N_3_27	7	1.4
		Manβ1,4-Manβ1,4-Manβ1,4-Manβ1,4-6F-GlcNAc-N_3_37	5	1.3
2	33	Manβ1,4-*S*-GlcNAc-N_3_34	20	3.8
		Manβ1,4-Manβ1,4-*S*-GlcNAc-N_3_35	23	5.9
		Manβ1,4-Manβ1,4-Manβ1,4-*S*-GlcNAc-N_3_36	11	3.4

**Fig. 4:**
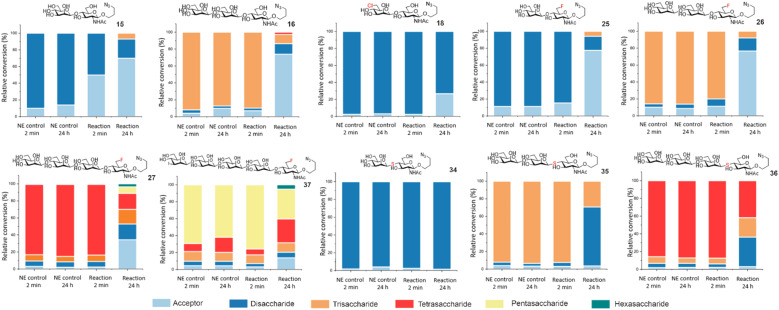
Phosphorolysis of Manβ1,4-GlcNAc-N_3_ analogues, with relative conversions determined by ITag LC-MS analysis. NE denotes: no enzyme control.

BT1033 belongs to the GH130 enzyme family, which includes β-mannoside phosphorylases MGP (4-*O*-β-d-mannosyl-d-glucose phosphorylase) and Uhgb_MP (β-1,4-mannosyl-*N*-glycan phosphorylase) from *Bacteroides* sp.^13,23,24^ Guided by structural studies of MGP and Uhgb_MP, GH130 catalysis is proposed to proceed through a “proton shuttle” mechanism (Fig. 5A). For the synthetic reaction, it is hypothesised that mannose in the −1 subsite, existing in an unstable B_2,5_ boat conformation, is deprotonated by a catalytic Asp residue (Asp131 in MGP or Asp104 in Uhgb_MP) at the 3-OH which subsequently deprotonates the incoming GlcNAc acceptor *via* its 3-OH group.^23,25^ Amino acid sequence alignment of BT1033 and Uhgb_MP, identified Asp101 as the putative catalytic residue in BT1033 (Fig. S36†). Superimposition of a BT1033 alphafold model with the structure of Uhgb_MP in complex with β-d-mannose and phosphate, showed that BT1033 Asp101 overlayed with Uhgb_MP Asp104, supporting this hypothesis (Fig. 5B). To reinforce this proposed role of Asp101 in BT1033 catalysis, we also produced a BT1033 D101A mutant and investigated the synthetic activity of the enzyme with both GlcNAc-N_3_11 and SH-GlcNAc-N_3_33, using the natural Man-1P 2 donor (Fig. S23†). As anticipated, BT1033 D101A displayed no activity towards GlcNAc-N_3_11, confirming that this residue is required for catalysis. Interestingly, no activity towards SH-GlcNAc-N_3_33 was observed either implying that despite the lower p*K*_a_ of the thiol acceptor, deprotonation of the incoming nucleophile within the active site is still required. Previously the enzymatic synthesis of diverse thioglycosides using “thioglycoligases”, glycosidase mutants with their catalytic acid/base residues mutated to an alanine or glycine, have been achieved and extensively explored by the Withers group^26^ and others.^20,27^ In contrast to thioglycoligases, we demonstrate here that the reverse thiophosphorylase activity of BT1033 is abolished in in the absence of the catalytic base, thus suggesting that a deeper understanding of the proposed GH130 ‘proton shuttle’ mechanism may be required to aid the design of more efficient reverse thiophosphorylases.

**Fig. 5:**
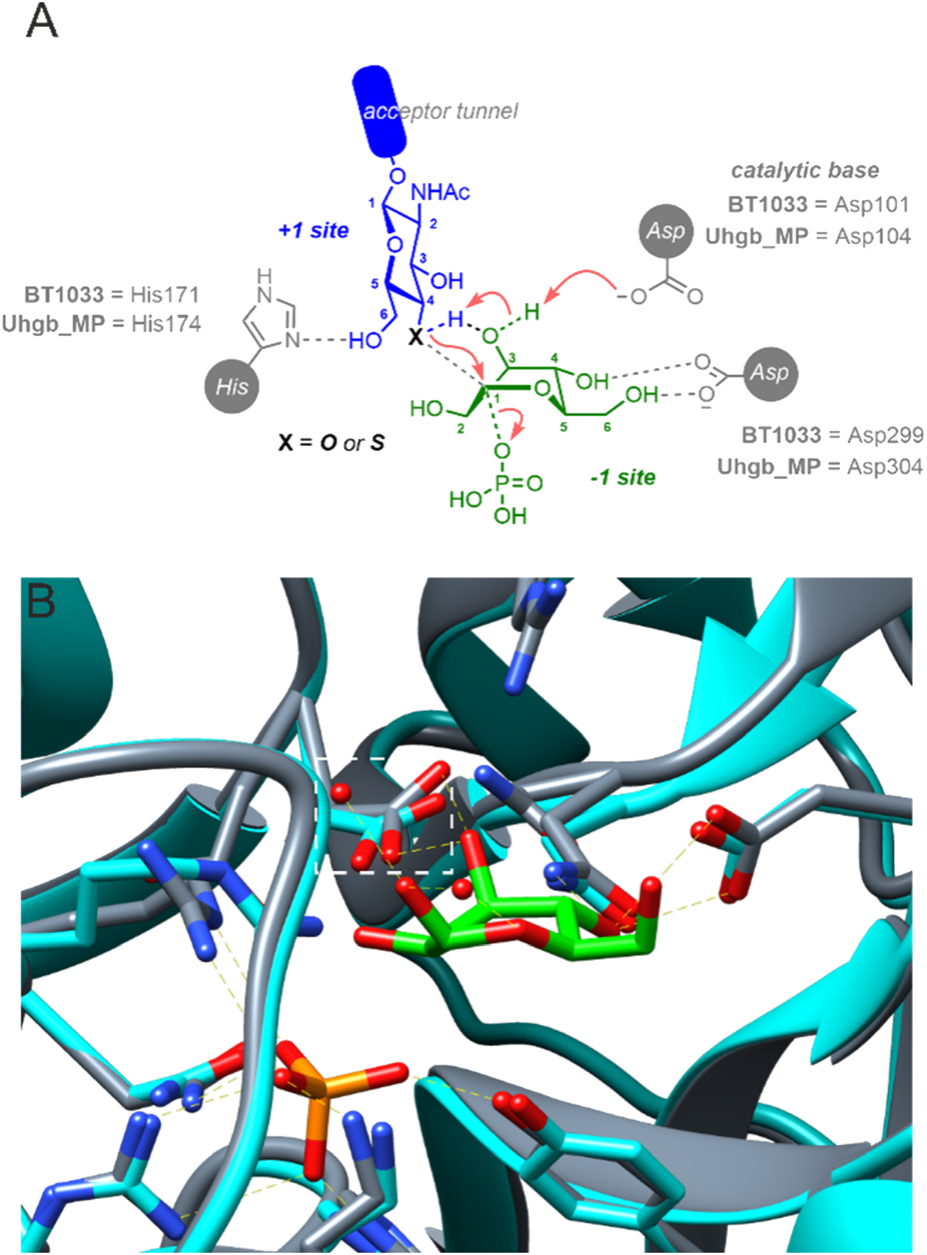
(A) Proposed mechanism of BT1033. (B) Crystal structure of Uhgb_MP (4UDJ, grey) superimposed with a BT1033 alphafold model (turquoise), showing that BT1033 Asp101 overlays with Uhgb_MP Asp104 (orange box). Uhgb_MP structure shown in complex with β-d-mannose and phosphate.

In summary, we have demonstrated that BT1033 can be exploited to access diverse Manβ1,4-GlcNAc analogues, and longer β-mannan like glycans. We also establish novel reverse thiophosphorylase activity favouring the synthesis of longer glycans by initially catalysing the formation of a stable thioglycoside linkage. Following incorporation of unnatural functionality into the enzymatic building blocks through chemical synthesis, we systematically screened BT1033 for activity towards these unnatural donors and acceptors in a MS-based strategy using a “clickable” ITag to facilitate product ionisation and detection. BT1033 displayed activity towards C6-modified donors, most notably 6Cl-Man-1P 4. Fluorinated acceptors were also turned over by the enzyme, and interestingly the presence of the fluorine appears to also facilitate extension of Manβ1,4-GlcNAc with Man to produce longer β-mannan like glycans, likely through slowing the rate of phosphorolysis. Whilst enzymatic strategies for the synthesis of thioglycosides to date have focused on the exploitation of “thioglycoligases”,^20,26,27^ to our knowledge the use of a wildtype GP to synthesise thioglycosides has not been explored. If this “reverse thiophosphorylase” activity was generally applicable to other GH130 phosphorylases, it could provide simple yet dynamic access to a diverse range of thioglycosides. As Manβ1,4GlcNAc has shown potential as a immune activator,^10^ the thioglycoside products of the reverse thiophosphorylase activity of BT1033 could have potential as non-hydrolysable β-mannose containing activators for immunotherapy. Furthermore, extension of the reverse thiophosphorylase approach to thiol substituted sugar-1P donors, could have potential utility in the construction of thiooligosaccharide homopolymers.

## Data availability

The datasets supporting this article have been uploaded as part of the ESI.†

## Author contributions

TK and NH screened donors and acceptors and performed chemoenzymatic synthesis. TK performed phosphorolysis experiments. TK, NH and JW performed protein production. JP, AW, SA, DW JBV and MG performed the chemical synthesis. MAF, GJM, CG and BL supervised the project. TK, NH, GM and MAF wrote the manuscript and designed the study. All authors analysed the data and commented on the manuscript.

## Conflicts of interest

The authors declare no competing financial interest.

## Supplementary Material

SC-014-D3SC04169G-s001
