# Supplementary material for: Reverse thiophosphorylase activity of a glycoside phosphorylase in the synthesis of an unnatural Manβ1,4GlcNAc library
Source: Chem Sci. 2023 Sep 29;14(42):11638–46. doi: 10.1039/d3sc04169g (PMC10619541; doi:10.1039/d3sc04169g)
Supplement: SC-014-D3SC04169G-s001 [file SC-014-D3SC04169G-s001.pdf]

Electronic Supplementary Material (ESI) for Chemical Science.
This journal is © The Royal Society of Chemistry 2023

Supporting Information

Reverse thiophosphorylase activity of a glycoside phosphorylase in the synthesis of an unnatural Manβ1,4GlcNAc library

‡Tessa Keenan[1], ‡Natasha Hatton[1], Jack Porter[2], Jean-Baptiste Vendeville[3], David Wheatley[3], Mattia Ghirardello[4], Alice. J. C. Wahart[2], Sanaz Ahmadipour[2], Julia Walton[1], Carmen Galan[4], Bruno †Linclau[3], Gavin J. Miller[2*] and Martin A. Fascione[1*].

[1]Department of Chemistry, University of York, Heslington, York, YO10 5DD, UK. [2]School of Chemical and Physical Sciences and Centre for Glycosciences, Keele University, Keele, Staffordshire ST5 5BG, UK. [3]School of Chemistry, University of Southampton, Highfield, Southampton, SO17 1BJ, U.K. [4]School of Chemistry, University of Bristol, Cantock's Close, Bristol BS8 1TS, UK. [5]Department of Organic and Macromolecular Chemistry, Ghent University, Campus Sterre, Krijgslaan 281-S4, Ghent, 9000, Belgium

Experimental:

1. General

All chemicals were purchased from Acros Organics, Alfa Aesar, Biosynth Carbosynth, Fisher Scientific, Fluorochem, Sigma Aldrich, VWR or TCI Chemicals and were used without further purification unless otherwise stated. NMR spectra were recorded on a Bruker Avance 400 spectrometer, JEOL 400 spectrometer or a Bruker AVIIIHD 500 spectrometer. The chemical shift data for each signal are given as δ in units of parts per million (ppm) relative to tetramethylsilane, where δ = 0.00 ppm. The number of protons (n) for a given resonance is indicated by nH. The multiplicity of each signal is indicated by s (singlet), br s (broad singlet), d (doublet), t (triplet), q (quartet), p (pentet), sep (septet), dd (doublet of doublets), ddd (doublet of doublet of doublets), dddd (doublet of doublet of doublet of doublets), dt (doublet of triplets), tt (triplet of triplets), dqd (doublet of quartets of doublets) or m (multiplet). Resonances were assigned using HH-COSY and CH-HSQC. All NMR chemical shifts (δ) were recorded in ppm and coupling constants (J) are reported in Hz. Assignment of ^{1}H and ^{13}C atoms in NMR follows standard pyranose ring numbering. Topspin 4.0.6 and MestReNovax64 were primarily used for processing the spectral data. pH was measured using a FisherbrandTM accumetTM AE150 Benchtop pH Meter. Anhydrous DMF, MeOH, pyridine and NEt_3 were obtained from Sure/Seal^{TM} bottles *via* chemical suppliers. Anhydrous THF, DCM and toluene were obtained by passing solvent through activated alumina columns and dispensed from a PureSolv MD ASNA solvent purification system and stored over 4 Å molecular sieves. Unless otherwise stated, all reactions were conducted using anhydrous solvents, under an atmosphere of N_2 which was passed through a Drierite® drying column. Analytical thin layer chromatography (TLC) was carried out on pre-coated 0.25 mm Merck KgaA 60 F254 silica gel plates. Visualisation was by adsorption of UV light, or thermal development after dipping in a MeOHic solution of sulfuric acid (5% v/v), p-Anisaldehyde stain or $KMnO_4$ solution. Automatic flash chromatography was carried out on silica gel (Reveleris® X2 system) under a positive pressure of compressed N_2. Manual flash column chromatography was performed using silica gel (230−400 mesh (40−63 µm)). Reaction mixture residues were dried under vacuum using rotary evaporation and high vacuum oil pump.

2. Mass spectrometry

Small-molecule high resolution mass spectrometry (HRMS) data were obtained at RT on a Bruker Daltonics microTOF mass spectrometer coupled to an Agilent 1200 series LC system, an Agilent 6530 Q-TOF, LQT Orbitrap XL1, Waters (Xevo, G2-XS TOF or G2-S ASAP) Micromass LCT or aVG Quattro mass spectrometers. Preparative RP-HPLC was performed on a Luna PREP C18 column (10 µm, 250 x 21.2 mm) using a linear gradient of acetonitrile/water (5:95 to 95:5, v:v, over 20 min at a flow rate of 14 mL/min).

High Performance Liquid Chromatography-Electrospray Ionisation Mass Spectrometry (LC-MS) was accomplished using a Dionex UltiMate® 3000 LC system (ThermoScientific) equipped with an UltiMate® 3000 Diode Array Detector (probing 250-400 nm) in line with a Bruker HCTultra ETD II system (Bruker Daltonics), using Chromeleon® 6.80 SR12 software (ThermoScientific), Compass 1.3 for esquire HCT Build 581.3, esquireControl version 6.2, Build 62.24 software (Bruker Daltonics), and Bruker compass HyStar 3.2-SR2, HyStar version 3.2, Build 44 software (Bruker Daltonics) at The University York Centre of Excellence in Mass Spectrometry (CoEMS). All mass spectrometry was conducted in positive ion mode. Data analysis was performed using ESI Compass 1.3 DataAnalysis, Version 4.1 software (Bruker Daltonics). For HILIC-LC-MS, analyses were carried out using a SeQuant ZIC®-HILIC HPLC column. Water, 0.1% formic acid by volume (solvent A), and acetonitrile, 0.1 % formic acid (solvent B) were used as the mobile phase at a flow rate of 0.3 ml/min at room temperature. A multi-step gradient of 9 min was programmed as follows: 95 % B for 0.5 min, followed by a linear gradient to 5 % B over 4.5 min, followed by 5 % B for an additional 0.5 min. A linear gradient to 95 % B was used to re-equilibrate the column. For analyses carried out by reverse-phased LC-MS , samples were analysed using a Symmetry C18 5 µm 3.0 x 150 mm reverse-phase column. Water + 0.1% formic acid by volume (solvent A) and acetonitrile + 0.1% formic acid (solvent B) were used as a mobile phase at a flow rate of 300 µL min^{-1} at room temperature. A multi-step gradient of 7.5 min was programmed as follows: 95% A for 1.0 min, followed by a linear gradient to 95% B over 6.5 min, followed by 95% B for an additional 1.0 min. A linear gradient to 95% A was used to equilibrate the column.

3. Chemical synthesis

All derivatised mannose-1-phophates (Man-1P) used in this work were synthesised and reported previously by *Beswick et al.* [1] (C6-fluoro Man-1P **6**, C6-gem-difluoro Man-1P **8**, C6-azido Man-1P **7** and C5-methyl Man-1P **3**), *Ahmadipour et al.* [2] (C6-chloro Man-1P **4**, C6-amine Man-1P **10**), *Beswick et al.* [3] (C6-hydroxamic acid Man-1P **9**) and *Ahmadipour et al.* [4] (C6-methyl Man-1P **5**). GlcNAc-N_3 **11**, 6F-GlcNAc-N_3 **12**, 6,6-diFGlcNTFA-N_3 **13** and 6F-GlcNTFA-N_3 **14** were synthesised and reported previously, by *Richards, Keenan et al.* [5]

3.1 Synthesis of Bis[3-azidopropyl (2-acetamido-2,4-dideoxy)-β-D-glucopyranos-4-yl] disulphide 33

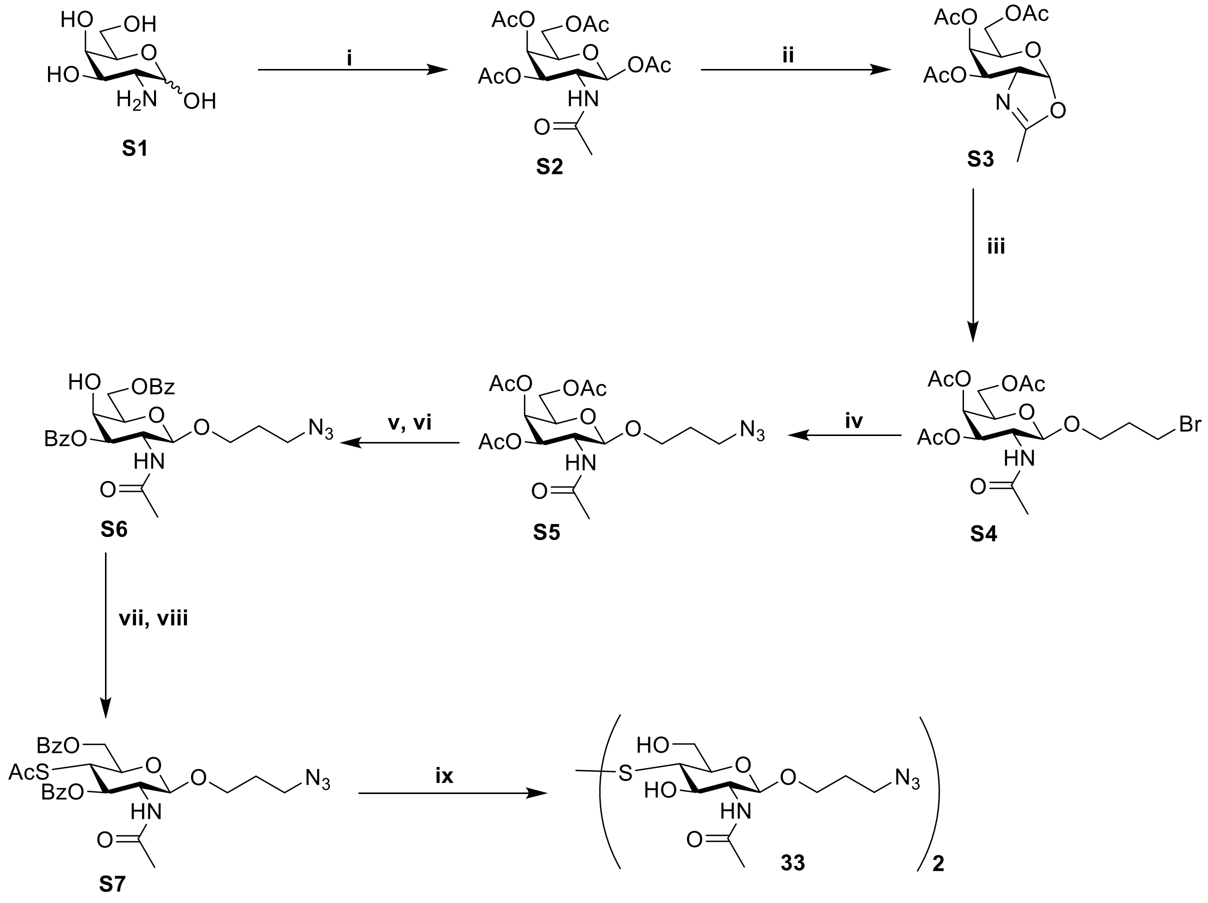

Scheme S1. Synthesis of Bis[3-azidopropyl (2-acetamido-2,4-dideoxy)-β-D-glucopyranos-4-yl] disulphide 33 from galactosamine hydrochloride. Reagents and conditions i) Galactosamine hydrochloride, pyridine, Ac_2O, 0 °C to RT, overnight; ii) DCE, TMSOTf, 50 °C, overnight; iii) 3-bromopropan-1-ol, CSA, 42 °C, 1 h; iv) DMF, NaN_3, 50 °C, 2 h; v) MeOH, 1M NaOH, RT, 2 h; vi) Pyridine, DCM, Benzoyl chloride, -60 °C; vii) DCM, pyridine, Tf_2O, 0 °C, 1 h; viii) Pyridine, KSAc, RT; ix) MeOH, RT, 30 minutes, NaOMe, RT.

3.1.1 2-acetamido-1,3,4,6-tetra-*O*-acetyl-2-deoxy-β-D-galactopyranoside S2

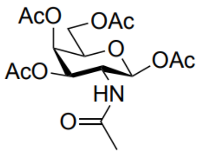

Galactosamine hydrochloride **S1** (5.00 g, 23.2 mmol, 1.0 equiv.) was dissolved in pyridine (50 mL) and acetic anhydride (10 mL) was added to the solution at 0 °C. The reaction mixture was allowed to warm to RT and stirred overnight. TLC analysis revealed complete consumption of the starting material (100 % EtOAc). The reaction mixture was co-evaporated with toluene (3 x 50 mL) and the crude solid recrystallised from MeOH (100 mL) yielding **S2** (7.10 g, 18.2 mmol, 79%) as a white solid. ^{1}H NMR (400 MHz, $CDCl_3$) *δ* 5.70 (d, *J* = 8.8 Hz, 1H, H-1), 5.41 (d, *J* = 9.5 Hz, 1H, N*H*), 5.38 (dd, *J* = 3.3, 0.8 Hz, 1H, H-4), 5.09 (dd, *J* = 11.3, 3.3 Hz, 1H, H-3), 4.50 – 4.40 (m, 1H, H-2), 4.21 – 4.08 (m, 2H, H-6a, H-6b), 4.02 (td, *J* = 6.5, 1.1 Hz, 1H, H-5), 2.17 (s, 3H, OAc), 2.13 (s, 3H, OAc), 2.05 (s, 3H, OAc), 2.02 (s, 3H, OAc), 1.94 (s, 3H, OAc); ^{13}C NMR (101 MHz, $CDCl_3$) *δ* 170.8 (C=O, Ac), 170.4 (C=O, Ac), 170.3 (C=O, Ac), 170.2 (C=O, Ac), 169.6 (C=O, Ac), 93.1 (C1), 71.9 (C5), 70.3 (C3), 66.3 (C4), 61.3 (C6), 49.8 (C2), 23.3 (Ac-CH_3), 20.9 (Ac-CH_3), 20.67 (Ac-CH_3), 20.65 (Ac-CH_3); HRMS *m/z* (ES+) [Found (M+H)+ 390.1395, $C_{16}H_{24}O_{10}N_1$ requires M+ 390.1395]. Data matched those previously reported.[6]

3.1.2 2-Methyl-4,5-dihydro-(3,4,6-tri-O-acetyl-1,2-dideoxy-α-D-galactopyranoso)[2,1-d]-1,3-oxazole S3

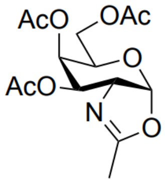

A solution of peracetate **S2** (1.28 g, 3.30 mmol, 1.0 equiv.) in dichloroethane (40 mL) was treated slowly with TMSOTf (0.90 mL, 4.95 mmol, 1.5 equiv.). After stirring overnight at 50 °C, TLC analysis revealed reaction completion (to R_f = 0.44, EtOAc). The reaction mixture was quenched with saturated aqueous $NaHCO_3$ (100 mL). The layers were separated, and the aqueous phase was extracted with DCM (2 × 100 mL). The combined organic layers were dried ($MgSO_4$) and concentrated under reduced pressure. Purification of the crude product by column chromatography (Hexane/EtOAc, 0-100%) delivered **S3** (623 mg, 1.89 mmol, 57%) as a colourless oil. R_f = 0.44 (EtOAc); ^{1}H NMR (400 MHz, $CDCl_3$) δ 6.00 (d, *J* = 6.8 Hz, 1H, H-1), 5.46 (t, *J* = 2.9 Hz, 1H, H-4), 4.91 (dd, *J* = 3.3, 7.4 Hz, 1H, H-3), 4.29 – 4.09 (m, 3H, H-6a, H-6b, H-5), 4.00 (m, 1H, H-2), 2.13 (s, 3H, Ac), 2.07 (s, 6H, Ac), 2.05 (s, 3H, Ac); ^{13}C NMR (101 MHz, $CDCl_3$) δ 170.5 (C=O, Ac), 170.1 (C=O, Ac), 169.8 (C=O, Ac), 166.4 (C=N), 101.5 (C1), 71.8 (C3), 69.5 (C5), 65.3 (C4), 63.6 (C2), 61.6 (C6), 20.8 (Ac-CH_3), 20.7 (Ac-CH_3), 20.6 (Ac-CH_3), 14.4 (CH_3); HRMS *m/z* (ES+) [Found (M+H)+ 330.1182, $C_{14}H_{20}O_8N_1$ requires M+ 320.1183]. Data matched those previously reported.[7]

3.1.3 3-Bromopropyl (2-acetamido-3,4,6-tri-O-acetyl-2-deoxy)-β-D-galactopyranoside S4

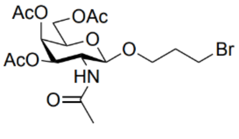

A solution of oxazole **S3** (238 mg, 0.723 mmol, 1.0 equiv.) in 3- bromopropan-1-ol (3.3 mL, 36.2 mmol, 50.0 equiv.) was treated with camphor sulfonic acid (CSA) (84.0 mg, 0.361 mmol, 0.5 equiv.) and left to stir for 1 h at 42 °C. TLC analysis revealed conversion of the starting material to a higher spot (to R_f = 0.50, EtOAc). After

allowing the reaction mixture to reach RT, the mixture was washed with saturated aqueous $NaHCO_3$ (50 mL), and water (50 mL). The combined organic phases were dried ($MgSO_4$), filtered and concentrated under reduced pressure. The crude residue was purified by column chromatography (hexane/EtOAc, 0-100%) to deliver **S4** (124 mg, 0.265 mmol, 37%) as a colourless oil. R_f = 0.50 (EtOAc); $[\alpha]_D^{28}$ = +0.48 (*c* = 1.0, $CHCl_3$); ^{1}H NMR (400 MHz, $CDCl_3$) *δ* 5.59 (d, *J* = 8.9 Hz, 1H, N*H*), 5.36 (dd, *J* = 3.3, 0.7 Hz, 1H, H-4), 5.20 (dd, *J* = 11.3, 3.4 Hz, 1H, H-3), 4.61 (d, *J* = 8.4 Hz, 1H, H-1), 4.22 – 4.07 (m, 3H, H-6a, H-6b, H-2), 4.06 – 4.00 (m, 1H, OC*H*H), 3.93 (td, *J* = 6.6, 1.0 Hz, 1H, H-5), 3.68 (td, *J* = 9.3, 4.2 Hz, 1H, OC*H*H), 3.58 – 3.47 (m, 2H, CH_2), 2.24 – 2.16 (m, 2H, CH_2), 2.15 (s, 3H, Ac), 2.06 (s, 3H, Ac), 2.01 (s, 3H, Oc), 1.99 (s, 3H, Ac); ^{13}C NMR (101 MHz, $CDCl_3$) *δ* 170.7 (C=O, Ac), 170.48 (C=O, Ac), 170.47 (C=O, Ac), 170.3 (C=O, Ac), 101.9 (C1), 70.7 (C5), 70.1 (C3), 67.3 (OCH_2), 66.7 (C4), 61.5 (C6), 51.3 (C2), 32.1 (CH_2), 30.6 (CH_2), 23.5 (Ac-CH_3), 20.71 (Ac-CH_3); HRMS *m/z* (ES+) [Found (M+H)+ 468.0866, $C_{17}H_{27}O_9N_1Br$ requires M+ 468.0864].

3.1.4 3-Azidopropyl (2-acetamido-3,4,6-tri-O-acetyl-2-deoxy)-β-D-galactopyranoside S5

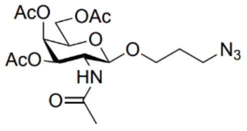

A solution of bromide **S4** (124 mg, 0.265 mmol, 1.0 equiv.) in DMF (10 mL) was treated with NaN*3* (34.0 mg, 0.530 mmol, 2.0 equiv.) at RT and then warmed to 50 °C. After 2 hours, TLC revealed reaction completion (to R_f = 0.45, EtOAc). The reaction was then cooled to RT, filtered through Celite®, and concentrated under reduced pressure. The crude residue was purified by flash chromatography (hexane/EtOAc, 0-100%) to deliver **S5** (93.0 mg, 0.216 mmol, 82%) as a white foam. R_f = 0.45 (EtOAc); $[\alpha]_D^{29}$ = -4.5 (c = 1.0, $CHCl_3$); ^{1}H NMR (400 MHz, $CDCl_3$) *δ* 5.57 (d, *J* = 8.8 Hz, 1H, N*H*), 5.36 (d, *J* = 2.6, 1H, H-4), 5.22 (dd, *J* = 11.3, 3.4 Hz, 1H, H-3), 4.64 (d, *J* = 8.4 Hz, 1H, H-1), 4.20 – 4.10 (m, 2H, H-6a, H-6b), 4.09 – 4.00 (m, 1H, H-2), 4.01 – 3.96 (m, 1H, OC*H*H), 3.93 (td, *J* = 6.6, 0.9 Hz, 1H, H-5), 3.65 – 3.56 (m, 1H, OC*H*H), 3.44 – 3.34 (m, 2H, CH_2), 2.15 (s, 3H, Ac), 2.05 (s, 3H, Ac), 2.01 (s, 3H, Ac), 1.97 (s, 3H, Ac), 1.95 – 1.82 (m, 2H, CH_2); ^{13}C NMR (101 MHz, $CDCl_3$) *δ* 170.6 (C=O, Ac), 170.5 (C=O, Ac), 170.4 (C=O, Ac), 170.3 (C=O, Ac), 101.4 (C1), 70.7 (C5), 70.0 (C3), 66.7 (C4), 66.3 (OCH_2), 61.5 (C6), 51.4 (C2), 48.1 (CH_2), 28.9 (CH_2), 23.5 (Ac-CH_3), 20.71 (Ac-CH_3), 20.69 (Ac-CH_3); HRMS *m/z* (ES+) [Found (M+H)+ 431.1775, $C_{17}H_{27}O_9N_4$ requires M+ 431.1773].

3.1.5 3-Azidopropyl (2-acetamido-3,6-di-O-benzoyl-2-deoxy)-β-D-galactopyranoside S6

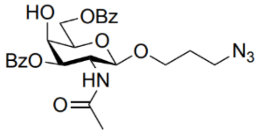

To a solution of peracetate **S5** (93.0 mg, 0.216 mmol, 1.0 equiv.) in MeOH (5 mL) was added 1M NaOH (5 mL). The reaction mixture stirred at RT for 2 h until consumption of the starting material by TLC (to R_f = 0.35, DCM/MeOH, 85/15). The reaction mixture was neutralized by addition of IR-120(H+) ion exchange resin, filtered and washed with MeOH (100 mL). The filtrate was concentrated under reduced pressure and the crude residue progressed without further purification. A solution of crude 3-azidopropyl (2-

acetamido-2-deoxy)-β-D-galactopyranoside (66.0 mg, 0.22 mmol, 1.0 equiv.) in pyridine (2.5 mL) and DCM (2.5 mL) was cooled to -60 °C and treated dropwise with benzoyl chloride (55 µL, 0.475 mmol, 2.2 equiv.). The reaction mixture was maintained at -60 °C until reaction completion by TLC (to R_f = 0.30, 1/1, hexane/EtOAc). Saturated aqueous $NaHCO_3$ (10 mL) was added with stirring and the aqueous layer was extracted with DCM (3 × 20 mL). The combined organic layers were washed with water (10 mL), dried ($MgSO_4$) and evaporated under reduced pressure, yielding a crude product, which was purified by flash chromatography (EtOAc/hexane, 0- 100%) to deliver **S6** (90.0 mg, 0.175 mmol, 81%) as an off-white foam. R_f = 0.30 (1:1 hexane/EtOAc); $[\alpha]_D^{22}$ = +4.9 (c = 1.0, $CHCl_3$); ^{1}H NMR (400 MHz, $CDCl_3$) δ 8.08 – 7.97 (m, 4H, ArH), 7.61 – 7.46 (m, 2H, ArH), 7.43 – 7.34 (m, 4H, ArH), 6.04 (d, J = 8.9 Hz, 1H, N*H*), 5.37 (dd, J = 11.2 Hz, 3.1 Hz, 1H, H-3), 4.71 (d, J = 8.3 Hz, 1H, H-1), 4.67 – 4.54 (m, 2H, H-6a, H-6b), 4.44 (dt, J = 11.1, 8.8 Hz, 1H, H-2), 4.25 (d, J = 2.8 Hz, 1H, H-4), 4.09 – 4.00 (m, 1H, H-5), 3.97 (dt, J = 10.7, 5.5 Hz, 1H, OC*H*H), 3.63 (ddd, J = 9.8 Hz, 8.3 Hz, 4.8 Hz, 1H, OC*H*H), 3.45 – 3.32 (m, 2H, CH_2), 1.89 (s, 3H, Ac), 1.80 – 0.94 (m, 2H, CH_2); ^{13}C NMR (101 MHz, CDCl3) δ 170.6 (C=O, Ac), 166.5 (C=O, Bz), 166.4 (C=O, Bz), 133.6 (Ar-C), 133.3 (Ar-C), 129.9 (Ar-CH), 129.7 (Ar-CH), 129.7 (Ar-CH), 129.1 (Ar-CH), 128.54 (Ar-CH), 128.45 (Ar-CH), 101.4 (C1), 73.6 (C3), 72.3 (C5), 66.9 (C4), 66.1 (OCH_2), 63.0 (C6), 50.8 (C2), 48.1 (CH_2), 29.0 (CH_2), 23.3 (Ac-CH_3); HRMS *m/z* (ES+) [Found (M+H)+ 513.1977, $C_{25}H_{29}O_8N_4$ requires M+ 513.1980].

3.1.6 3-Azidopropyl (2-acetamido-4-S-acetyl-3,6-di-O-benzoyl-2-deoxy)-β-D-glucopyranoside S7

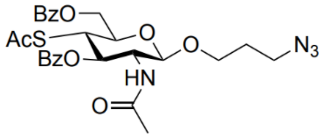

A solution of alcohol **S6** (141 mg, 0.280 mmol, 1.0 equiv.) in DCM (4 mL) and pyridine (1 mL) was treated at 0 °C with Tf_2O (106 µL, 0.640 mmol, 2.3 equiv.) in three portions. After 1 h of stirring at this temperature, DCM (50 mL) was added and the solution washed sequentially with ice cold 1M HCl (50 mL), cold saturated aqueous $NaHCO_3$ (50 mL) and cold brine (50 mL). The combined organic phases were dried ($MgSO_4$) and concentrated under reduced pressure. A solution of the crude residue in pyridine (5 mL) was treated with KSAc (114 mg, 0.530 mmol, 3.0 equiv.). The suspension was stirred at RT until complete consumption of the starting material by TLC (to R_f = 0.67, hexane/EtOAc, 7/3). The reaction was diluted with DCM (50 mL), washed with water (3 × 50 mL), brine (50 mL), dried ($MgSO_4$) and concentrated under reduced pressure. The crude material was purified by column chromatography (EtOAc/hexane, 0-30%) delivering **S7** (74.0 mg, 0.123 mmol, 47%) as a white foam. R_f = 0.67 (hexane/EtOAc, 7/3); $[\alpha]_D^{25}$ = +21.8 (c = 1.0, $CHCl_3$); ^{1}H NMR (400 MHz, $CDCl_3$) δ 8.12 – 8.08 (m, 2H, ArH), 8.00 – 7.96 (m, 2H, ArH), 7.63 – 7.54 (m, 2H, ArH), 7.51, 7.40 (m, 4H, ArH), 5.71 (d, J = 8.8 Hz, 1H, NH), 5.49 (t, J = 10.3 Hz, 1H, H-3), 4.72 – 4.66 (m, 2H, H-1, H-6a), 4.53 (dd, J = 12.1, 4.8 Hz, 1H, H-6b), 4.13 (dt, J = 10.2, 8.8 Hz, 1H, H-2), 4.08 – 3.93 (m, 3H, H-4, H-5, OC*H*H), 3.62 (ddd, J = 9.7, 8.2, 4.8 Hz,

1H, OC*H*H), 3.42 – 3.30 (m, 2H, CH_2), 2.19 (s, 3H, SAc), 1.87 (s, 3H, NAc), 1.94 – 1.75 (m, 2H, CH_2); ^{13}C NMR (101 MHz, $CDCl_3$) *δ* 192.6 (C=O, SAc) 170.2 (C=O, Ac), 166.7 (C=O, Bz), 166.3 (C=O, Bz), 133.6 (Ar-C), 133.2 (Ar-C), 130.0 (Ar-CH), 129.8 (Ar-CH), 128.9 (Ar-CH), 128.6 (Ar-CH), 128.5 (Ar-CH), 101.2 (C1), 72.8 (C5), 72.3 (C3), 66.0 (OCH_2), 64.0 (C6), 55.9 (C2), 48.1 (CH_2), 44.6 (C4), 30.8 (Ac-CH_3), 29.0 (CH_2), 23.3 (Ac-CH_3); HRMS *m/z* (ES+) [Found (M+H)+ 571.1847, $C_{27}H_{31}N_4O_8S$ requires M+ 571.1857].

3.1.7 Bis[3-azidopropyl (2-acetamido-2,4-dideoxy)-β-D-glucopyranos-4-yl] disulfide 33

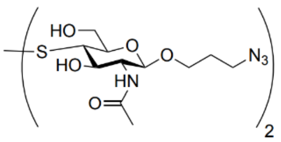

A solution of 4-thioacetate **S7** (72.0 mg, 0.126 mmol, 1.0 equiv.) in MeOH (5 mL) was stirred at RT for 30 mins and NaOMe (7.00 mg, 0.126 mmol, 1.0 equiv.) was then added. The reaction was left stirring at RT until complete consumption of the starting material by TLC (to R_f = 0.49, DCM/MeOH, 9/1). The reaction was neutralised by addition of IR-120(H+) ion exchange resin, filtered and washed with MeOH (100 mL). The filtrate was concentrated under reduced pressure and the crude residue purified by flash chromatography (DCM/MeOH, 0-10%). Compound containing fractions were then lyophilized from H2O (10 mL) to afford **33** as a fluffy white solid (38.0 mg, 0.113 mmol, 46%). R_f = 0.49 (DCM/MeOH, 9/1); $[\alpha]_D^{26}$ = +34.8 ; 1 H NMR (400 MHz, D2O) *δ* 4.41 (d, *J* = 8.4 Hz, 1H, H-1), 4.04 (dd, *J* = 12.3, 1.8 Hz, 1H, H-6b), 3.93 – 3.85 (m, 2H, H-6a, OCHH), 3.75 (t, *J* = 10.2 Hz, 1H, H-3), 3.68 – 3.57 (m, 3H, H-2, H-5, OC*H*H), 3.30 (dd, *J* = 6.6, 1.3 Hz, 2H, CH_2), 2.71 (t, *J* = 10.5 Hz, 1H, H-4), 1.99 (s, 3H, Ac), 1.81 – 1.74 (m, 2H, CH_2); ^{13}C NMR (101 MHz, D2O) δ 174.6 (C=O, Ac), 100.8 (C1), 75.6 (C5), 70.3 (C3), 67.0 (OCH_2), 61.3 (C6), 56.9 (C2), 54.1 (C4), 47.8 (CH_2), 28.1 (CH_2), 22.2 (Ac-CH_3); HRMS *m/z* (ES+) [Found (M+Na)+ 661.2035, $C_{22}H_{38}N_8O_{10}S_2Na$ requires M+ 661.2045].

3.2 Synthesis of 1-(4-(((but-3-yn-1-ylcarbamoyl)oxy)methyl)benzyl)-3-methyl-imidazolium trifluoromethanesulfonate 1.

3.2.1 1-(4-(hydroxymethyl)benzyl)-3-methyl-imidazolium trifluoromethanesulfonate S8.

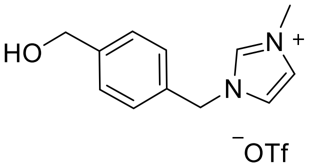

To a stirred solution of 4-(Chloromethyl)benzyl alcohol (2.00 g mL, 12.77 mmol) in anhydrous CH_3CN (50 mL), 1-methylimidazole (4.07 mL, 51.08 mmol) and KOTf (4.69 g, 24.90 mmol) were added. The resulting mixture was stirred for 16 h at reflux (90 °C) under inert atmosphere. The reaction mixture was filtered over a celite pad and the filtrate was concentrated under reduced pressure. The residue was triturated with hexane and Et_2O (3 x 5 mL, Hex:Et_2O 1:1, v/v) to remove the excess of unreacted reagents followed by an initial purification step via silica gel column chromatography to

remove the excess of KOTf (DCM/MeOH 1:0 to 8:2, v:v), furnishing partially purified **S8** as a transparent oil. The oil was then further purified via reverse phase HPLC (MeCN: H_2O) to afford compound **S8** (1.67 g, 37% yield) as colourless oil. **^{1}H NMR** (400 MHz, Methanol-d_4) δ 8.92 (s, 1H, NCHN), 7.56 – 7.52 (m, 2H, $H^{Imidazole}$), 7.42 (s, 4H, H^{Ph}), 5.38 (s, 2H, CH_2N), 4.61 (s, 2H, CH_2O), 3.90(s, 3H, CH_3).**^{13}C NMR** (101 MHz, Methanol-d_4) 142.8, 136.6, 132.6, 128.5, 127.4, 123.8, 122.2, 63.2, 52.5, 35.2. **HRMS** (ESI) m/z: Calcd for $C_{12}H_{15}N_2O^+$ $(M)^+$ 203.1179, found 203.1188.

3.2.2 1-(4-((((4-nitrophenoxy)carbonyl)oxy)methyl)benzyl-3-methyl-imidazolium trifluoromethane sulfonate. S9

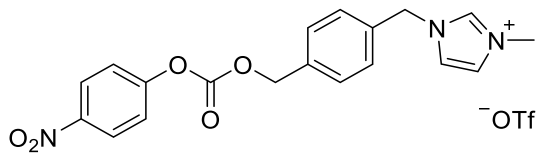

To a stirred solution of **S8** (0.71 g, 2.02 mmol) in a DCM/MeOH solution (14 mL, DCM/MeOH 1:1, v:v) at 0 °C under inert atmosphere, pyridine (0.98 mL, 12.17 mmol) and 4-nitrophenyl chloroformate (1.22 g, 6.06 mmol) were added sequentially and the mixture was stirred for 2.5 hours at room temperature. The reaction mixture was concentrated under reduced pressure and the residue was purified via silica gel column chromatography (DCM/MeOH 1:0 to 9:1, v:v), furnishing **S9** (0.44 g, 41% yield) as a white solid. **^{1}H NMR** (400 MHz, Methanol-d_4) δ 8.99 (d, J = 1.9 Hz, 1H, NCHN), 8.34 – 8.29 (m, 2H, $H^{Nitrophenyl}$), 7.61 (dt, J = 14.8 Hz, J = 1.9 Hz, 2H, $H^{Imidazole}$), 7.56 (d, J = 8.3 Hz, 2H, H^{Ph}), 7.50 (d, J = 2.5 Hz, 2H, H^{Ph}), 7.49 – 7.46 (m, 2H, $H^{Nitrophenyl}$), 5.46 (s, 2H, CH_2N), 5.35 (s, 2H, CH_2O), 3.95 (s, 3H, CH_3). **^{13}C NMR** (101 MHz, Methanol-d_4) δ 155.7, 152.5, 136.2, 134.4, 129.0, 128.6, 124.9, 124.0, 122.3, 121.9, 69.8, 52.3, 35.2. **HRMS** (ESI) m/z: Calcd for $C_{19}H_{18}N_3O_5^+$ $(M)^+$ 368.1241, found 368.1238.

3.2.3 1-(4-(((but-3-yn-1-ylcarbamoyl)oxy)methyl)benzyl)-3-methyl-imidazoliumtrifluoromethane sulfonate 1.

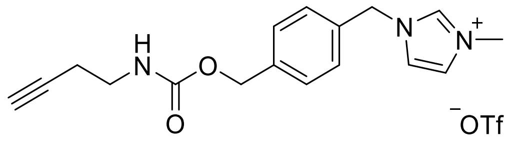

To a stirred solution of **S9** (200 mg, 0.39 mmol) in anhydrous DMF (20 mL) and pyridine (3 mL, 37.24 mmol), 1-amino-3-butyne (122.4 mg, 1.16 mmol) and DIPEA (336 µL, 1.93 mmol) were added and the solution was stirred for 3 hours at 30 °C. The reaction was quenched via the addition of H_2O (1 mL) and the solution was concentrated under reduced pressure. The residue was purified was purified via silica gel column chromatography (DCM/MeOH 1:0 to 9:1, v:v), furnishing **1** (126 mg, 73% yield) as a white solid. **^{1}H NMR** (400 MHz, Methanol-d_4) δ 7.61 (d, J = 2.1 Hz, $1H^{Imidazole}$), 7.58 (d, J = 2.0 Hz, $1H^{Imidazole}$), 7.49 – 7.40 (m, $4H^{Ph}$), 5.42 (s, 2H, $PhCH_2N$), 5.12 (s, 2H,

PhCH_2O), 3.94 (s, 3H, CH_3), 3.26 (t, *J* = 7.0 Hz, 2H, C*H*$_2$CH$_2$C≡CH), 2.37 (td, *J* = 7.0, 2.7 Hz, 2H, CH$_2$C*H*$_2$C≡CH), 2.28 (t, *J* = 2.7 Hz, 1H, CH$_2$CH$_2$C≡C*H*). **^{13}C NMR** (126 MHz, Methanol-*d*$_4$) δ 157.2, 138.5, 133.4, 128.4, 128.1, 123.8, 122.2, 80.7, 69.4, 65.3, 52.3, 39.6, 35.1, 18.9. **HRMS** (ESI) m/z: Calcd for $C_{17}H_{20}N_3O_2^+$ $(M)^+$ 298.1550, found 298.1553.

4. Molecular biology and protein production

4.1 Site directed mutagenesis to make BT1033 D101A mutant.

A PCR reaction was assembled containing 1 X GC Buffer, 200 µM dNTPs, 0.5 µM of each forward & reverse primers, 3% v/v DMSO, 30 ng BT1033_pET24a plasmid DNA and 0.5 units of Phusion polymerase (NEB), with ddH_20 to 25 µL. Following PCR, 1 µl of Dpn1 (NEB) was added to the PCR reaction and incubated at 37 °C for 1 h. 1 µl of Dpn1 treated PCR product was transformed into 50 µL of XL1-Blue Supercompetent cells by heat shock and the cells were grown on LB + Kan (50 µg/mL) at 37 °C for 16 h. To screen for the correct mutation, single colonies were grown in LB + Kan (50 µg/mL) at 37 °C (16 h, 180 rpm), plasmid DNA was isolated by a QIAprep spin miniprep kit (Qiagen) and validated by DNA sequencing (BT1033 D101A_pET24a).

Mutagenic primers for D101A mutation in BT1033

Oligo name	Sequence (5' → 3')
a302c_for (D to A)	CCACGTAACACGCGGGGCATATTTATATTCGGATTC
a302c_rev (D to A)	GAATCCGAATATAAATATGCCCCGCGTGTTACGTGG

PCR conditions for site-directed mutagenesis of BT1033

**Segment**	**Cycles**	**Temperature**	**Time**
1	1	98 °C	30 s
2	35	98 °C	10 s
		62 °C	30 s
		72 °C	3 min
3	1	72 °C	10 min
4	1	4 °C	Hold

4.2 Protein production.

BT1033 and BT1033 D101A were expressed as previously described.[8] Briefly, the BT1033_pET24a (Genbank accession: CP083685.1; purchased from Genscript) and BT1033 D101A pET24a plasmids were each respectively transformed into chemically competent *E. coli* BL21 (DE3) cells by heatshock and plated on LB agar + kanamycin (50 µg/µL). Starter cultures was prepared by picking a single *E. coli* BL21 (DE3) transformant harbouring the expression plasmid into LB + kanamycin (25 µg/µL) and grown at 37 °C 180 rpm for 16 h. The starter culture was diluted 1/100 into 1L of LB + kanamycin (25 µg/µL) in a 3L baffled flask, and grown at 37 °C 180 rpm until it reached an OD_{600} of 0.6. Expression of BT1033 was then induced by adding 0.1 mM isopropyl β-D-thiogalactopyranoside (IPTG) and the cultures were incubated at 18 °C, 180 rpm for 20 h. Cell pellets were resuspended in 50 mL of 50 mM HEPES-NaOH, 500 mM NaCl, pH 7.0 buffer containing pepstatin (final concentration 1 µM), ABDSF (final

concentration 0.2 mM) and benzonase (50 units) and disrupted on ice by sonication (30s on/30s off for 12 min). The supernatant was collected after centrifugation at 20,000 × g (20 min at 4 °C), filtered through a 0.22 µm syringe filter and applied to a HisTrap HP column (5 mL, Cytiva) equilibrated with 50 mM HEPES-NaOH, 500 mM NaCl, 10 mM imidazole pH 7.0 buffer. Proteins were eluted using a linear gradient of 50 mM HEPES-NaOH, 500 mM NaCl, 400 mM imidazole pH 7.0 buffer. The fractions containing recombinant protein were pooled and dialysed into 10 mM HEPES-NaOH, pH 7.0 buffer and concentrated (Sartorius vivaspin6 10,000 MWCO). The recombinant proteins were further purified using a gel filtration column (Hiload 16/600, superdex 200 pg, Cytiva) equilibrated with 10 mM HEPES-NaOH, 150 mM NaCl, pH 7.0 buffer. The fractions containing the recombinant protein were pooled and concentrated (Sartorius vivaspin6 10,000 MWCO). The yield was ~10 mg/L of culture. The activity of WT BT1033 was validated using natural substrates Man-1P and GlcNAc (Section 5.1).

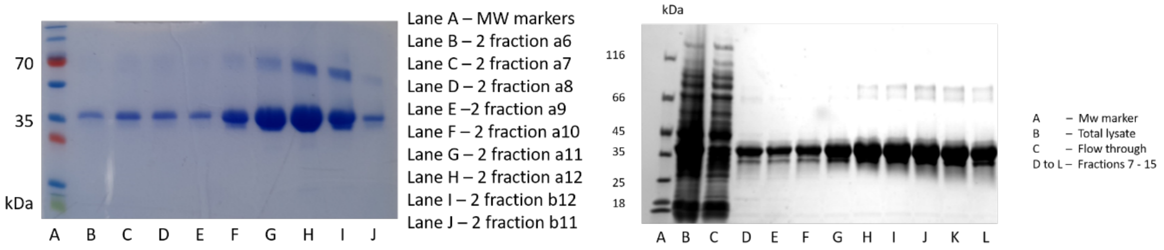

Figure S1. SDS-PAGE analysis of BT1033 (left hand gel) and BT1033 D101A (right hand gel) after Nickel affinity and then gel filtration chromatography.

5. BT1033 activity towards unnatural donors and acceptors

5.1 BT1033 synthetic activity with natural substrates Man-1P & GlcNAc

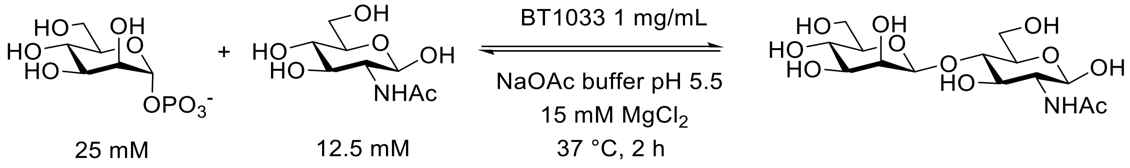

Reactions were prepared on 30 µL scale containing 40 mM NaOAc pH 5.5 buffer, 25 mM Man-1P (donor), 12.5 mM GlcNAc (acceptor), 15 mM $MgCl_2$ and BT1033 (1 mg/mL). Samples were incubated at 37 °C for 2 h. 1 µL of sample was diluted in 30 µL of MeCN and analysed by HILIC-LC-MS to validate the desired product formation (Figure S2).

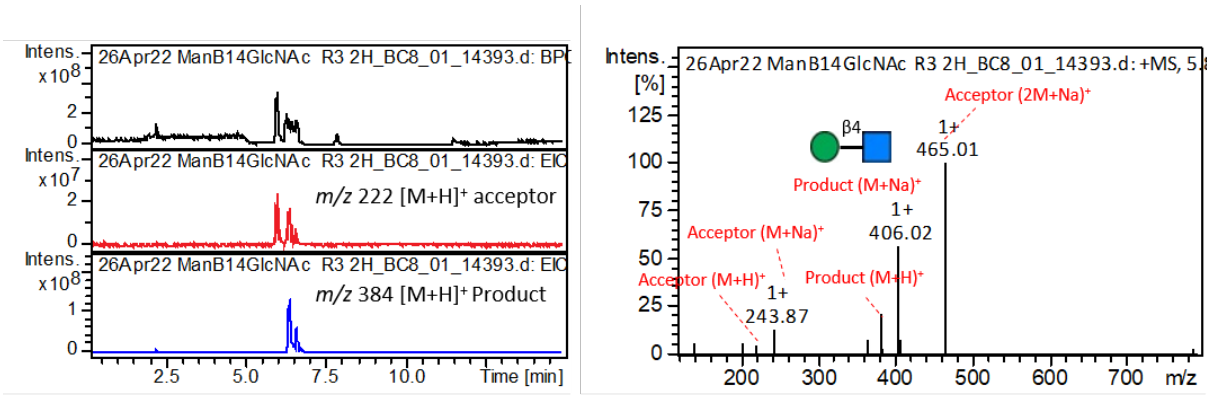

Figure S2. BT1033 synthetic activity validated with natural substrates Man-1P & GlcNAc. Right-hand panel: Base peak chromatogram (BPC) shown in black and extracted ion chromatograms (EIC) for unreacted acceptor GlcNAc at *m/z* 222 $(M+H)^+$ (red) and product Manβ1,4GlcNAc *m/z* 384 $(M+H)^+$ (blue). Left hand panel: average mass spectrum taken over the acceptor and product peaks.

5.2 BT1033 synthetic activity screen with unnatural mannose-1-phosphates

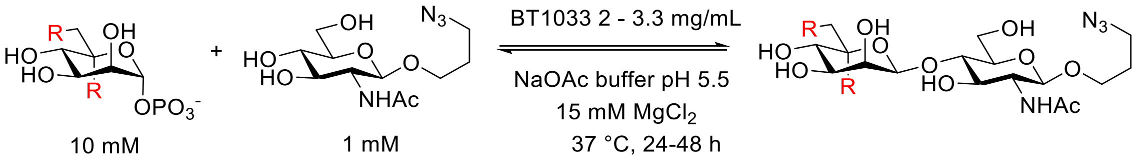

Reactions were prepared in duplicate on a 30 µL scale containing 40 mM NaOAc pH 5.5 buffer, 10 mM sugar phosphate donor, 1 mM acceptor, 15 mM $MgCl_2$ and BT1033 (2-3.3 mg/mL). Samples were incubated at 37 °C for 24 h. Specific reaction conditions for each analogue are listed in Table S1. 3 µL of each sample was diluted in 30 µL of MeCN and analysed by HILIC-LC-MS to validate product formation. The remaining sample was diluted 1:1 in 40 mM NaOAc pH 5.5 buffer and then further

diluted 1:1 in EtOH and stored at – 20 °C for 18 h. Samples were centrifuged at 17 000 x g for 5 min and the supernatant collected. 1 mL of H_2O was added to each sample and the samples were lyophilised. The samples were labelled with the ITag (procedure in section 7) and analysed by C_{18}-LC-MS. (Figure S3-S10, Table S1).

Table S1. Screening conditions and results summary of BT1033 activity towards unnatural Man-1P donors.

Disaccharide product	Donor concentration (mM)	Acceptor concentration (mM)	BT-1033 concentration (µg/µL)	Incubation time (h)	Mean conversion to disaccharide (%)
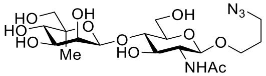	10	1	3.3	48	51 ± 0.6
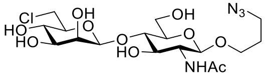	10	1	2	24	61 ± 7.2
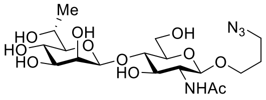	10	1	3.3	24	44 ± 0.7
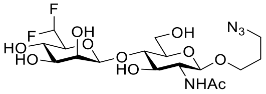	10	1	2	24	0.4 ± 0.1
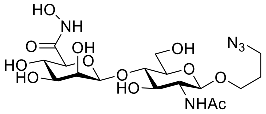	10	1	2	24	0.26 ± 0.1
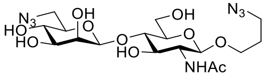	10	1	2	24	11 ± 0.1
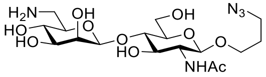	10	1	2	24	0.4 ± 0.1
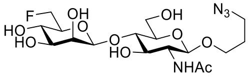	10	1	2	24	16 ± 2.1

± Standard deviation

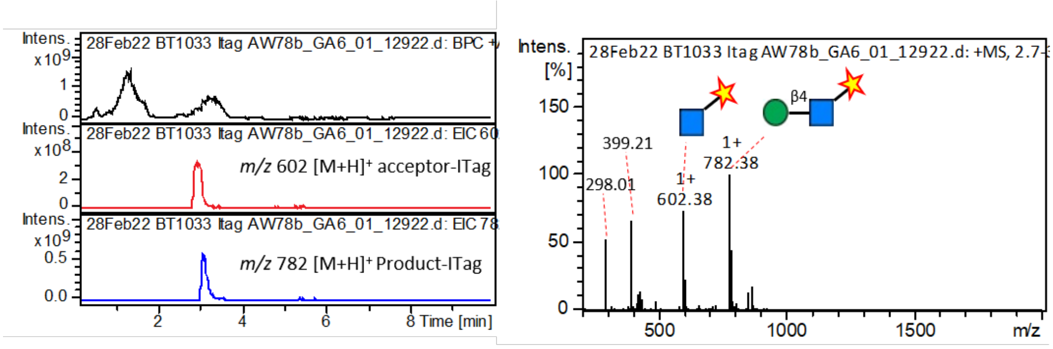

Figure S3. BT1033 synthetic activity validated with substrates C6-Chloro Man-1P **4** & GlcNAc-N_3 **11**. Right-hand panel: BPC shown in black and EIC for unreacted acceptor GlcNAc-N_3 at *m/z* 602 $(M+H)^+$ (red) and product *m/z* 782 $(M+H)^+$ (blue). Left hand panel: average mass spectrum taken over the acceptor and product peaks. Peak at *m/z* = 298 is unreacted ITag. Peak at *m/z* = 399 is a molecular ion fragment.

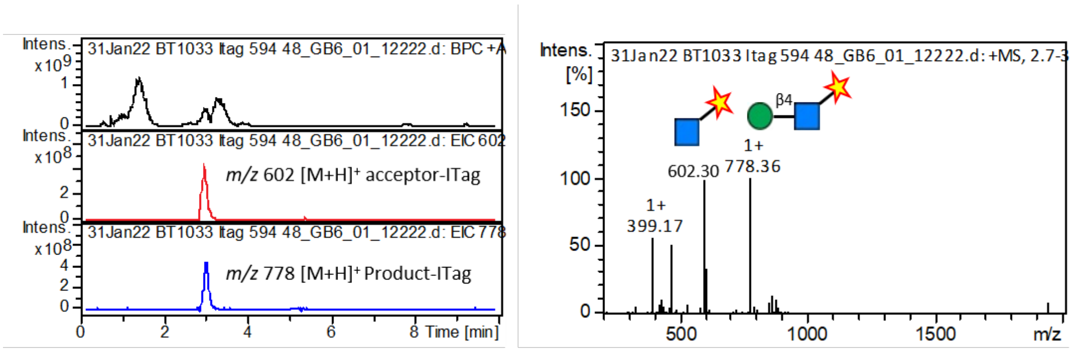

Figure S4. BT1033 synthetic activity validated with substrates C5-methyl Man-1P **3** & GlcNAc-N_3 **11**. Right-hand panel: BPC shown in black and EIC for unreacted acceptor GlcNAc-N_3 at *m/z* 602 $(M+H)^+$ (red) and product *m/z* 778 $(M+H)^+$ (blue). Left hand panel: average mass spectrum taken over the acceptor and product peaks. Peak at *m/z* = 399 is a molecular ion fragment.

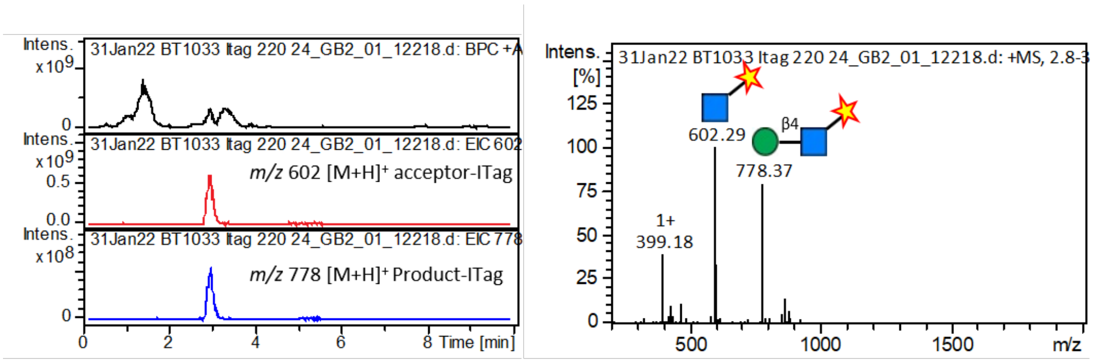

Figure S5. BT1033 synthetic activity validated with substrates C6-methyl Man-1P **5** & GlcNAc-N_3 **11**. Right-hand panel: BPC shown in black and EIC for unreacted acceptor GlcNAc-N_3 at *m/z* 602 $(M+H)^+$ (red) and product *m/z* 778 $(M+H)^+$ (blue). Left hand panel: average mass spectrum taken over the acceptor and product peaks. Peak at *m/z* = 399 is a molecular ion fragment.

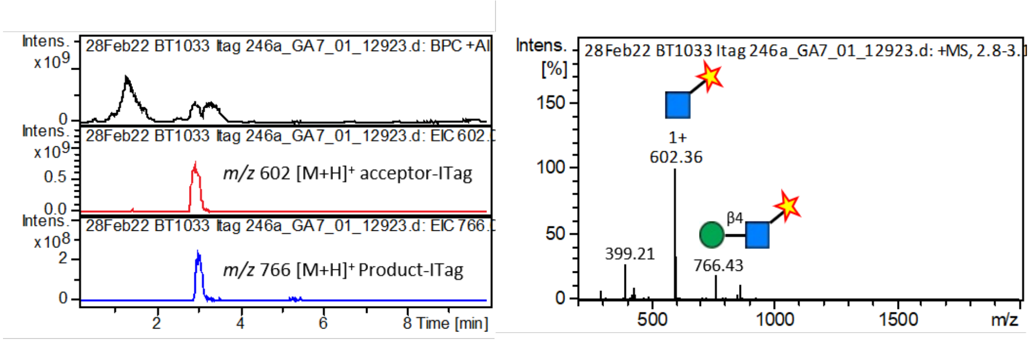

Figure S6. BT1033 synthetic activity validated with substrates C6-fluoro Man-1P **6** & GlcNAc-N_3 **11**. Right-hand panel: BPC shown in black and EIC for unreacted acceptor GlcNAc-N_3 at *m/z* 602 $(M+H)^+$ (red) and product *m/z* 766 $(M+H)^+$ (blue). Left hand panel: average mass spectrum taken over the acceptor and product peaks. Peak at *m/z* = 399 is a molecular ion fragment.

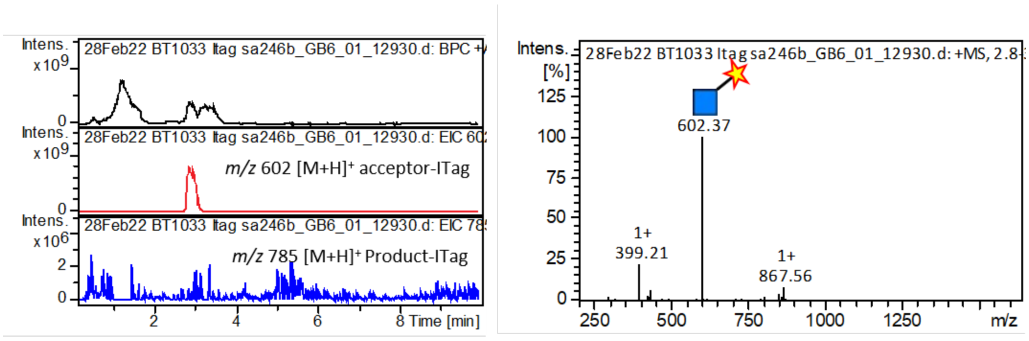

Figure S7. BT1033 synthetic activity validated with substrates C6-gem-difluoro Man-1P **8** & GlcNAc-N_3 **11**. Right-hand panel: BPC shown in black and EIC for unreacted acceptor GlcNAc-N_3 at *m/z* 602 $(M+H)^+$ (red) and product *m/z* 785 $(M+H)^+$ (blue). Left hand panel: average mass spectrum taken over the acceptor and product peaks. Peak at *m/z* = 399 is a molecular ion fragment.

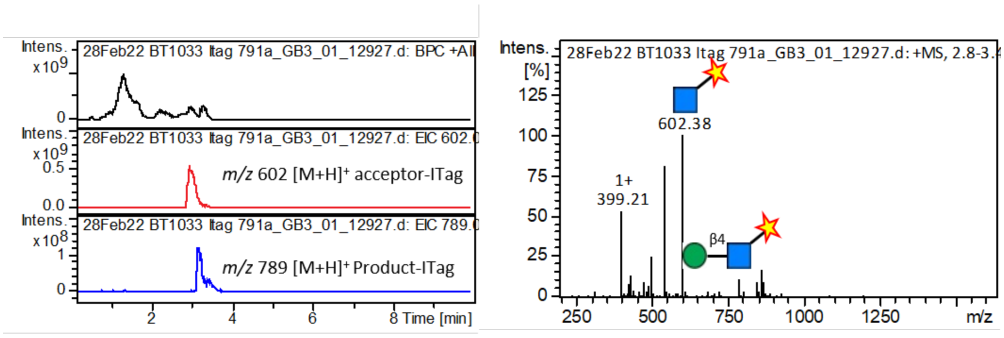

Figure S8. BT1033 synthetic activity validated with substrates C6-azido Man-1P **7** & GlcNAc-N_3 **11**. Right-hand panel: BPC shown in black and EIC for unreacted acceptor GlcNAc-N_3 at *m/z* 602 $(M+H)^+$ (red) and product *m/z* 789 $(M+H)^+$ (blue). Left hand panel: average mass spectrum taken over the acceptor and product peaks. Peak at *m/z* = 399 is a molecular ion fragment.

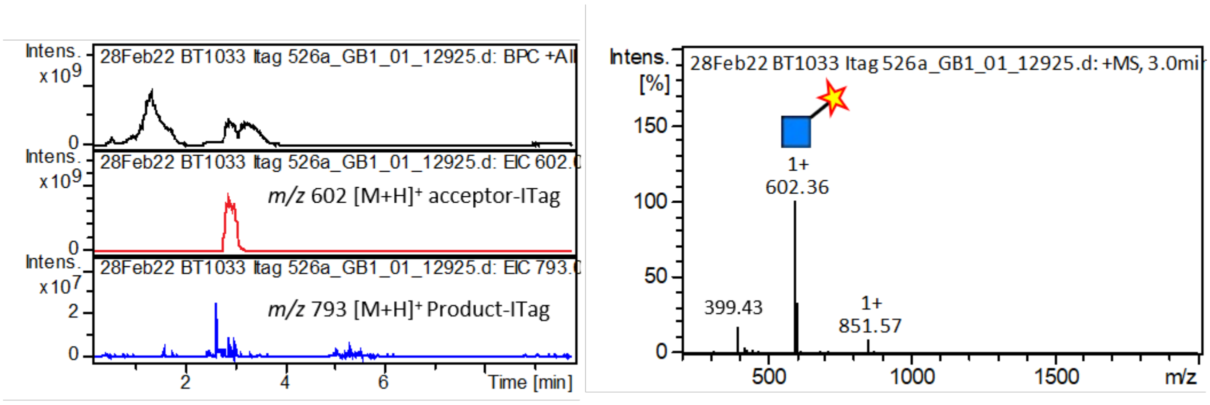

Figure S9. BT1033 synthetic activity validated with substrates C6-hydroxamic acid Man-1P **9** & GlcNAc-N_3 **11**. Right-hand panel: BPC shown in black and EIC for unreacted acceptor GlcNAc-N_3 at *m/z* 602 $(M+H)^+$ (red) and product *m/z* 793 $(M+H)^+$ (blue). Left hand panel: average mass spectrum taken over the acceptor and product peaks. Peak at *m/z* = 399 is a molecular ion fragment.

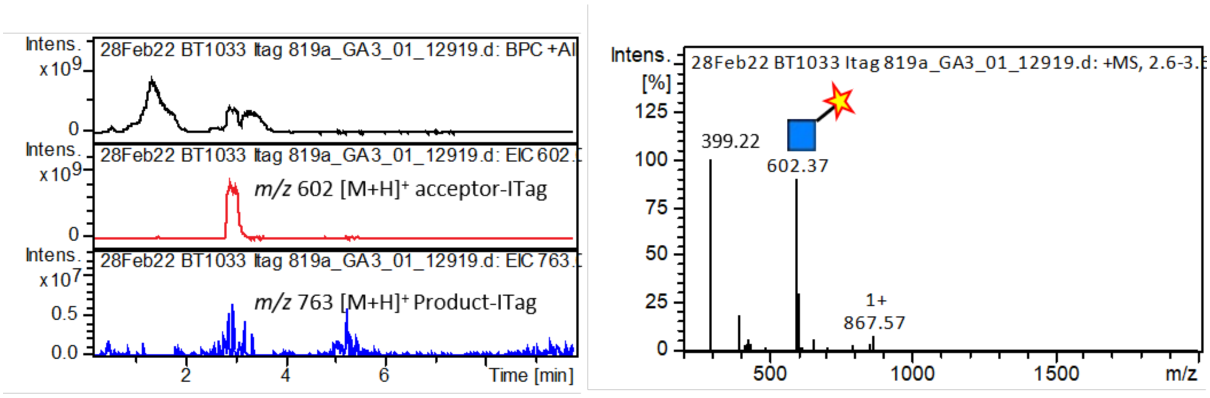

Figure S10. BT1033 synthetic activity validated with substrates C6-amine Man-1P **10** & GlcNAc-N_3 **11**. Right-hand panel: BPC shown in black and EIC for unreacted acceptor GlcNAc-N_3 at *m/z* 602 $(M+H)^+$ (red) and product *m/z* 763 $(M+H)^+$ (blue). Left hand panel: average mass spectrum taken over the acceptor and product peaks. Peak at *m/z* = 399 is a molecular ion fragment.

5.3 BT1033 synthetic activity screen with unnatural acceptors

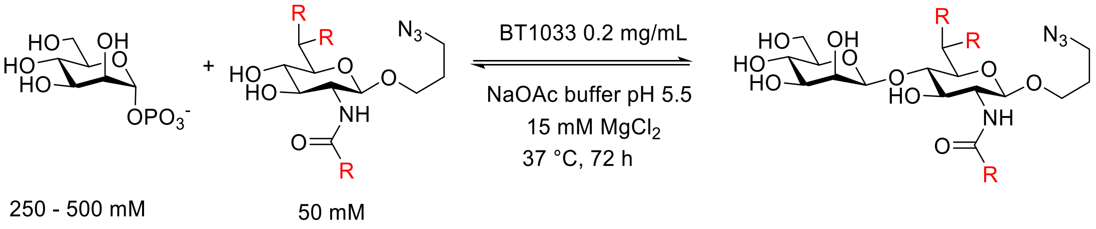

Reactions were prepared in duplicate on a 10 µL scale containing 40 mM NaOAc pH 5.5 buffer, 250 - 500 mM Man1-P **2** donor, 50 mM acceptor, 15 mM $MgCl_2$ and BT1033 (0.2 mg/mL). Samples were incubated at 37 °C for 72 h. Specific reaction conditions for each analogue are listed in Table S2. 3 µL of each sample was diluted in 30 µL of MeCN and analysed by HILIC-LC-MS to validate product formation. The remaining sample was diluted 1:1 in 40 mM NaOAc pH 5.5 buffer and then further diluted 1:1 in EtOH and stored at – 20 °C for 18 h. Samples were centrifuged at 17 000 x g for 5 min and the supernatant collected. 1 mL of H_2O was added to each sample and the samples were lyophilised. The samples were labelled with the ITag (see procedure in section 7) and analysed by C_{18}-LC-MS. (Table S2, Figure S11-S14).

Table S2. Screening conditions and results summary of BT1033 activity towards unnatural GlcNAc acceptors.

Disaccharide product	Donor concentration (mM)	Acceptor concentration (mM)	BT-1033 concentration (μg/μL)	Incubation time (h)	Conversion to disaccharide (%)	Conversion to trisaccharide (%)	Conversion to tetrasaccharide (%)	Conversion to pentasaccharide (%)
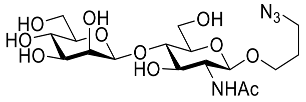	500	50	0.2	72	74 ± 1.6	4 ± 0.4		
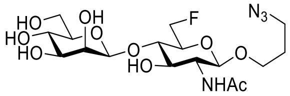	500	50	0.2	72	41 ± 0.4	41 ± 0.6	1 ± 0.04	
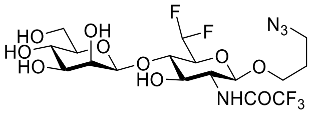	500	50	0.2	72	2 ± 0.01			
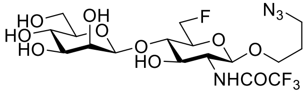	250	50	0.2	72	22± 6.1	19 ± 5.1	9 ± 2.4	2 ± 0.6

± Standard deviation

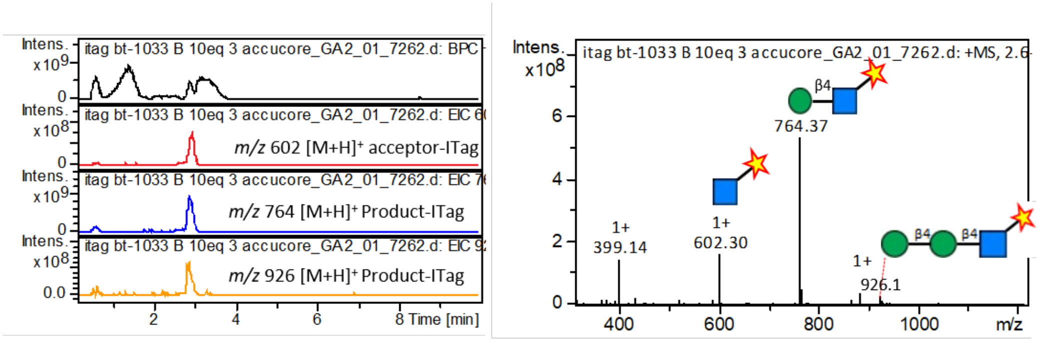

Fig S11. BT1033 synthetic activity validated with substrates Man-1P **2** & GlcNAc-N_3 **11.** Right-hand panel: BPC (black) and EIC shown for I-tagged unreacted acceptor (*m/z* 602, red), disaccharide (*m/z* 764, blue) and trisaccharide (*m/z* 926, orange). Left hand panel: average mass spectrum taken over the acceptor and product peaks.

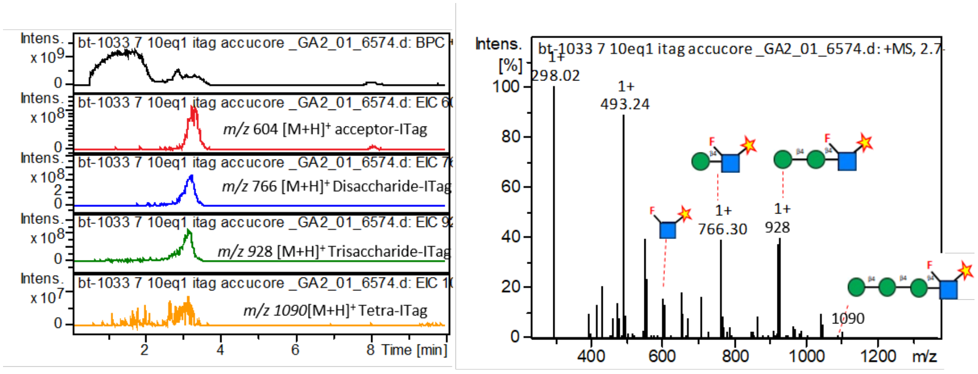

Fig S12. BT1033 synthetic activity validated with substrates Man-1P 2 & 6F-GlcNAc-N_3 **12**. Right-hand panel: BPC (black) and EIC shown for I-tagged unreacted acceptor (*m/z* 604, red), disaccharide (*m/z* 766, blue), trisaccharide (*m/z* 928, green) and tetrasaccharide (*m/z* 1090, orange). Left hand panel: average mass spectrum taken over the acceptor and product peaks.

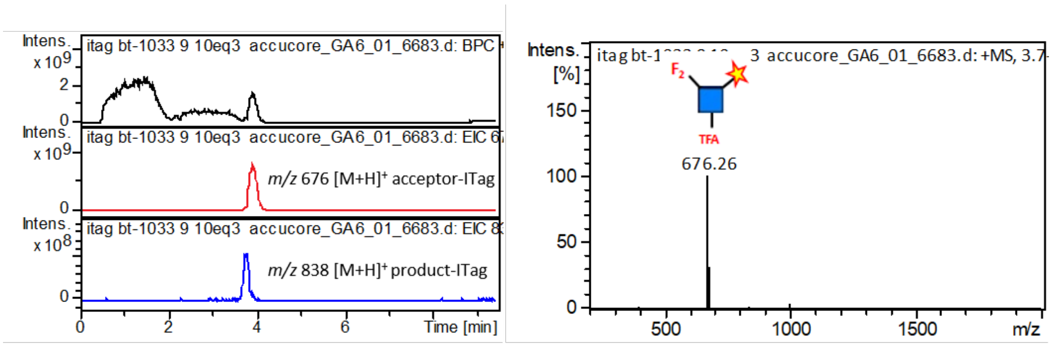

Fig S13. BT1033 synthetic activity validated with substrates Man-1P **2** & 6,6-diFGlcNTFA-N_3 **13.** Right-hand panel: BPC (black) and EIC shown for I-tagged unreacted acceptor (*m/z* 676, red) and disaccharide (*m/z* 838, blue). Left hand panel: average mass spectrum taken over the acceptor and product peaks.

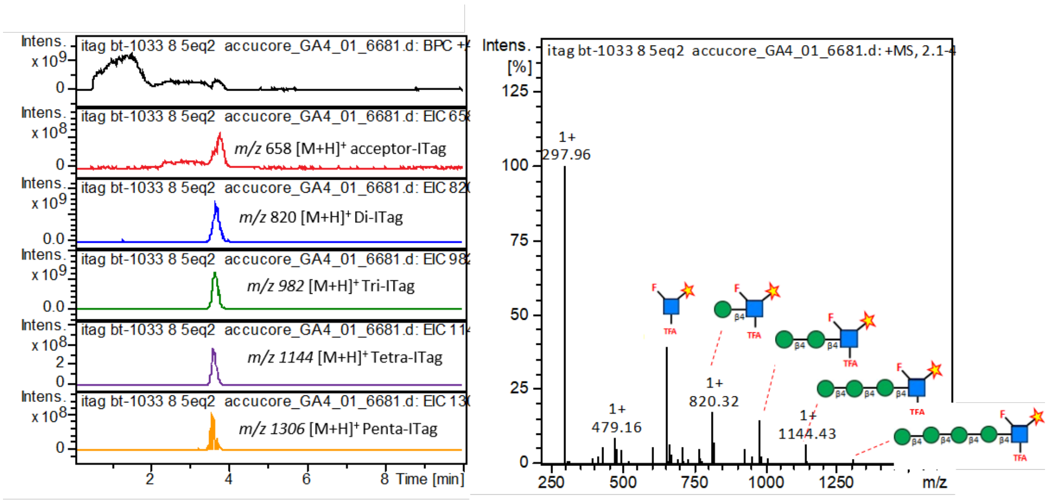

Fig S14. BT1033 synthetic activity validated with substrates Man-1P **2** & 6F-GlcNTFA-N_3 **14.** Right-hand panel: BPC (black) and EIC shown for I-tagged unreacted acceptor (*m/z* 658, red), disaccharide (*m/z* 820, blue), trisaccharide (*m/z* 982, green), tetrasaccharide (*m/z* 1144, purple) and pentasaccharide (*m/z* 1306, orange). Left hand panel: average mass spectrum taken over the acceptor and product peaks.

5.4 Investigating thioglycolygase activity of WT BT1033 and BT1033 D101A

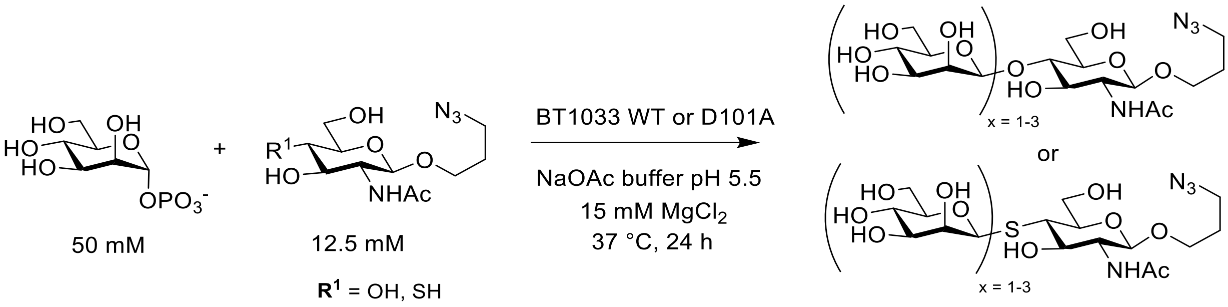

Reactions were prepared on a 30 µL scale containing 40 mM NaOAc pH 5.5 buffer, 50 mM sugar phosphate donor, 12.5 mM SH-GlcNAc-N_3 **33,** 5 mM TCEP (added from a 100 mM stock made in 40 mM NaOAc buffer and adjusted to pH 5.5), 15 mM $MgCl_2$ and WT BT1033 or BT1033 D101A (0.2 mg/mL or 0.4 mg/mL). Control reactions were assembled under the same conditions, except with GlcNAc-N_3 **11** as the acceptor. Samples were incubated at 37 °C for 24 h. 3 µL of each sample was diluted in 30 µL of MeCN and analysed by HILIC-LC-MS to validate product formation. The remaining sample was diluted 1:1 in 40 mM NaOAc pH 5.5 buffer and then further diluted 1:1 in EtOH and stored at – 20 °C for 18 h. Samples were centrifuged at 17 000 x g for 5 min and the supernatant collected. 1 mL of H_2O was added to each sample and the samples were lyophilised. The samples were labelled with the ITag (procedure outlined in section 7) and analysed by C_{18}-LC-MS. (Figure S15-S22).

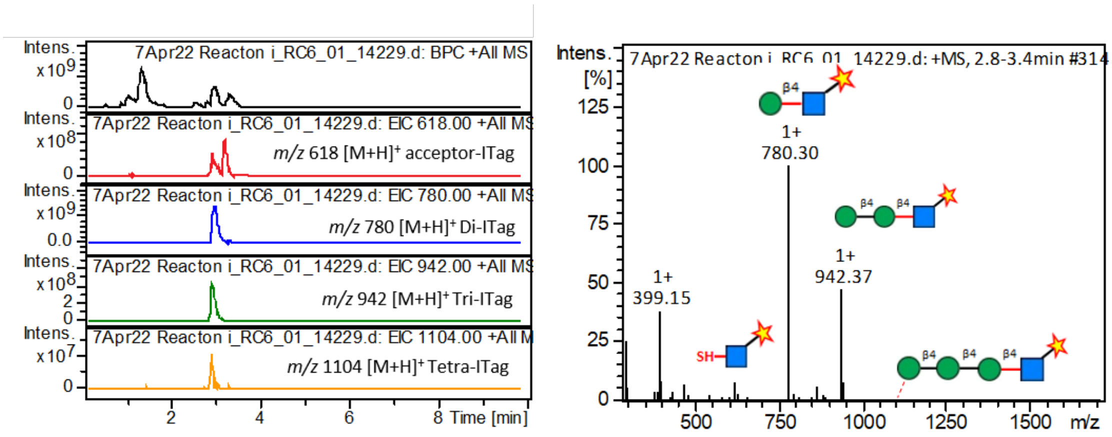

Fig S15. WT BT1033 (0.2 mg/mL) synthetic activity investigated under reducing conditions with substrates Man-1P **2** & SH-GlcNAc-N_3 **33.** Right-hand panel: BPC (black) and EIC shown for I-tagged unreacted acceptor (*m/z* 618, red), disaccharide (*m/z* 780, blue), trisaccharide (*m/z* 942, green) and tetrasaccharide (*m/z* 1104, orange). Left hand panel: average mass spectrum taken over the acceptor and product peaks.

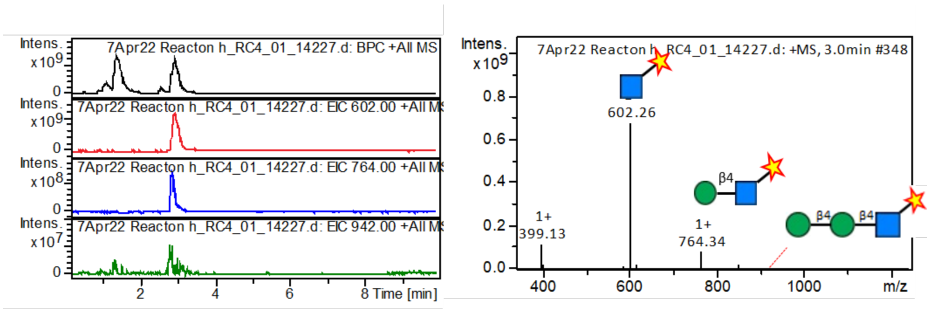

Fig S16. WT BT1033 (0.2 mg/mL) synthetic activity investigated under reducing conditions with substrates Man-1P **2** & GlcNAc-N_3 **11**. Right-hand panel: BPC (black) and EIC shown for I-tagged unreacted acceptor (*m/z* 602, red), disaccharide (*m/z* 764, blue) and trisaccharide (*m/z* 942, green). Left hand panel: average mass spectrum taken over the acceptor and product peaks.

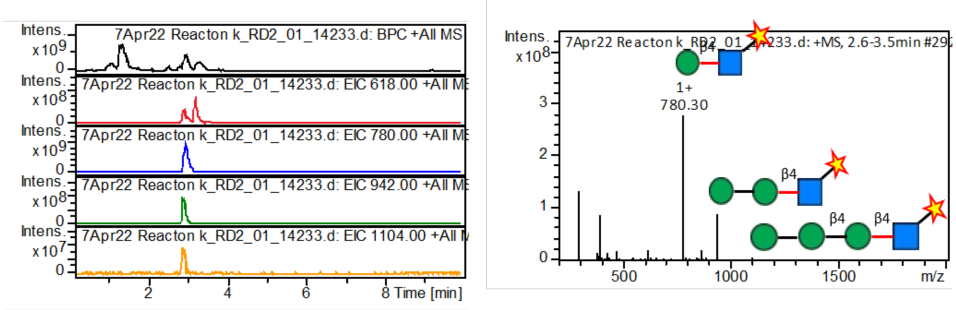

Fig S17. WT BT1033 (0.4 mg/mL) synthetic activity investigated under reducing conditions with substrates Man-1P **2** & SH-GlcNAc-N_3 **33.** Right-hand panel: BPC (black) and EIC shown for I-tagged unreacted acceptor (*m/z* 618, red), disaccharide (*m/z* 780, blue), trisaccharide (*m/z* 942, green) and tetrasaccahride (*m/z* 1104, orange). Left hand panel: average mass spectrum taken over the acceptor and product peaks.

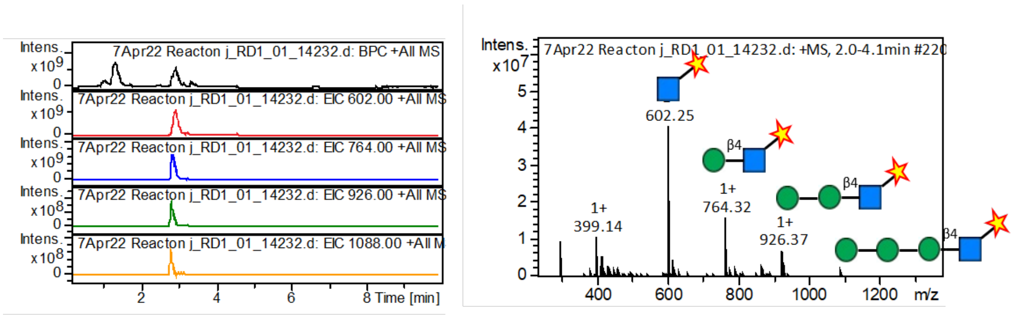

Fig S18. WT BT1033 (0.4 mg/mL) synthetic activity investigated under reducing conditions with substrates Man-1P **2** & GlcNAc-N_3 **11**. Right-hand panel: BPC (black) and EIC shown for I-tagged unreacted acceptor (*m/z* 602, red), disaccharide (*m/z* 764, blue), trisaccharide (*m/z* 926, green) and tetrasaccahride (*m/z* 1088, orange). Left hand panel: average mass spectrum taken over the acceptor and product peaks.

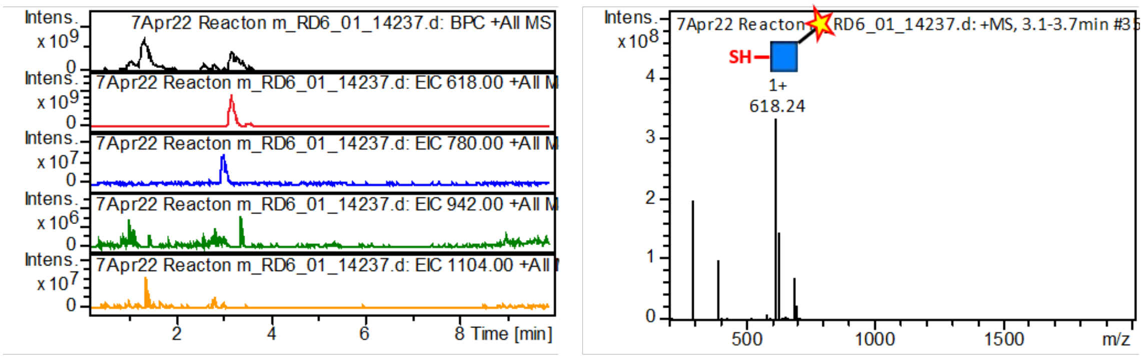

Fig S19. D101A BT1033 (0.2 mg/mL) synthetic activity investigated under reducing conditions with substrates Man-1P **2** & SH-GlcNAc-N_3 **33.** Right-hand panel: BPC (black) and EIC shown for I-tagged unreacted acceptor (*m/z* 618, red), disaccharide (*m/z* 780, blue), trisaccharide (*m/z* 942, green) and tetrasaccahride (*m/z* 1104, orange). Left hand panel: average mass spectrum taken over the acceptor and product peaks.

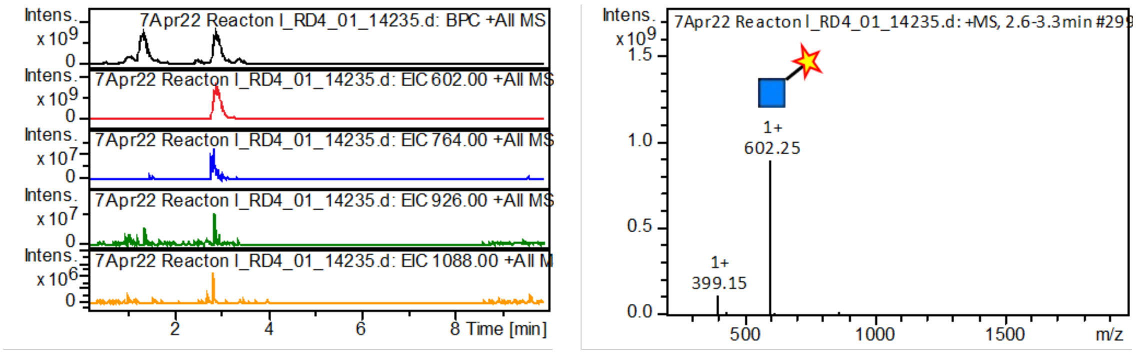

Fig S20. D101A BT1033 (0.2 mg/mL) synthetic activity investigated under reducing conditions with substrates Man-1P **2** & GlcNAc-N_3 **11**. Right-hand panel: BPC (black) and EIC shown for I-tagged unreacted acceptor (*m/z* 602, red), disaccharide (*m/z* 764, blue), trisaccharide (*m/z* 926, green) and tetrasaccahride (*m/z* 1088, orange). Left hand panel: average mass spectrum taken over the acceptor and product peaks.

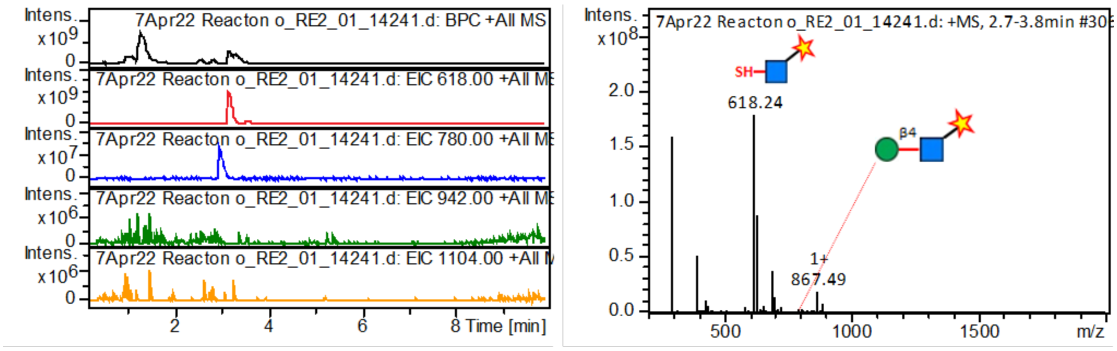

Fig S21. D101A BT1033 (0.4 mg/mL) synthetic activity investigated under reducing conditions with substrates Man-1P **2** & SH-GlcNAc-N_3 **33.** Right-hand panel: BPC (black) and EIC shown for I-tagged unreacted acceptor (*m/z* 618, red), disaccharide (*m/z* 780, blue), trisaccharide (*m/z* 942, green) and tetrasaccahride (*m/z* 1104, orange). Left hand panel: average mass spectrum taken over the acceptor and product peaks.

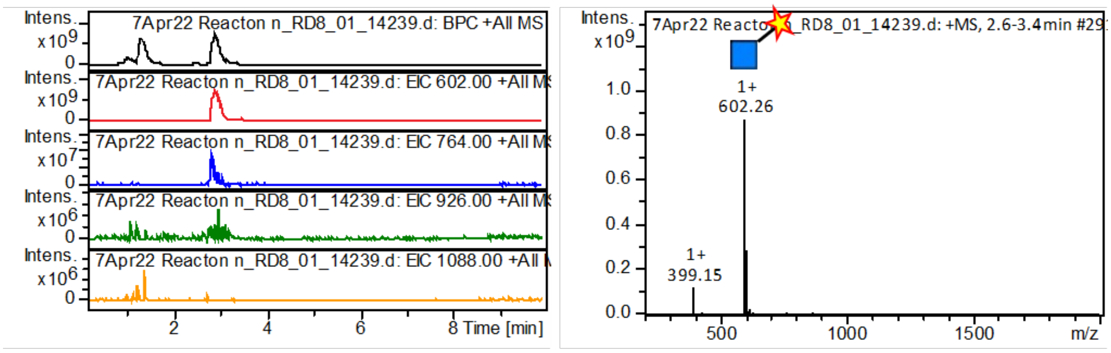

Fig S22. D101A BT1033 (0.4 mg/mL) synthetic activity investigated under reducing conditions with substrates Man-1P **2** & GlcNAc-N_3 **11**. Right-hand panel: BPC (black) and EIC shown for I-tagged unreacted acceptor (*m/z* 602, red), disaccharide (*m/z* 764, blue), trisaccharide (*m/z* 926, green) and tetrasaccahride (*m/z* 1088, orange). Left hand panel: average mass spectrum taken over the acceptor and product peaks.

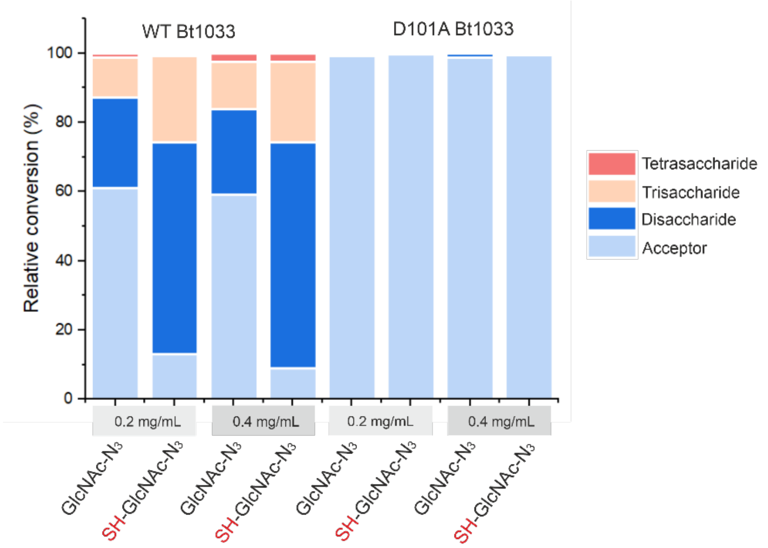

Fig. S23 Summary of BT1033 and BT1033 D101A activity towards GlcNAc-N_3 **11** and SH-GlcNAc-N_3 **33**. The data for BT1033 turnover of GlcNAc-N_3 **11** and SH-GlcNAc-N_3 **33** at 0.2 mg/mL (the first 2 bars shown here) are presented in Figure 3C in the main text.

6. BT1033 phosphorylysis of synthetic β-mannosides

Reactions were prepared on a 10 µL scale containing 40 mM NaOAc pH 5.5 buffer, 5 mM sugar, 15 mM $MgCl_2$, 10 mM KH_2PO_4 (prepared freshly for each set of reactions) and BT1033 (0.1 mg/mL). No enzyme controls were assembled alongside each reaction to monitor hydrolysis over the time course. Samples were incubated at 37 °C for 20 h. Reactions and controls were stopped at 2 min and 20 h, after the addition of 10 µL of EtOH. Samples were stored at – 20 °C for 18 h. Samples were centrifuged at 17 000 x g for 5 min and the supernatant collected. 1 mL of H_2O was added to each sample and the samples were lyophilised. The samples were labelled with the ITag (procedure outlined in section 7) and analysed by C_{18}-LC-MS (Figure S24-S33).

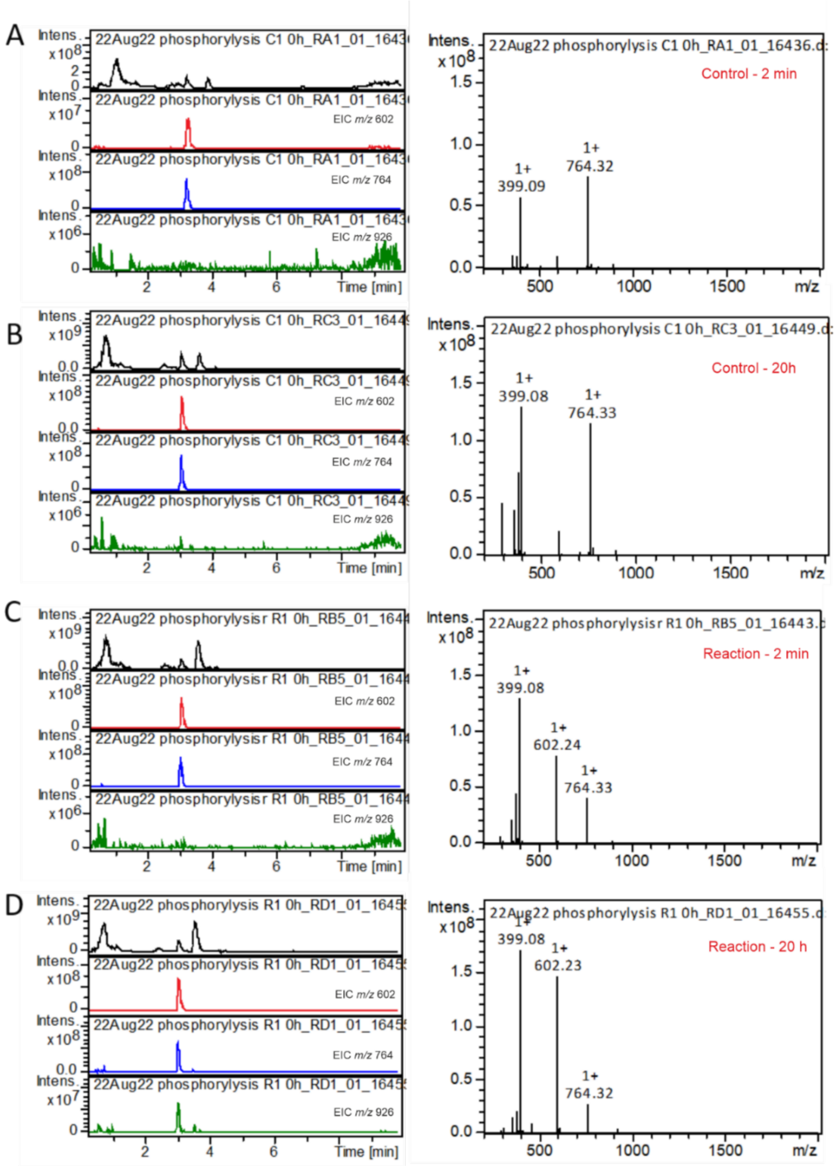

Fig S24. Phosphorolysis time course of Manβ1,4-GlcNAc-N_3 **15.** Right-hand panels: BPC (black) and EIC shown for I-tagged unreacted acceptor (*m/z* 602, red), disaccharide (*m/z* 764, blue) and trisaccharide (*m/z* 926, green). Left hand panels: average mass spectrum taken over the acceptor and product peaks. A. Control reaction at 2 min. B. Control reaction at 20 h. C. Reaction at 2 min. D. Reaction after 20 h.

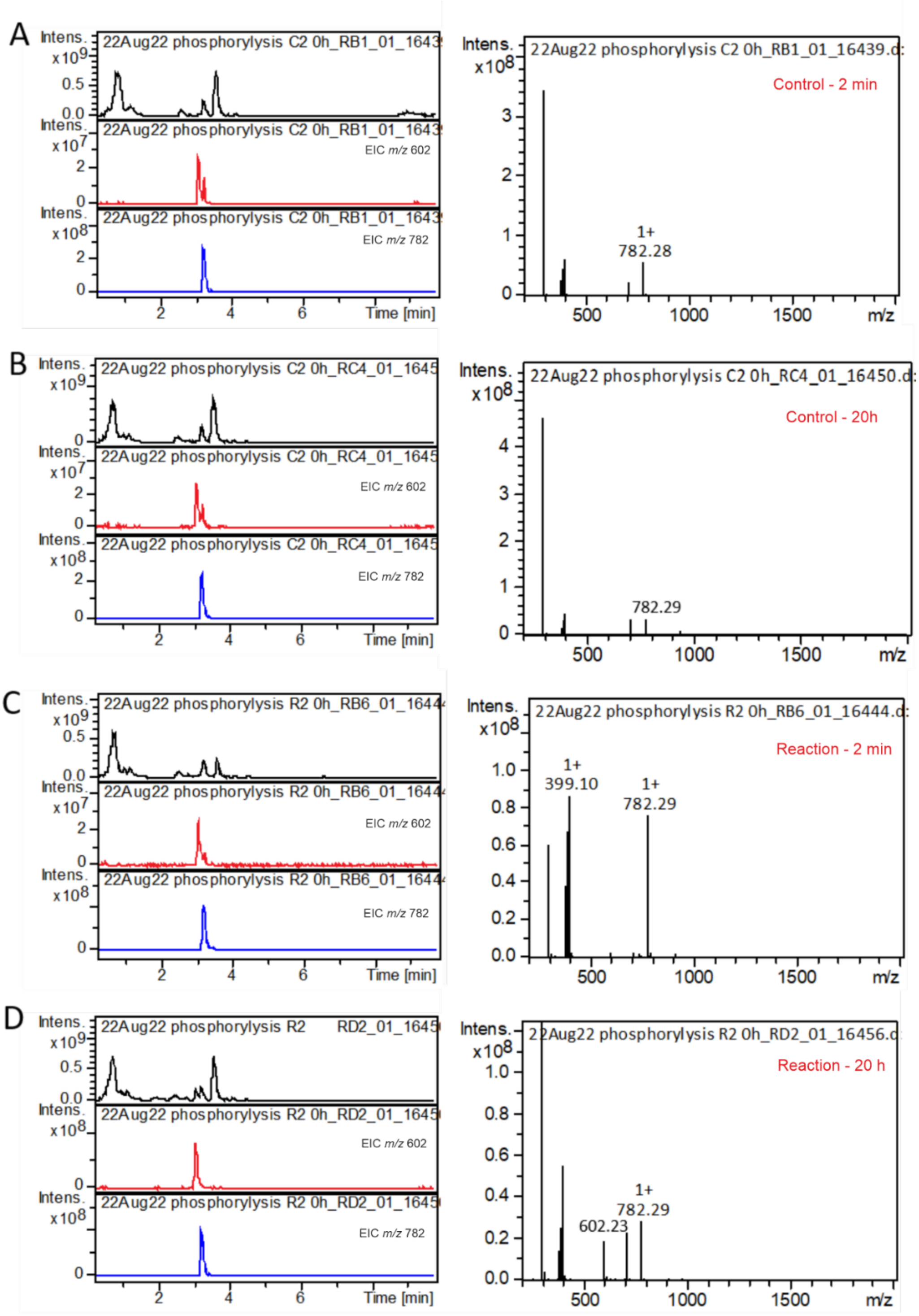

Fig S25. Phosphorolysis time course of 6Cl-Manβ1,4-GlcNAc-N_3 **18.** Right-hand panels: BPC (black) and EIC shown for I-tagged unreacted acceptor (*m/z* 602, red) and disaccharide (*m/z* 782, blue). Left hand panels: average mass spectrum taken over the acceptor and product peaks. A. Control reaction at 2 min. B. Control reaction at 20 h. C. Reaction at 2 min. D. Reaction after 20 h.

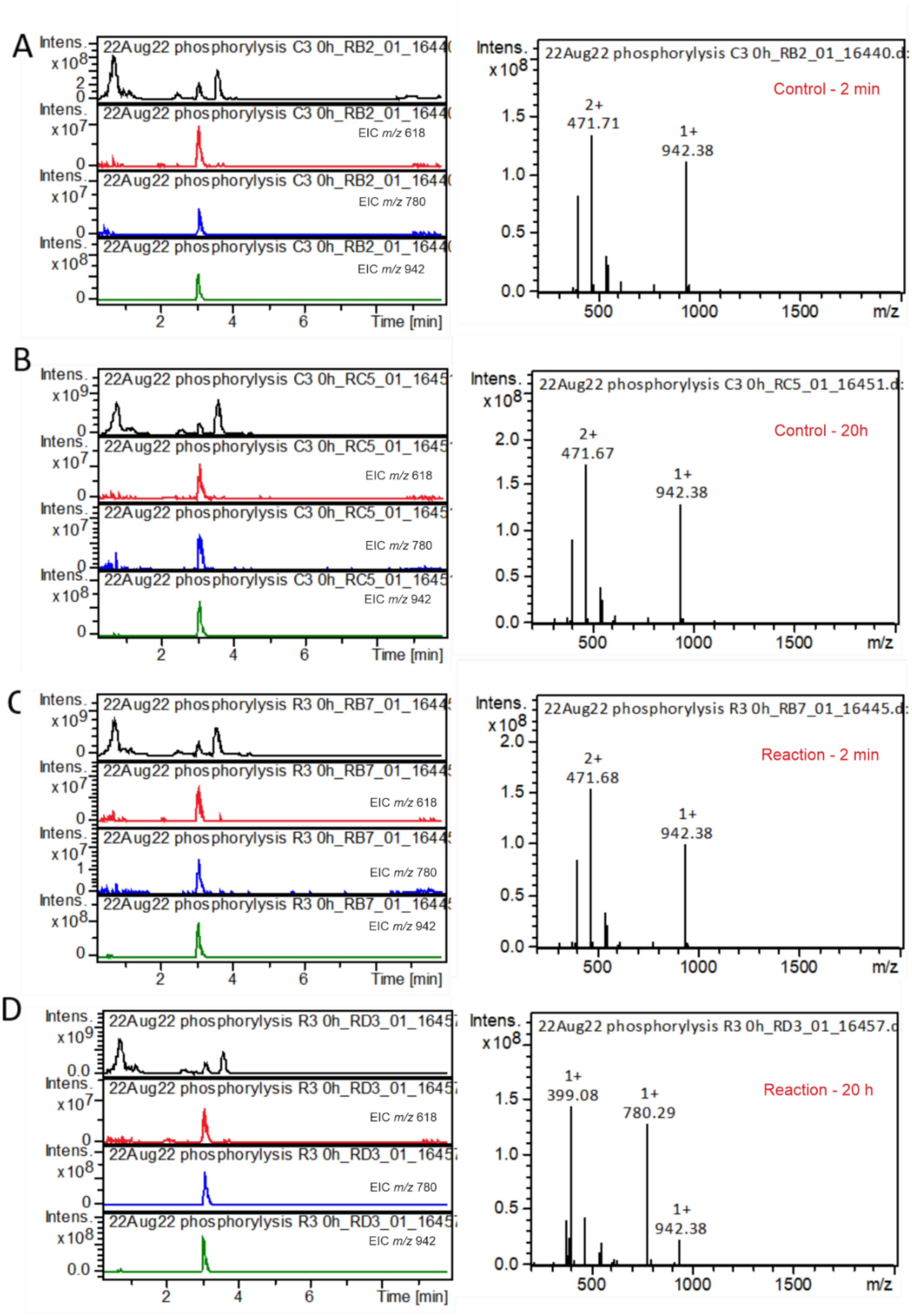

Fig S26. Phosphorolysis time course of Manβ1,4-Manβ1,4-S-GlcNAc-N_3 **35.** Right-hand panels: BPC (black) and EIC shown for I-tagged unreacted acceptor (*m/z* 602, red), disaccharide (*m/z* 782, blue) and trisaccharide (*m/z* 942, green). Left hand panels: average mass spectrum taken over the acceptor and product peaks. A. Control reaction at 2 min. B. Control reaction at 20 h. C. Reaction at 2 min. D. Reaction after 20 h.

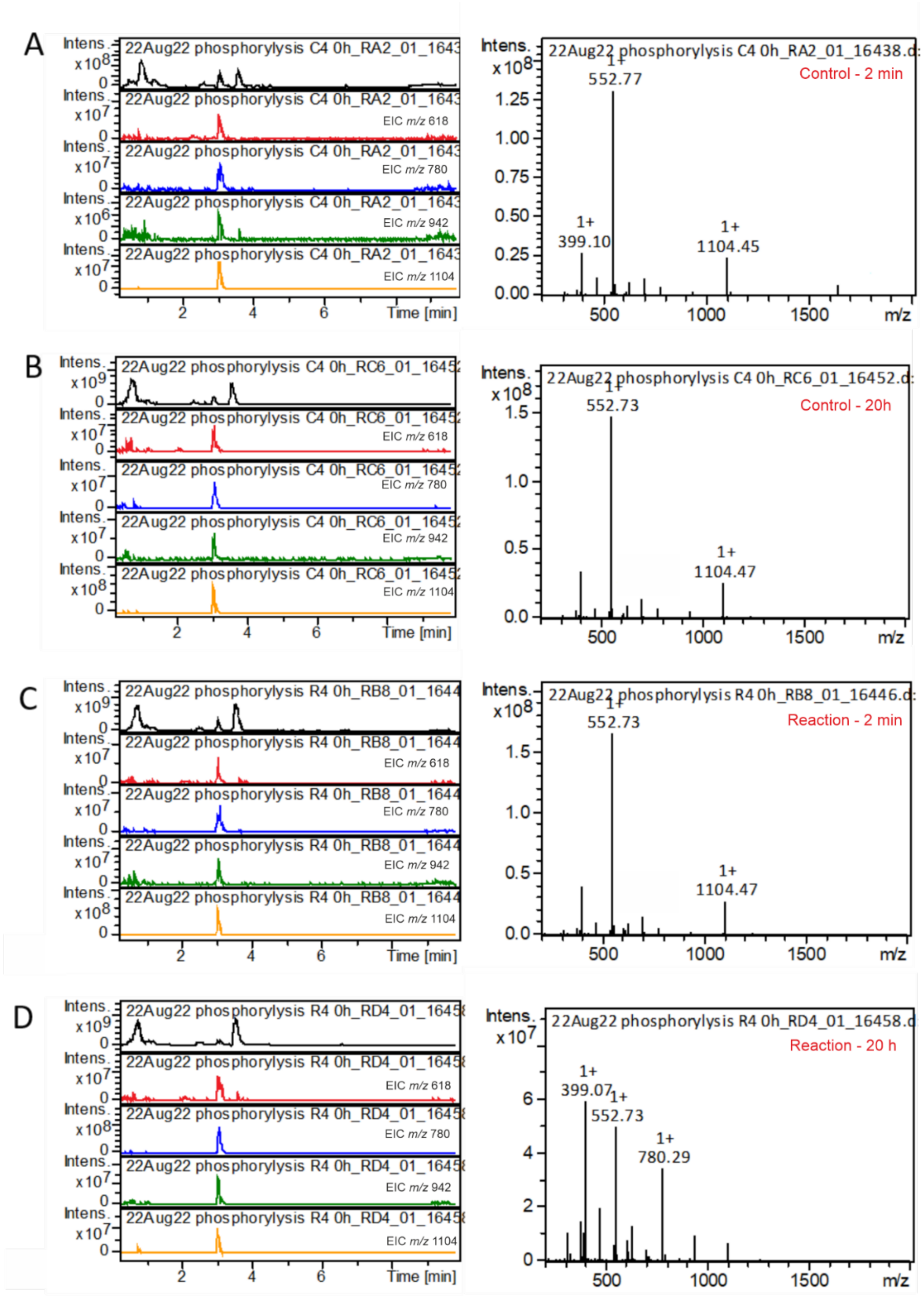

Fig S27. Phosphorolysis time course of Manβ1,4-Manβ1,4-Manβ1,4-S-GlcNAc-N_3 **36.** Right-hand panels: BPC (black) and EIC shown for I-tagged unreacted acceptor (*m/z* 602, red), disaccharide (*m/z* 782, blue), trisaccharide (*m/z* 942, green) and tetrasaccharide (*m/z* 1104, orange). Left hand panels: average mass spectrum taken over the acceptor and product peaks. A. Control reaction at 2 min. B. Control reaction at 20 h. C. Reaction at 2 min. D. Reaction after 20 h.

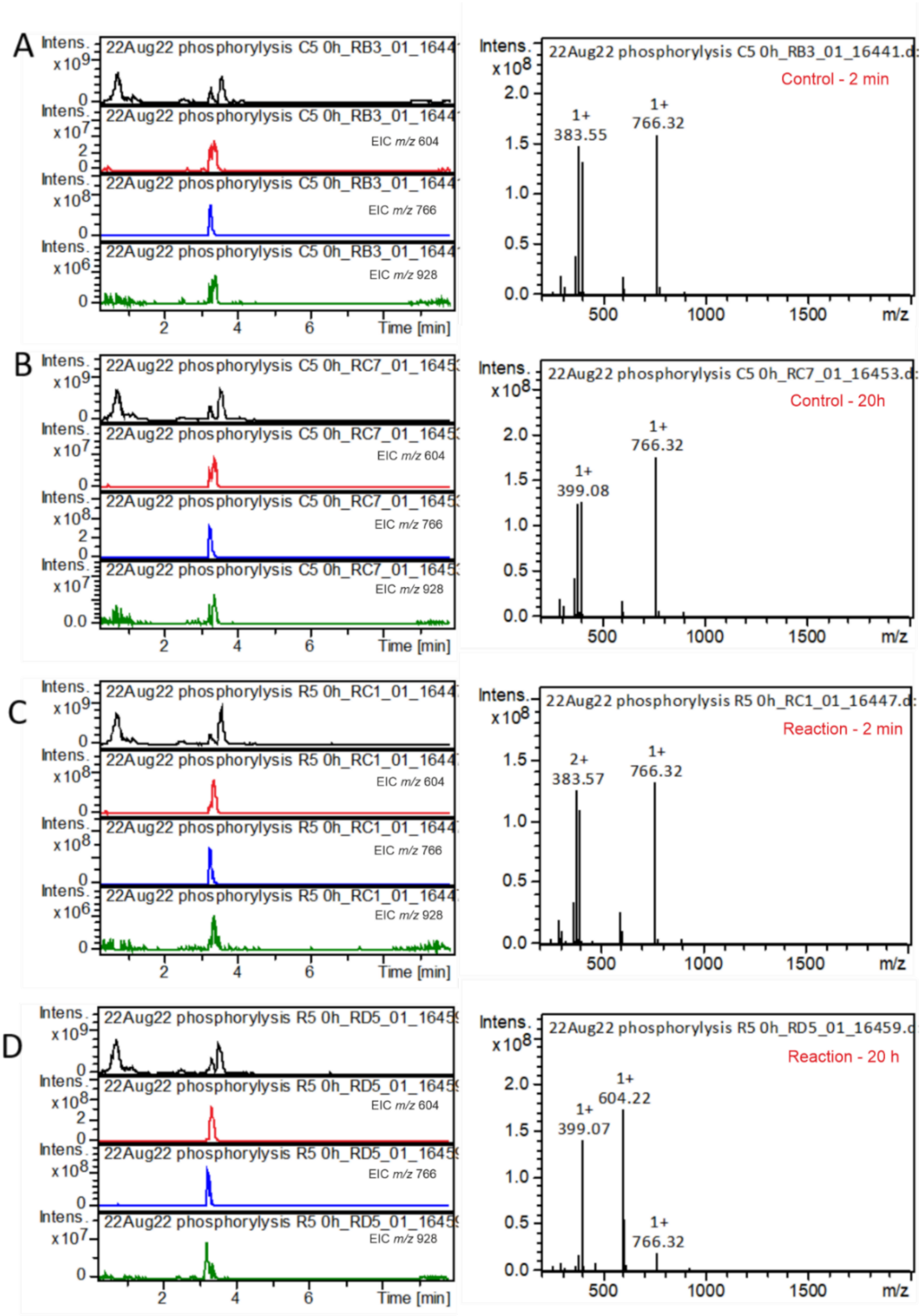

Fig S28. Phosphorolysis time course of Manβ1,4-6F-GlcNAc-N_3 **25.** Right-hand panels: BPC (black) and EIC shown for I-tagged unreacted acceptor (*m/z* 604, red), disaccharide (*m/z* 766, blue) and trisaccharide (*m/z* 928, green). Left hand panels: average mass spectrum taken over the acceptor and product peaks. A. Control reaction at 2 min. B. Control reaction at 20 h. C. Reaction at 2 min. D. Reaction after 20 h.

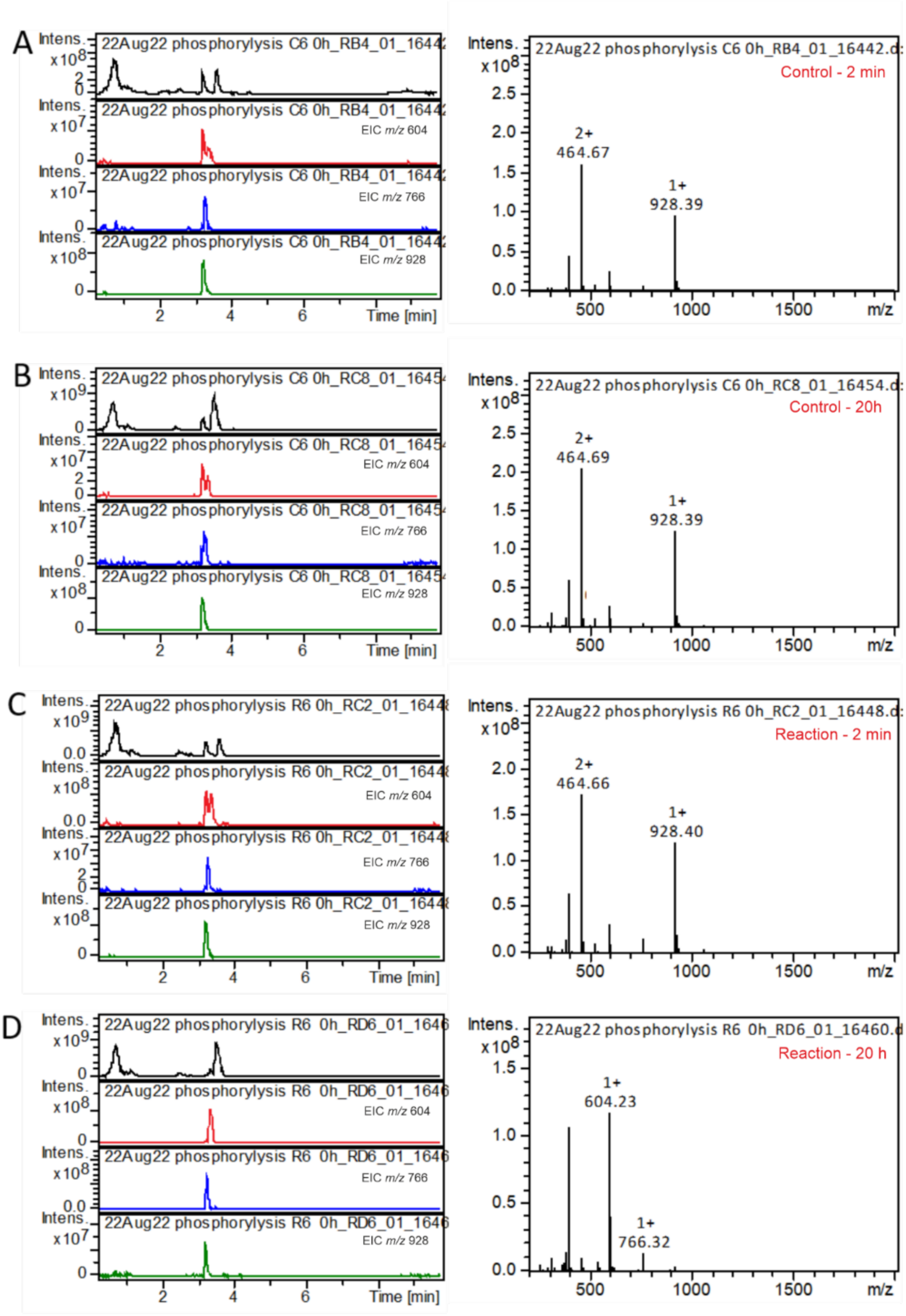

Fig S29. Phosphorolysis time course of Manβ1,4-Manβ1,4-6F-GlcNAc-N_3 **26.** Right-hand panels: BPC (black) and EIC shown for I-tagged unreacted acceptor (*m/z* 604, red), disaccharide (*m/z* 766, blue) and trisaccharide (*m/z* 928, green). Left hand panels: average mass spectrum taken over the acceptor and product peaks. A. Control reaction at 2 min. B. Control reaction at 20 h. C. Reaction at 2 min. D. Reaction after 20 h.

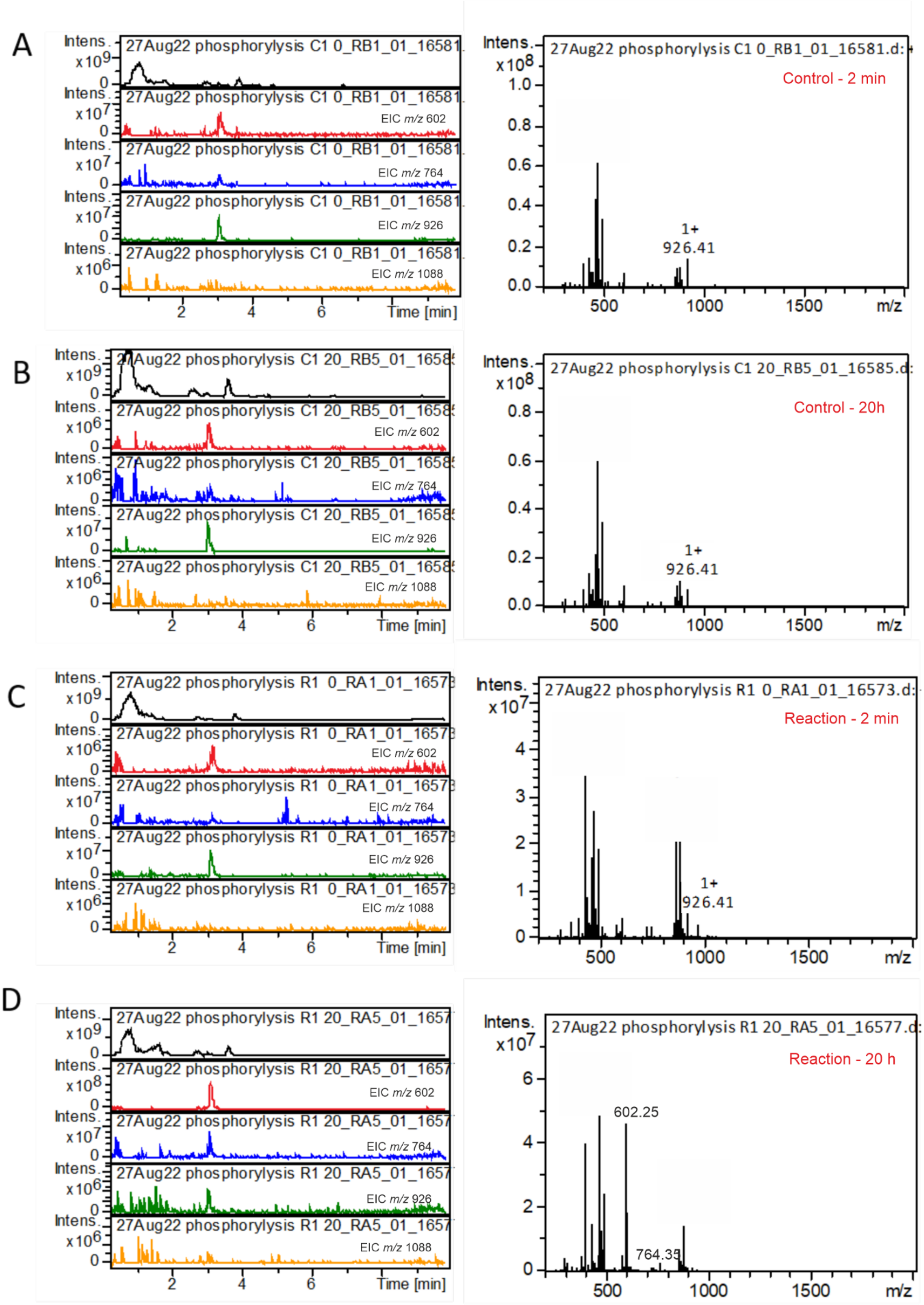

Fig S30. Phosphorolysis time course of Manβ1,4-Manβ1,4-GlcNAc-N_3 **16.** Right-hand panels: BPC (black) and EIC shown for I-tagged unreacted acceptor (*m/z* 602, red), disaccharide (*m/z* 764, blue), trisaccharide (*m/z* 926, green) and tetrasaccharide (*m/z* 1088, orange). Left hand panels: average mass spectrum taken over the acceptor and product peaks. A. Control reaction at 2 min. B. Control reaction at 20 h. C. Reaction at 2 min. D. Reaction after 20 h.

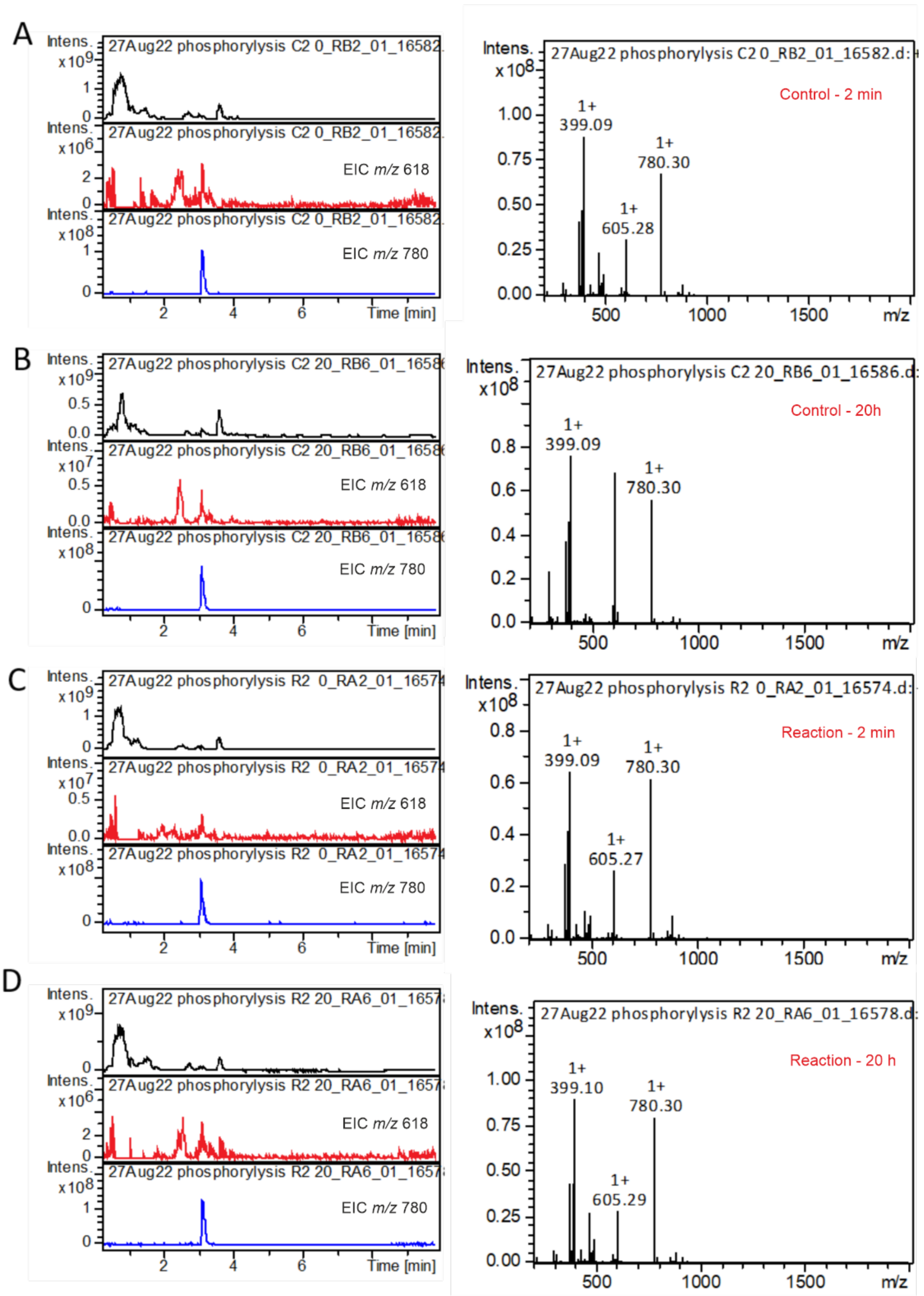

Fig S31. Phosphorolysis time course of Manβ1,4-S-GlcNAc-N_3 **34.** Right-hand panels: BPC (black) and EIC shown for I-tagged unreacted acceptor (*m/z* 618, red) and disaccharide (*m/z* 780, blue). Left hand panels: average mass spectrum taken over the acceptor and product peaks. A. Control reaction at 2 min. B. Control reaction at 20 h. C. Reaction at 2 min. D. Reaction after 20 h.

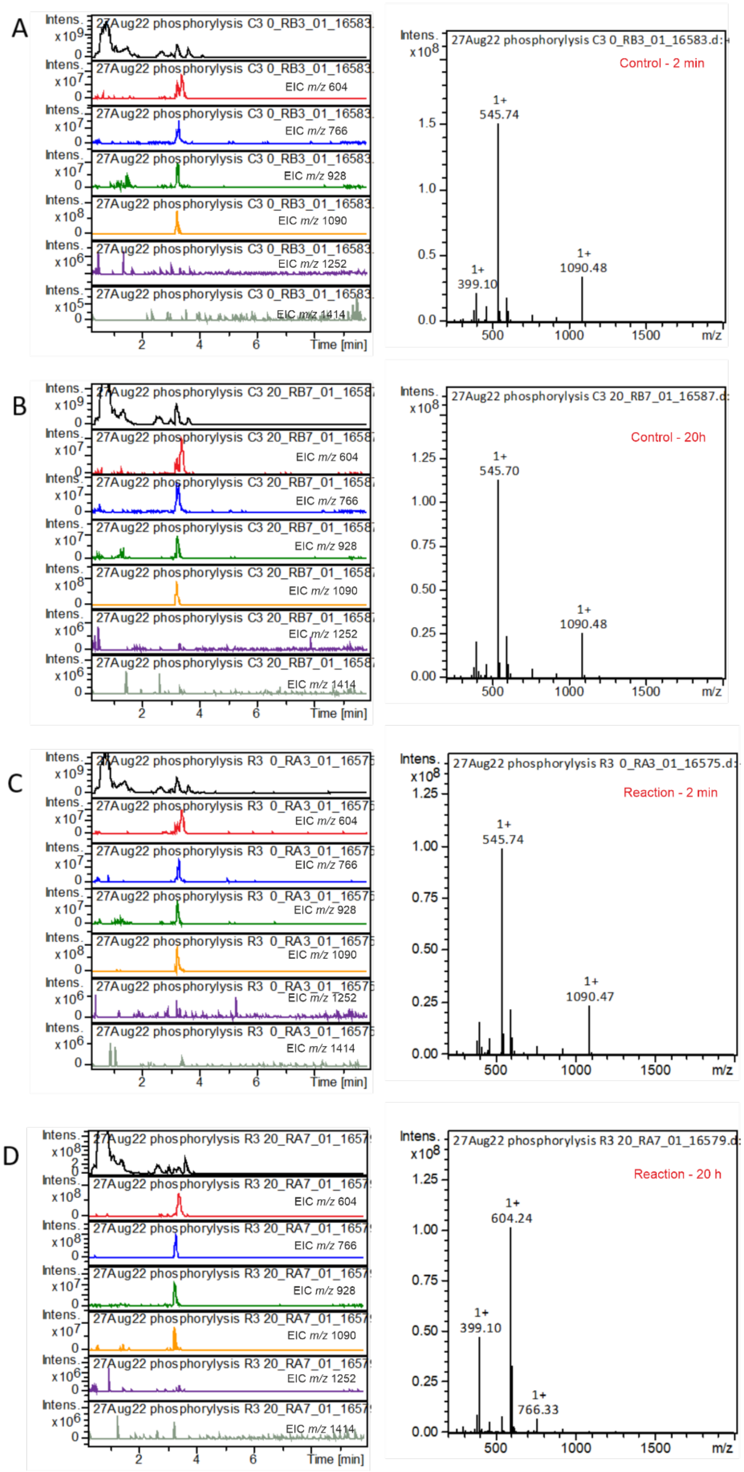

Fig S32. Phosphorolysis time course of Manβ1,4-Manβ1,4-Manβ1,4-6F-GlcNAc-N_3 **27.** Right-hand panels: BPC (black) and EIC shown for I-tagged unreacted acceptor (*m/z* 604, red), disaccharide (*m/z* 766, blue), trisaccharide (*m/z* 928, green), tetrasaccharide (*m/z* 1090, orange), pentasaccharide (*m/z* 1252, purple) and hexasaccharide (*m/z* 1414, grey). Left hand panels: average mass spectrum taken over the acceptor and product peaks. A. Control reaction at 2 min. B. Control reaction at 20 h. C. Reaction at 2 min. D. Reaction after 20 h.

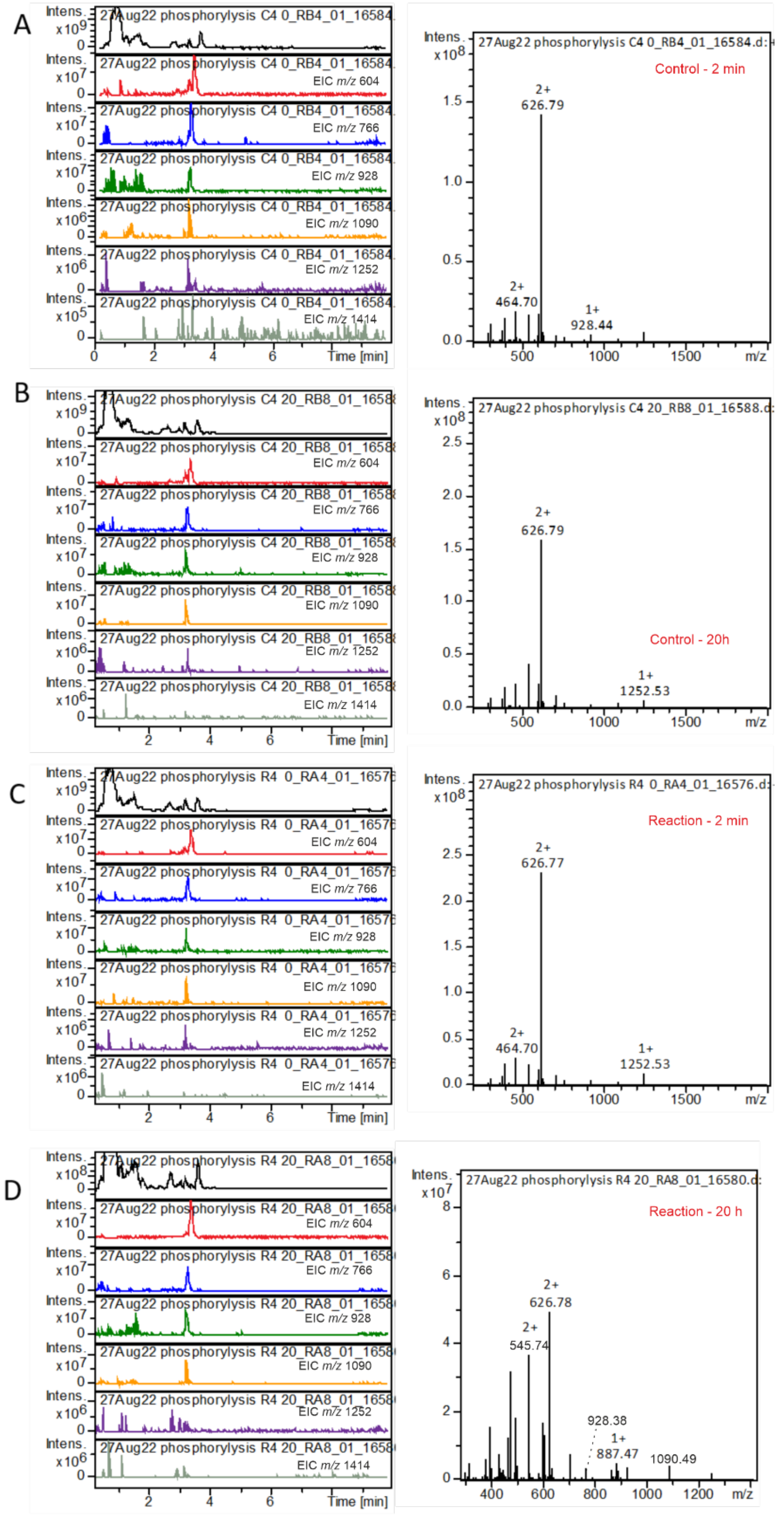

Fig S33. Phosphorolysis time course of Manβ1,4-Manβ1,4-Manβ1,4-Manβ1,4-6F-GlcNAc-N_3 **37.** BPC (black) and EIC shown for I-tagged unreacted acceptor (*m/z* 604, red), disaccharide (*m/z* 766, blue), trisaccharide (*m/z* 928, green), tetrasaccharide (*m/z* 1090, orange), pentasaccharide (*m/z* 1252, purple) and hexasaccharide (*m/z* 1414, grey). Left hand panels: average mass spectrum taken over the acceptor and product peaks. A. Control reaction at 2 min. B. Control reaction at 20 h. C. Reaction at 2 min. D. Reaction after 20 h.

7. ITag labelling of phosphorylase reaction products.

Lyophilised samples were resuspended in 24 µL of H_2O, then 0.5 - 1 µL of Itag solution (250 mM in DMSO) was added to each sample. Then 10 µL of a stock solution containing 500 mM $CuSO_4$. 5 H_2O and 500 mM THPTA in H_2O, was added to each sample. Samples were mixed by vortexing and then 5 µL of a 1 M sodium ascorbate solution was added to each sample. Samples were further mixed by vortexing and then incubated for 2 h at 20 °C 300 rpm. 1 mL of H_2O was added to each sample and the samples were lyophilised. Samples were resuspended in 100 µL of H_2O. and analysed by C_{18}-LC-MS. Relative conversion of starting material to products was determined by analysing the peak intensities of starting material and product species, and using the following equation:

$$\frac{\text{Product peak intensity}}{\text{Starting material peak intensity + Product peak intensity}} \quad \text{x} \quad 100 = \text{\% relative conversion}$$

8. Semi-preparative production of β-mannosides

8.1 Synthesis of Manβ1,4-GlcNAc-N_3 15 and Man_2β1,4-GlcNAc-N_3 16

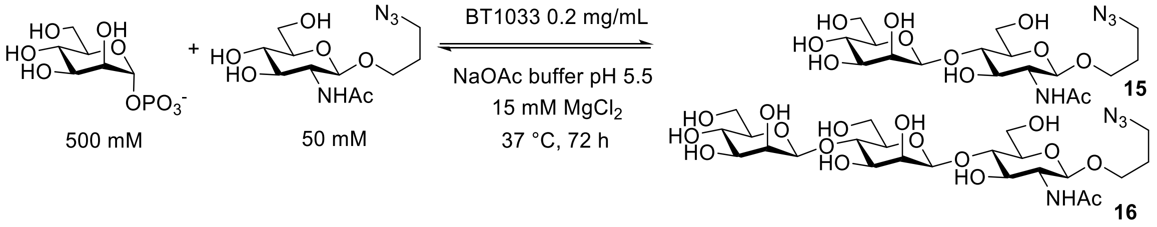

The reaction was assembled on a 500 µL scale containing 40 mM NaOAc pH 5.5 buffer, 500 mM mannose-1-phosphate (83 mg), 50 mM GlcNAc-N_3 (7.5 mg), 15 mM $MgCl_2$ and BT1033 (0.2 mg/mL). The reaction was incubated at 37 °C for 72 h. The reaction was stopped after the addition of 500 µL of EtOH to precipitate the enzyme. The sample was incubated at -20 °C for 12 h. The sample was then centrifuged at 4000 x g for 15 min at 4 °C and the supernatant transferred to a 50 mL falcon tube containing 40 mL of HPLC grade H_2O. The sample was then lyophilised and purified by BioGel P2 column chromatography in H_2O. Fractions containing the desired disaccharide (**15**, 0.014 mmol, 6.5 mg, 56%) and trisaccharide (**16**, 0.0038 mmol, 2.4 mg, 15%) respectively, were pooled and lyophilised.

Manβ1,4-GlcNAc-N_3 15

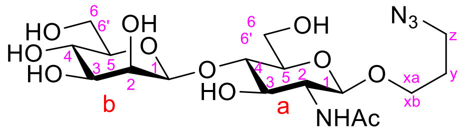

R_f = 0.74 (2:1:1, N-butanol/acetic acid/H_2O). ^{1}H NMR (700 MHz, D_2O) δ 4.78 (s, 1H, H1-b), 4.55 – 4.52 (m, 1H, H1-a), 4.08 (d, $J_{2,3}$ = 3.3 Hz, 1H, H2-b), 3.98 (dt, J = 10.9, 5.6 Hz, 1H, Hx-a), 3.95 – 3.91 (m, 2H, H6-a, H6-b), 3.79 – 3.72 (m, 5H, H6'-a, H6'-b, H5-a, H4-a, H2-a), 3.70 – 3.65 (m, 2H, H3-b, Hx-b), 3.61 – 3.55 (m, 2H, H4-b, H3-a), 3.43 (ddd, J = 9.8, 6.6, 2.3 Hz, 1H, H5-b), 3.41 – 3.35 (m, 2H, Hz), 2.06 (s, 3H, NHCOC<u>H</u>$_3$), 1.90 – 1.82 (m, 2H, Hy). ^{13}C (150 MHz, D_2O) δ 174.5 (NH<u>C</u>OCH$_3$), 101.1 (1C, J_{CH} = 162 Hz, C1-a), 100.0 (1C, J_{CH} = 160 Hz, C1-b), 78.9, 72.2 & 54.9 (C5-a, C4-a, C2-a), 76.4 (C5-b), 74.5 & 66.5 (C4-b & C3-a), 72.7 (1C, C3-b), 70.5 (C2-b), 67.1 (Cx), 60.8 & 60.1 (C6-a & C6-b), 47.7 (Cz), 28.0 (Cy), 22.1 (NHCO<u>C</u>H$_3$). HRMS (ESI) m/z calcd for $C_{17}H_{31}N_4O_{11}$ (M + H) 467.1984, found 467.1993. *IR* –3260 (C-H), 2095 (N=N=N), 1649 (C=O), 1559, 1373, 1056, 1028, 937, 800, 564.

Man$_2$β1,4-GlcNAc-N$_3$ 16

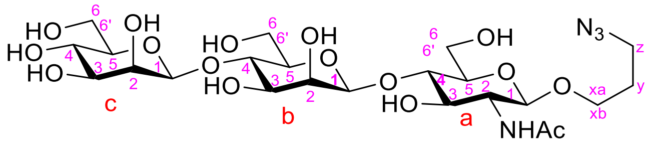

R_f = 0.65 (2:1:1, N-butanol/acetic acid/H_2O). ^1H (700 MHz, D_2O) δ 4.79 (1H, H1-c), 4.74 (s, 1H, H1-b), 4.56 – 4.52 (m, 1H, H1-a), 4.14 (d, *J* = 3.0 Hz, 1H, H2-c), 4.07 (d, *J* = 3.3 Hz, 1H, H2-b), 4.01 – 3.89 (m, 4H, Hx-a, H6'-b, H6'-a, H6'-c), 3.87 – 3.80 (m, 2H, H3-c, H4-a), 3.80 – 3.70 (m, 6H, H-2a, H6-b, H6-c, H6-a, X, X), 3.67 (m, 2H, Hx-b, H3-b), 3.56 (m, 3H, H3-a, H4-b, X), 3.45 (m, 1H, H5-b), 3.38 (m, 2H, Hz), 2.06 (s, 3H, NHCOC<u>H</u>$_3$), 1.90 – 1.78 (m, 2H, Hy). X= H4-c, H5-a or H5-c. ^{13}C DEPT (176 MHz, D_2O) δ 101.14 (C1-a), 100.18 (C1-b), 100.01 (C1-c), 78.85 (X), 76.51 (C5-b), 76.39 (C4-a), 74.99 (X), 74.53 (X), 72.72 (C3-b), 72.19 (X), 71.45 (C3-c), 70.45 (C2-b), 69.91 (C2-c), 67.14 (Cx), 66.66 (C4-b), 60.98 , 60.42 & 60.22 (C6-a, C6-b, C6-c), 55.00 (X), 47.75 (Cz), 28.08 (Cy), 22.12 (NHCO<u>C</u>H$_3$). X= C4-c, C2-a, C3-a, C5-c or C5-a. HRMS (ESI) m/z calcd for $C_{23}H_{40}N_4NaO_{16}$ (M + Na) 651.2332, found 651.2346. *IR* – 3302 (C-H), 2928, 2877, 2097 (N=N=N), 1974, 1649, 1421, 1375, 1066, 1031, 807

8.2 Synthesis of Manβ1,4-S-GlcNAc-N$_3$ 34, Man$_2$β1,4-S-GlcNAc-N$_3$ 35 and Man$_3$β1,4-S-GlcNAc-N$_3$ 36.

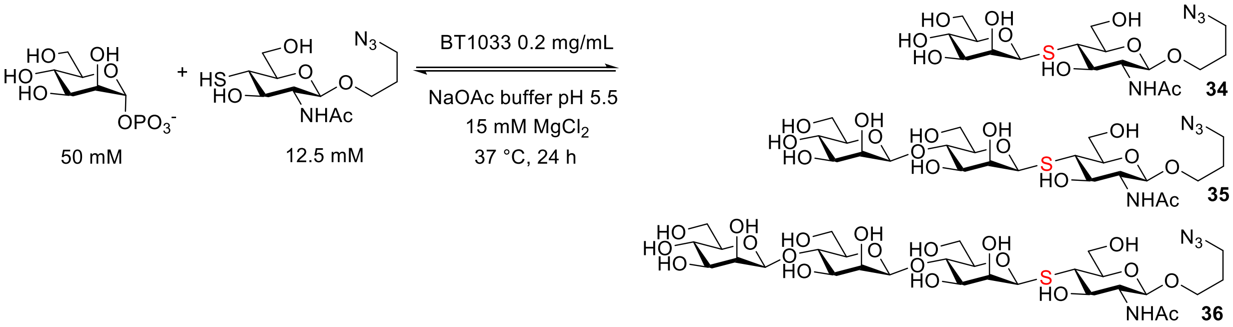

The reaction was assembled on a 3200 µL scale containing 40 mM NaOAc pH 5.5 buffer, 50 mM mannose-1-phosphate (53 mg), 12.5 mM GlcNAc-N$_3$ (12.8 mg), 15 mM $MgCl_2$, 5 mM TCEP (added from a 100 mM stock diluted in 40 mM NaOAc buffer adjusted to pH 5.5) and BT1033 (0.2 mg/mL). The reaction was incubated at 37 °C for 24 h. The reaction was stopped after the addition of 3200 µL of EtOH to precipitate the enzyme. The sample was incubated at -20 °C for 16 h. The sample was then centrifuged at 4000 x g for 20 min at 4 °C and the supernatant retained. The supernatant was diluted to 5 % (*v/v*) EtOH and lyophilised. The sample was then purified by BioGel P2 column chromatography in H_2O. Fractions containing the desired disaccharide (**34**, 0.0079mmol, 3.8 mg, 20%), trisaccharide (**35**, 0.0092 mmol, 5.9 mg, 23%) and tetrasaccharide (**36**, 0.0042, 3.4 mg, 11%) respectively were pooled and lyophilised.

Manβ1,4-S-GlcNAc-N_3 34

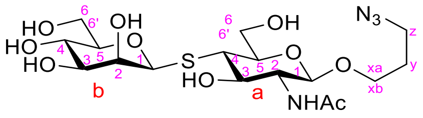

R_f = 0.76 (2:1:1, N-butanol/acetic acid/H_2O). ^{1}H (700 MHz, D_2O) δ 4.96 (s, 1H, H-1b), 4.51 (d, *J* = 8.2 Hz, 1H, H1-a), 4.15 (dd, *J* = 12.3, 1.9 Hz, 1H, H6-a), 4.07 (dd, *J* = 3.5 Hz, 1H, H2-b), 3.98 (dt, *J* = 10.4, 5.6 Hz, 1H, Hx-b), 3.94 (dd, *J* = 12.3, 5.3 Hz, 1H, H6'-a), 3.90 (dd, *J* = 12.4, 2.2 Hz, 1H, H6'b), 3.73 – 3.67 (m, 6H, H6-b, Hx-a, H3-b, H2-a, H5-a, H3-a), 3.59 (t, *J* = 9.8 Hz, 1H, H4-b), 3.43 (ddd, *J* = 10.0, 6.5, 2.3 Hz, 1H, H5-b), 3.39 (m, 2H, Hz), 2.90 – 2.87 (m, 1H, H4-a), 2.06 (s, 3H, NHAc), 1.86 (p, J_{CH2} = 6.4 Hz, CH_2, Hy). ^{13}C NMR (176 MHz, D_2O) δ 174.58(NH<u>CO</u>CH_3), 100.89 (C1-a), 82.13 (C1-b), 80.22 (C5-b), 76.19, 73.74, 71.05, 56.85 (C3-b, C2-a, C5-a, C3-a), 72.05 (C2-b), 67.02 (Cx), 66.53 (C4-b), 61.57 (C6-a), 61.11 (C6-b), 47.77 (Cz, C4-a), 28.08 (Cy), 22.15 (NHCO<u>C</u>H_3). HRMS (ESI) m/z calcd for $C_{17}H_{30}N_4NaO_{10}S$ (M + Na) 505.1575, found 505.1590. *IR* – 3288 (C-H), 2939, 2870, 2098 (N=N=N), 1973, 1644, 1560, 1375, 1120, 1030, 950, 616

Man_2β1,4-S-GlcNAc-N_3 35

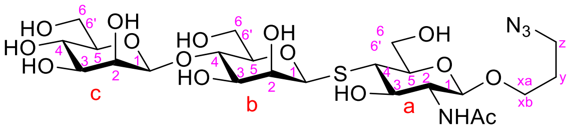

R_f = 0.69 (2:1:1, N-butanol/acetic acid/H_2O). ^{1}H (700 MHz, D_2O) δ 4.98 (s, 1H, H-1b), 4.75 (s, 1H, H1-c), 4.51 (d, *J* = 8.0 Hz, 1H, H1-a), 4.17 – 4.13 (m, 2H, H2-b & H-6a), 4.07 (dd, *J* = 3.2, 0.9 Hz, 1H, H2-c), 4.00 – 3.93 (m, 3H, Hx-b, H6'-a, H6'-b), 3.88 (dd, *J* = 12.4, 2.2 Hz, 1H, H6-c), 3.84 (m, 2H, H3-b, H5-c), 3.76 – 3.66 (m, 7H, H6-b, Hx-a, H2-a, H5-a, H3-a, H6'-c, H3-c), 3.59 – 3.54 (m, 2H, H4-b,H4-c), 3.45 (ddd, *J* = 9.5, 6.8, 2.2 Hz, 1H, H5-b), 3.39 (td, *J* = 6.5, 4.7 Hz, 2H, Hz), 2.89 (t, *J* = 10.3 Hz, 1H, H4-a), 2.07 (s, 3H, NHCOC<u>H_3</u>), 1.88 – 1.83 (m, 2H, Hy). ^{13}C NMR (176 MHz, D_2O) δ 174.54 (NH<u>C</u>OCH_3), 100.89 (C-1a), 100.14 (C1-c), 82.13 (C1-b), 78.74 (C4-b), 76.42 (C5-b), 76.37 (C3-b), 76.13, 72.75, 71.01 & 56.86 (C2-a, C5-a, C3-a, C3-c) 72.40 (C5-c), 71.59 (C2-b), 70.47 (C2-c), 67.02 (Cx), 66.66 (C4-c) 61.53 (C6-a), 60.98 (C6-b), 60.65 (C6-c), 47.83 (Cz), 47.77 (C4-a), 28.09 (Cy), 22.15 (NHCO<u>C</u>H_3). HRMS (ESI) m/z calcd for $C_{23}H_{41}N_4O_{15}S$ (M + H) 645.2284, found 645.2287. *IR* – 3337, 2947, 2870, 2097.5 (N=N=N), 1973, 1653, 1564, 1379, 1309, 1093, 1034, 795

Man$_3$β1,4-S-GlcNAc-N$_3$ 36

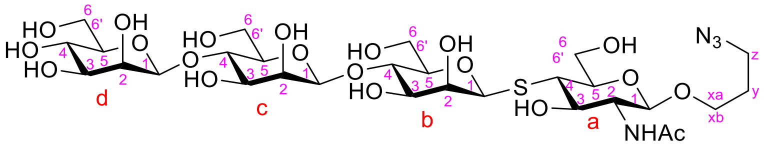

R$_f$ = 0.66 (2:1:1, N-butanol/acetic acid/H_2O). ^{1}H (700 MHz, D_2O) δ 4.98 (s, 1H, H1-b), 4.77 (s, 1H, H1-d), 4.74 (s, 1H, H1-c), 4.51 (d, *J* = 7.9 Hz, 1H, H1-a), 4.14 (m, 3H, H2-b, H2-d, H6-a), 4.07 (dd, *J* = 3.3, 0.9 Hz, 1H, H2-c), 3.99 – 3.97 (m, 1H, Hx-a), 3.96 – 3.91 (m, 3H, H6'-a, H6'-b, H6'-c/d), 3.88 (dd, *J* = 12.5, 2.2 Hz, 1H, H6'-c/d), 3.85 – 3.82 (m, 4H, H3-b, H3-d, H3-a, H5-c/H5-d), 3.78 – 3.65 (m, 8H, H6-b, H6-c, H6-d, Hx-b, H2-a, H3-c, H5-a, H4-c), 3.60 – 3.54 (m, 3H, H4-b, H4-d, H5-c/H5-d), 3.47 – 3.44 (m, 1H, H5-b), 3.39 (m, 2H, Hz), 2.89 (t, *J* = 10.3 Hz, 1H, H4-a), 2.07 (s, 3H, $NHCO\underline{C}H_3$), 1.89 – 1.79 (m, 2H, Hy). ^{13}C NMR (176 MHz, D_2O) δ 174.54 ($NH\underline{C}OCH_3$), 100.89 (C1-a), 100.18 (C1-c), 100.08 (C1-d) 82.13 (C1-b), 78.75 (C4-b), 76.61, 72.38 & 71.45 (C3-b, C3-d, C5-c/C5-d), 76.40 (C5-b), 76.13 (C3-a), 76.29 (C4-c), 72.73 & 56.83, (C2-a, C3-c), 75.02 & 66.66 (C4-d, C5-c/C5-d) 71.62 (C2-b), 71.00 (C5-a), 70.46 (C2-c), 69.89 (C2-d), 67.02 (Cx), 61.52 (C6-a), 60.98 & 60.51 (C6-b & C6-x), 60.63 (C6-x), 47.77 (Cz), 47.81 (C4-a), 28.09 (Cy), 22.16 ($NHCO\underline{C}H_3$). HRMS (ESI) m/z calcd for $C_{29}H_{50}N_4NaO_{20}S$ (M + Na) 829.2631, found 829.2640. *IR* – 3281, 2932, 2877, 2098 (N=N=N), 1977, 1643, 1564, 1374, 1070, 1026, 811

8.3 Synthesis of 6Cl-Manβ1,4-GlcNAc-N$_3$ 18

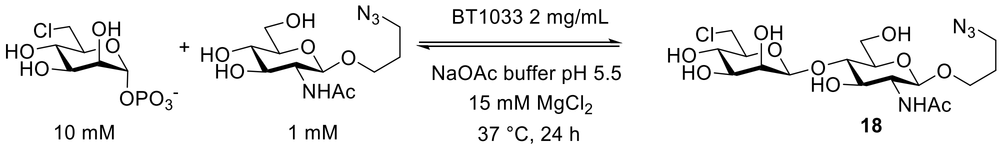

The reaction was assembled on an 8000 µL scale containing 40 mM NaOAc pH 5.5 buffer, 10 mM mannose-1-phosphate (22.2 mg), 1 mM GlcNAc-N$_3$ (2.4 mg), 15 mM $MgCl_2$ and BT1033 (2 mg/mL). The reaction was incubated at 37 °C for 24 h. The reaction was stopped after the addition of 8000 µL of EtOH to precipitate the enzyme. The sample was incubated at -20 °C for 30 min. The sample was then centrifuged at 4000 x g for 15 min at 4 °C and the supernatant retained. The pellet was further washed with 8000 µL of 50 % (*v/v*) EtOH in H_2O, centrifuged (4000 x g, 15 min, 4 °C) and the supernatant added to the previously retained supernatant. The sample was diluted to 5 % (*v/v*) EtOH and lyophilised. The sample was then purified by BioGel P2 column chromatography in H_2O. Fractions containing the desired product (**18**, 0.0054, 2.6 mg, 68%) were pooled and lyophilised.

6Cl-Manβ1,4-GlcNAc-N_3 18

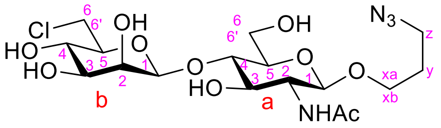

R_f = 0.81 (2:1:1, N-butanol/acetic acid/H_2O). ^1H (700 MHz, D_2O) δ 4.84 (s, 1H, H1-b), 4.54 (d, *J* = 8.0 Hz, 1H, H1-a), 4.09 (s, 1H, H-2b), 4.01 – 3.96 (m, 2H, Hx-a, H6'b), 3.91 (dd, *J* = 12.4, 2.2 Hz, 1H, H6'-a), 3.83 – 3.71 (m, 5H, H2-a, H6-a, H6-b, H4-a, H4-b), 3.71 – 3.66 (m, 3H, H3-b, Hx-b, H3-a), 3.64 (ddd, *J* = 9.6, 6.0, 2.4 Hz, 1H, H5-b), 3.60 (ddd, *J* = 9.6, 5.1, 2.2 Hz, 1H, H5-a), 3.42 – 3.35 (m, 2H, Hz), 2.06 (s, 3H, NHCOC<u>H</u><u>3</u>), 1.88 – 1.83 (m, 2H, Hy). ^{13}C NMR (176 MHz, D_2O) δ 174.49 (NH<u>C</u>OCH3), 101.10 (C1-a), 100.30 (C1-b), 79.62 (C4-a), 75.28 (C5-b), 74.40 (C5-a), 72.37 (C3-a), 72.18 (C4-b) 70.40 (C2-b), 67.30 (C3-b), 67.16 (Cx), 60.18 (C6-a), 54.95 (C2-a), 47.76 (Cz), 43.91 (C6-b), 28.09 (Cy), 22.15 (NHCO<u>C</u>H3). HRMS (ESI) m/z calcd for $C_{17}H_{29}ClN_4NaO_{10}$ (M + Na) 507.1464, found) 507.1476. *IR* – 3287, 2932, 2874, 2097, 1657, 1556, 1379, 1305, 1060, 803

7.4 Synthesis of Manβ1,4-6F-GlcNAc-N_3 25, Man_2β1,4-6F-GlcNAc-N_3 26, Man_3β1,4-6F-GlcNAc-N_3 27 and Man_4β1,4-6F-GlcNAc-N_3 37

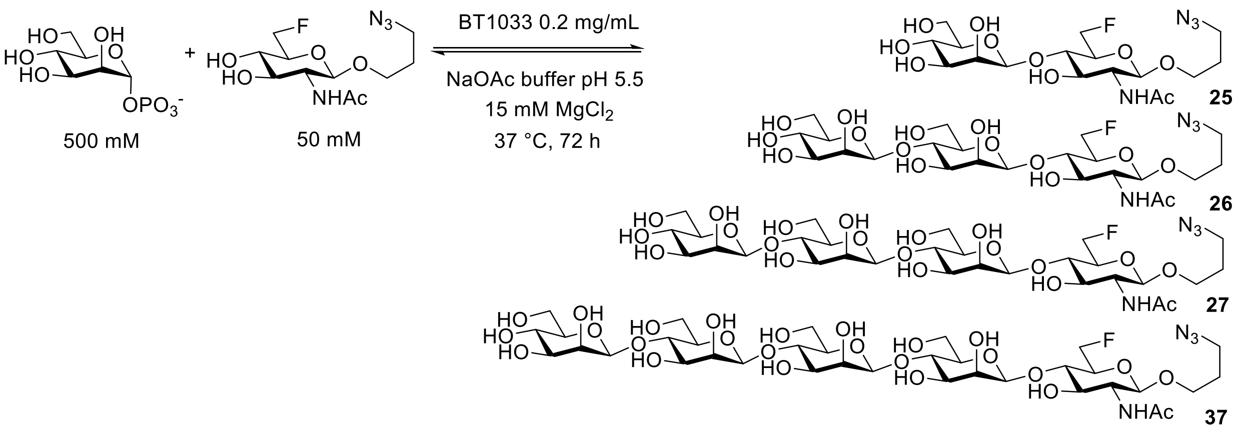

The reaction was assembled on a 500 µL scale containing 40 mM NaOAc pH 5.5 buffer, 500 mM mannose-1-phosphate (83 mg), 50 mM 6F-GlcNAc-N_3 (7.5 mg), 15 mM $MgCl_2$ and BT1033 (0.2 mg/mL). The reaction was incubated at 37 °C for 72 h. The reaction was stopped after the addition of 500 µL of EtOH to precipitate the enzyme. The sample was incubated at -20 °C for 12 h. The sample was then centrifuged at 4000 x g for 15 min at 4 °C and the supernatant transferred to a 50 mL falcon tube containing 40 mL of HPLC grade H_2O. The sample was then lyophilised and purified by BioGel P2 column chromatography in H_2O. Fractions containing the desired disaccharide (**25**, 0.0029, 1.4 mg,

12%), trisaccharide (**26**, 0.0033, 2.1 mg, 13%), tetrasaccharide (**27**, 0.0017, 1.4 mg, 7%) and pentasaccharide (**37**, 0.0014, 1.3 mg, 5%) respectively, were pooled and lyophilised.

Manβ1,4-6F-GlcNAc-N_3 25

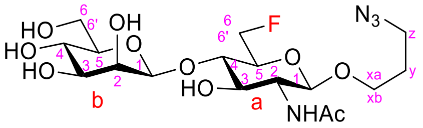

R_f = 0.81 (2:1:1, N-butanol/acetic acid/H_2O). ^{1}H (700 MHz, D_2O) δ 4.79 (1H, H-1b), 4.77 (1H, H6-a), 4.71 (s, 1H, H6’-a), 4.59 (d, J = 7.9 Hz, H1-a), 4 .09 (d, J = 3.3 Hz, 1H, H2-b), 3.97 (td, J = 10.7 Hz, J = 6.0, 1H, Hx-a), 3.94 (dd, J = 12.4 Hz, J = 2.3 Hz, 1H, H6-b), 3.85 – 3.66 (m, 7H, H1-b, Hx-b, H3-b, H6’-b, H3-a, H4-a, H5-a), 3.61 – 3.57 (m, 1H, H4-b), 3.46 – 3.42 (m, 1H, H5-b), 3.38 (m, 2H, Hz), 2.06 (s, 3H, NHCOC$\underline{H_3}$), 1.85 (m, 2H, Hy). ^{13}C (175 MHz, D_2O) δ 174.55 (C=O), 101.3 (J_{CH} = 159 Hz, C-1a), 100.0 (J_{CH} = 164 Hz, C-1b), 81.7 (J_{CH} = 168.4 Hz, C6-a), 77.6, 76.4, 72.8, 72.0 & 55.1 (C1-b, C3-b, C3-a, C4-a, C5-a), 70.7 (C2-b), 67.6 (Cx), 66.5 (C4-b), 60.9 (C6-b), 47.8 (Cz), 28.2 (Cy), 22.1 (NHCO$\underline{C}H_3$). ^{19}F (400 MHz, D_2O) δ -234.04 (td, J = 47.2, 28.3 Hz). HRMS (ESI) m/z calcd for $C_{17}H_{29}FN_4NaO_{10}$ (M + Na) 491.1760, found 491.1761

Man_2β1,4-6F-GlcNAc-N_3 26

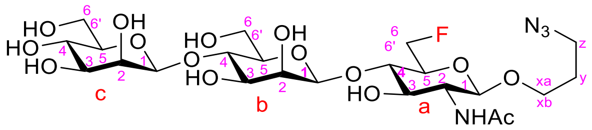

R_f = 0.71 (2:1:1, N-butanol/acetic acid/H_2O). ^{1}H (700 MHz, D_2O) δ 4.80 (s, 1H, H1-c) 4.74 (s, 1H, H-1b), 4.79 & 4.71 (s, 1H, H6-a & H6’-a) 4.59 (d, J = 7.7 Hz, 1H, H1-a), 4.15 (d, J = 2.9 Hz, 1H, H2-c), 4.15 (d, J = 2.9 Hz, 1H, H2-b), 4.00 – 3.89 (m, 3H, Hx-a, H6’-b, H6’-c), 3.88 – 3.81 (m, 3H, H3-c, H3-a, H4-a), 3.80 – 3.65 (m, 7H, Hxb, H6-b, H6-c, H2-a,H3-b, H5-a, H5-b/c), 3.60 – 3.54 (m, 2H, H4-b, H4-c), 3.45 (ddd, J = 9.5, 6.8, 2.2 Hz, 1H, H5-b/c), 3.39 (m, 2H, Hz), 2.07 – 2.05 (m, 3H, NHCOC$\underline{H_3}$) , 1.88 – 1.83 (m, 2H, Hy). ^{13}C NMR (176 MHz, D_2O) δ 174.51 (C=O), 101.26 (C1-a), 100.18 (C1-b), 99.89 (C1-c), 81.54 (C6-a, J = 167.2 Hz), 77.58 & 71.41 (C3-c, C3-a), 76.49 (C4-a), 76.40 (C5-b/c), 75.01 (C4-c), 73.00, 72.72, 72.01, 54.97 (C2-a, C3-b, C5-a, C5-b/c),70.45 (C2-b), 69.90 (C2-c), 67.27 (Cx), 66.66 (C4-b), 60.98 & 60.42 (C6-b & C6-c), 47.73 (Cz), 28.09 (Cy), 22.13 (NHCO$\underline{C}H_3$). ^{19}F (400 MHz, D_2O) δ -234.01 (td, J = 47.3, 28.7 Hz). HRMS (ESI) m/z calcd for $C_{23}H_{39}FN_4NaO_{15}$ (M + Na) 653.2288, found 653.2292. *IR* – 3329, 2947, 2858, 2101 (N=N=N), 1657, 1568, 1433, 1379, 1060

Man$_3$β1,4-6F-GlcNAc-N$_3$ 27

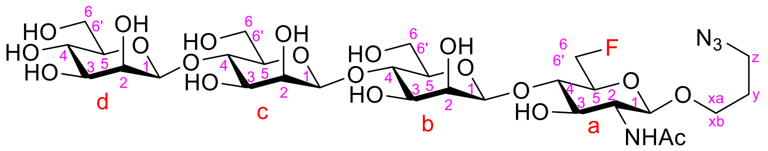

R_f = 0.62 (2:1:1, N-butanol/acetic acid/H_2O). ^1H (700 MHz, D_2O) δ 4.81 (s, 1H, H1-d), 4.77 (s, 1H, H6-a), 4.76 (s, 1H, H1-c) 4.75 – 4.74 (s, 1H, H1-b), 4.71 (s, 1H, H6'-a), 4.59 (d, *J* = 7.8 Hz, 1H, H1-a), 4.15 (d, *J* = 3.3 Hz, 1H, H2-d), 4.14 (d, *J* = 2.5 Hz, 1H, H2-c), 4.07 (d, *J* = 3.6 Hz, 1H, H2-b), 4.00 – 3.89 (m, 4H, Hx-a, H6-b, H6-c, H6-d), 3.88 – 3.64 (m, 14H, H2-a, H3-b, H3-c, H3-d, H5-a, Hx-b, H6'-b, H6'-c, H6'-d, H4-c/H4-d, H5-c, H5-d, H4-a, H5-d), 3.60 – 3.54 (m, 3H, H3-a, H4-b, H4-c/H4-d), 3.45 (ddd, *J* = 9.5, 6.8, 2.3 Hz, 1H, H5-b), 3.38 (m, 2H, Hz), 2.06 (s, 3H, NHCOC$\underline{H_3}$), 1.90 – 1.81 (m, 2H, Hy). ^{13}C NMR (176 MHz, D_2O) δ 174.51 (C=O), 101.26 (C1-a), 100.18 & 100.12 (C1-b, C-1c), 99.89 (C1-d) 81.58 (C6-a, *J* = 168.3 Hz), 77.58, 76.61, 76.40, 73.05, 72.96, 72.73, 72.00, 71.42, 71.38 & 54.98 (C2-a, C3-b, C3-c, C3-d, C5-a, C4-c/C4-d, C5-c, C4-a, C5-d), 75.01 & 66.65 (C3-a, C4-b, C4-c/C4-d), , 70.45 (C2-b), 69.93 & 69.88 (C2-c & C2-d), 67.27 (Cx), 60.97, 60.51 & 60.39 (C6-b, C6-c, C6-d), 47.73 (Cz), 28.09 (Cy), 22.14 (NHCO$\underline{C}H_3$). ^{19}F NMR (376 MHz, D_2O) δ -234.02 (td, *J* = 47.2, 28.5 Hz). HRMS (ESI) m/z calcd for $C_{29}H_{49}FN_4NaO_{20}$ (M + Na) 815.2816, found 815.2824. *IR* – 3314, 2943, 2874, 2101, 1657, 1564, 1305, 1062, 1030, 807

Man$_4$β1,4-6F-GlcNAc-N$_3$ 37

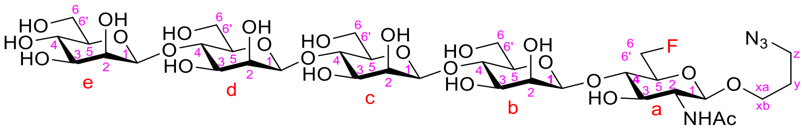

R_f = 0.48 (2:1:1, N-butanol/acetic acid/H_2O). ^1H (700 MHz, D_2O) δ 4.83 – 4.72 (5H, H1-e, H1-d, H1-c, H1-b, H6'-a), 4.71 (s, 1H, H6-a), 4.59 (d, *J* = 7.8 Hz, 1H, H1-a), 4.14 (m, 3H, H2-c, H2-d, H2-e), 4.07 (d, *J* = 3.7 Hz, 1H, H2-b), 3.99 – 3.89 (m, 5H, Hx-a, H6'-b, H6'-c, H6'-d, H6'-e), 3.88 – 3.64 (m, 16H, H2-a, H3-b, Hx-b, H6-b, H6-c, H6-d, H6-e, H3-c, H3-d, H3-e, X, X, X, X, X, X), 3.60 – 3.54 (m, 4H, H4-b, X, X, X), 3.45 (ddd, *J* = 9.5, 6.8, 2.3 Hz, 1H, H5-b), 3.38 (m, 2H, Hz), 2.06 (s, 3H, NHCOC$\underline{H_3}$), 1.90 – 1.82 (m, 2H, Hy). X= H3-a, H4-a, H4-c, H4-d, H4-e, H5-a, H5-c, H5-d or H5-e. DEPT ^{13}C NMR (176 MHz, D_2O) δ 101.26 (C1-a), 100.18, 100.12 & 99.90 (C1-e, C1-d, C1-c, C1-b), 81.62 (C6-a), 77.56, 76.60, 76.42, 76.39,75.01, 73.06, 72.95, 72.73, 72.00, 71.43, 71.39, 66.65, 54.97 (C2-a, C3-b, C3-c, C3-d, C3-e, C4-b, C3-a, C4-a, C4-c, C4-d, C4-e, C5-a, C5-c, C5-d, C5-e), 76.53 (C5-b), 70.45 (C2-b), 69.92 & 69.87 (C2-e, C2-d, C2-c), 67.27 (Cx), 60.95, 60.91, 60.50 & 60.40 (C6-b, C6-c, C6-d, C6-e), 47.73 (Cz), 28.08 (Cy), 22.22 (NHCO$\underline{C}H_3$). ^{19}F NMR (376 MHz, D_2O) δ -234.05 (td, J = 47.3, 28.8 Hz). HRMS (ESI) m/z calcd for

$C_{35}H_{59}FN_4NaO_{25}$ (M + Na) 977.3345, found 977.3308. *IR* – 3312, 2946, 2888, 2101, 1650, 1375, 1066, 1032, 810.

9. NMR Spectra

Manβ1,4-GlcNAc-N_3 15

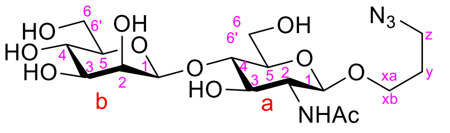

^{1}H NMR

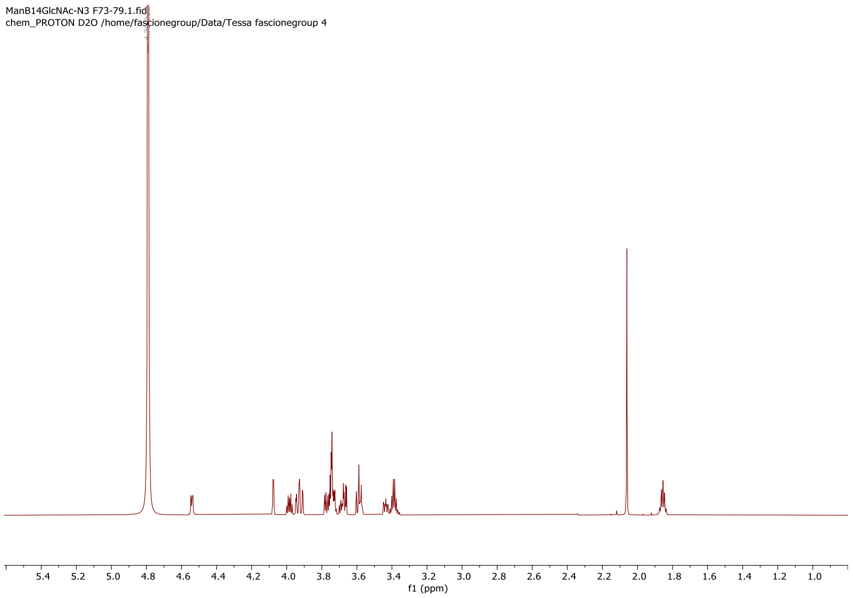

^{13}C NMR

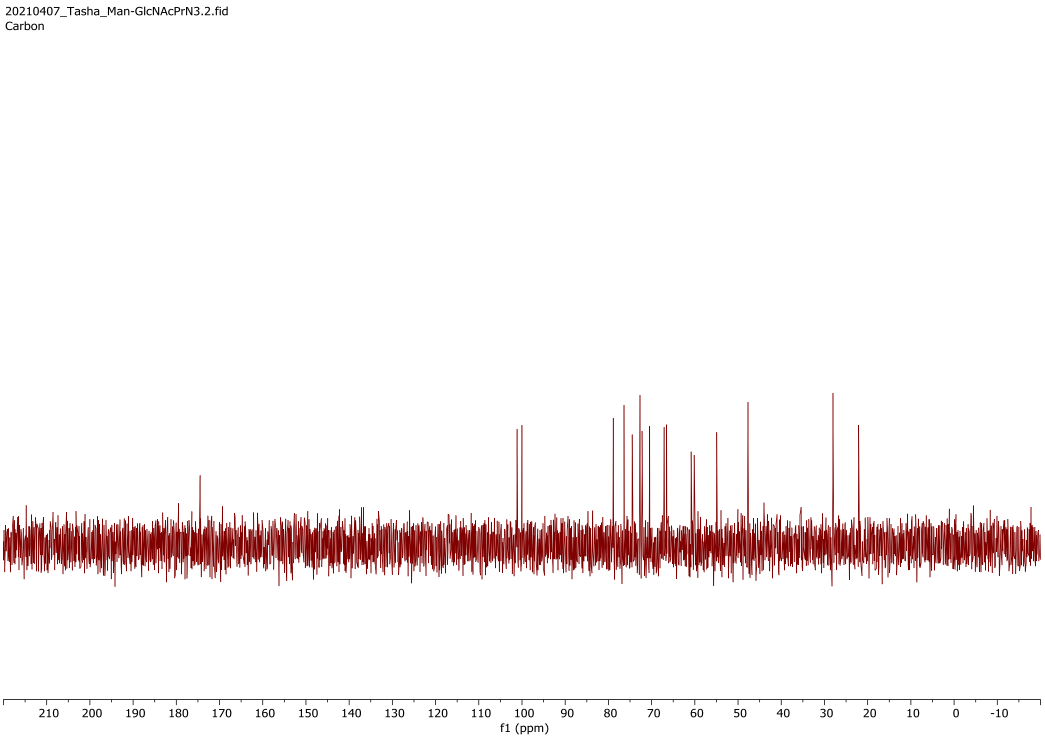

IPAP HSQC (^{13}C HSQC with multiplicity editing)

Spectrum 1

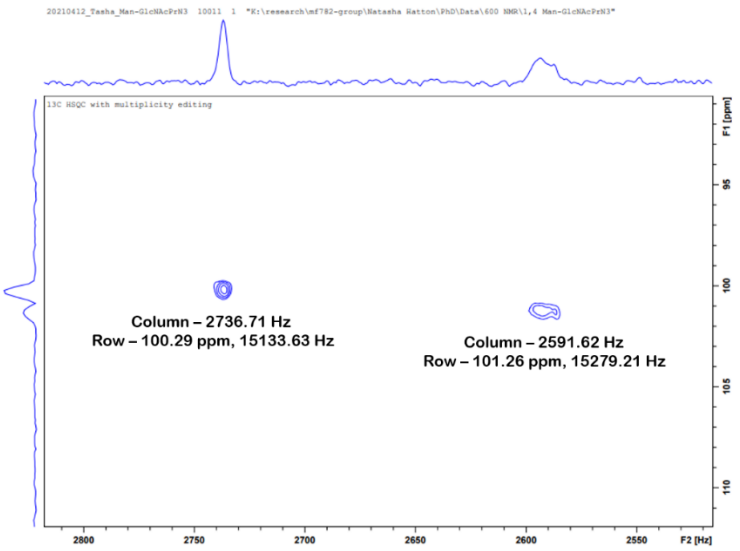

Spectrum two

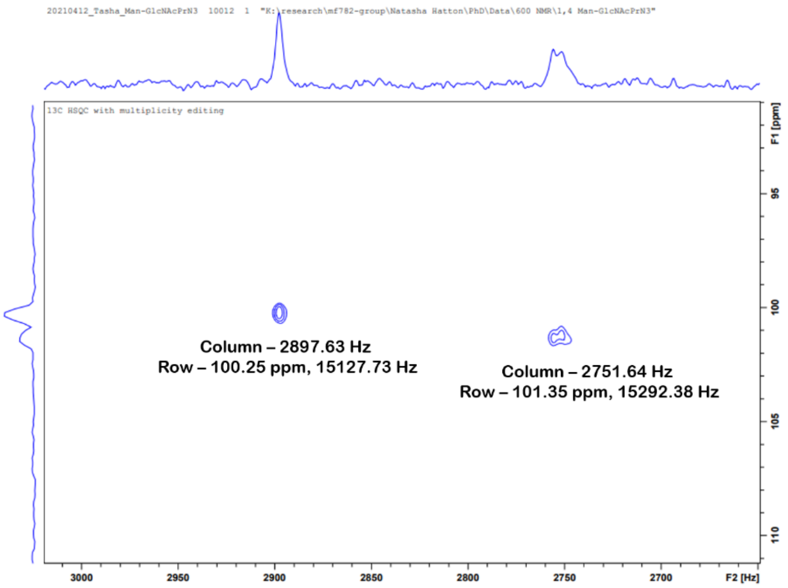

Table S3. Results of the two IPAP HSQC (13C HSQC with multiplicity editing) spectra for the two anomeric carbons and analysis of the results to give the $^{1}J_{CH}$ coupling constants.

Carbon	Spectrum one	Spectrum two	$^{1}J_{CH}$ coupling constant (Hz)
C-1 mannose	2736.71	2897.63	160.92
C-1 Glucosamine	2591.62	2751.64	160.02

Man₂β1,4-GlcNAc-N₃ 16

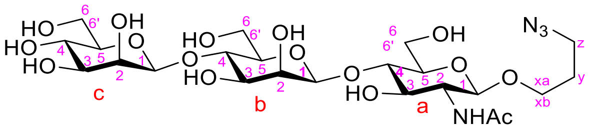

^{1}H

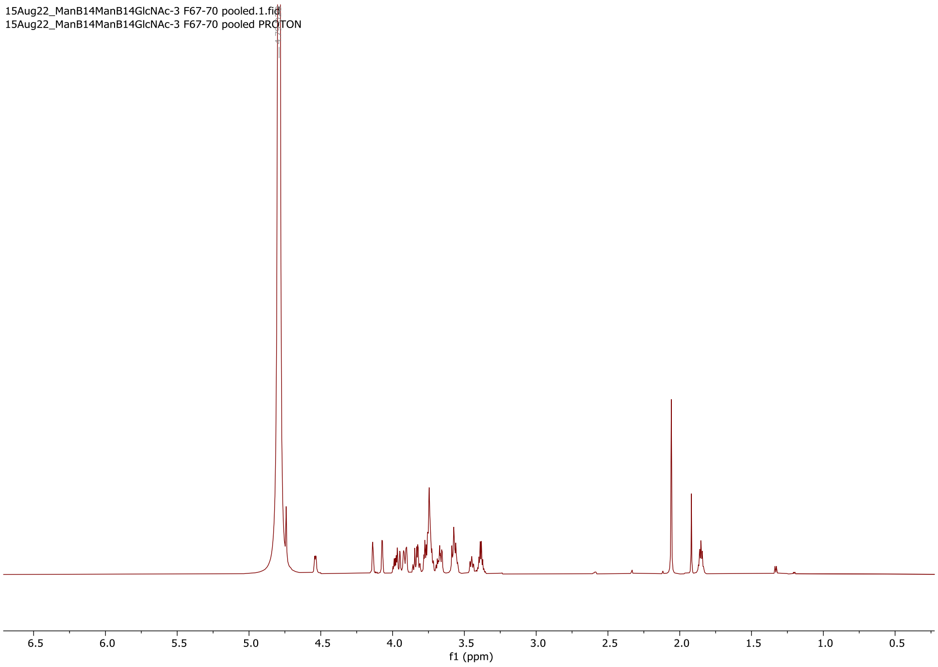

DEPT ^{13}C

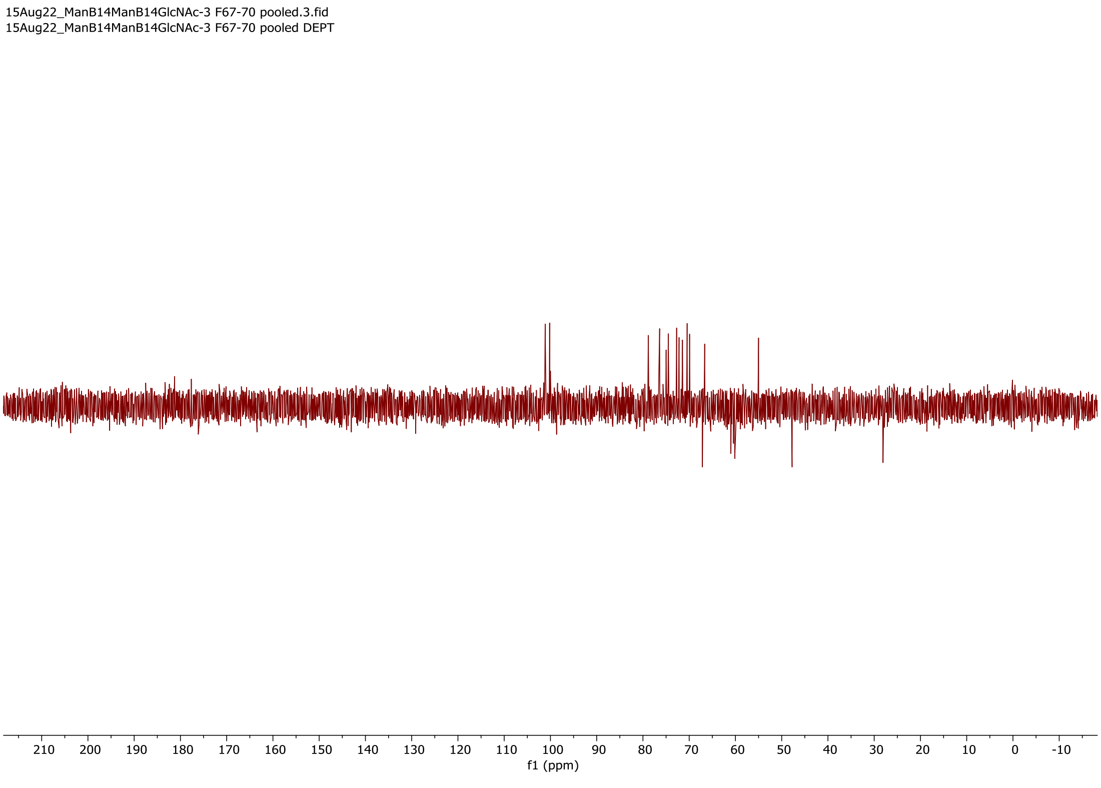

HSQC with anomeric proton and carbon cross couplings shown.

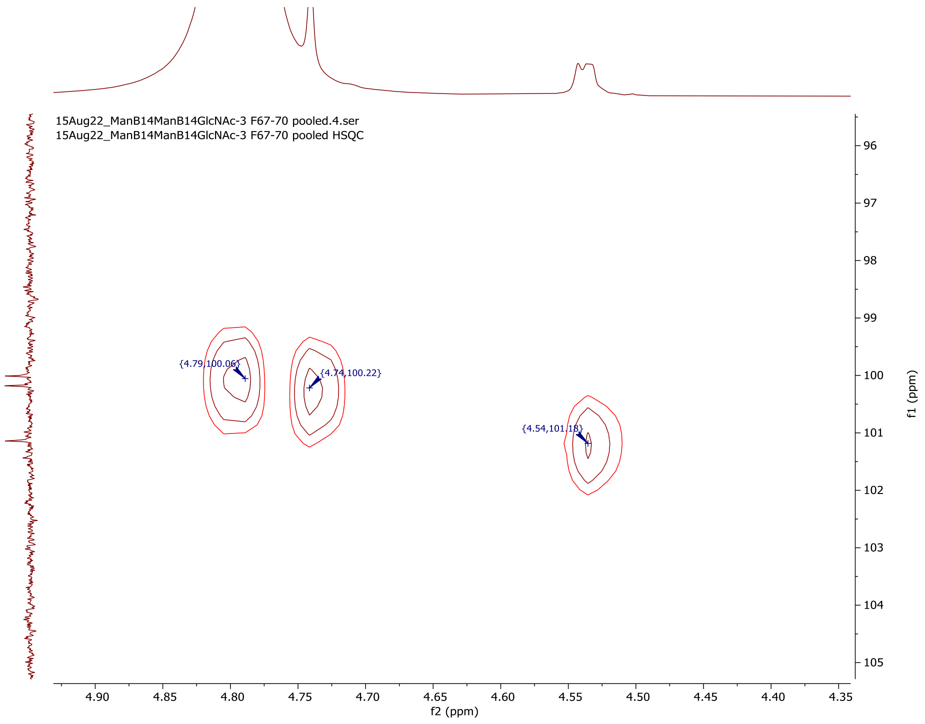

6Cl-Manβ1,4-GlcNAc-N_3 18

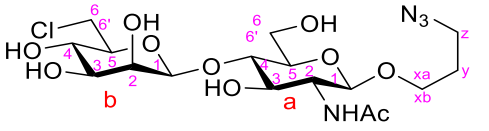

^{1}H NMR

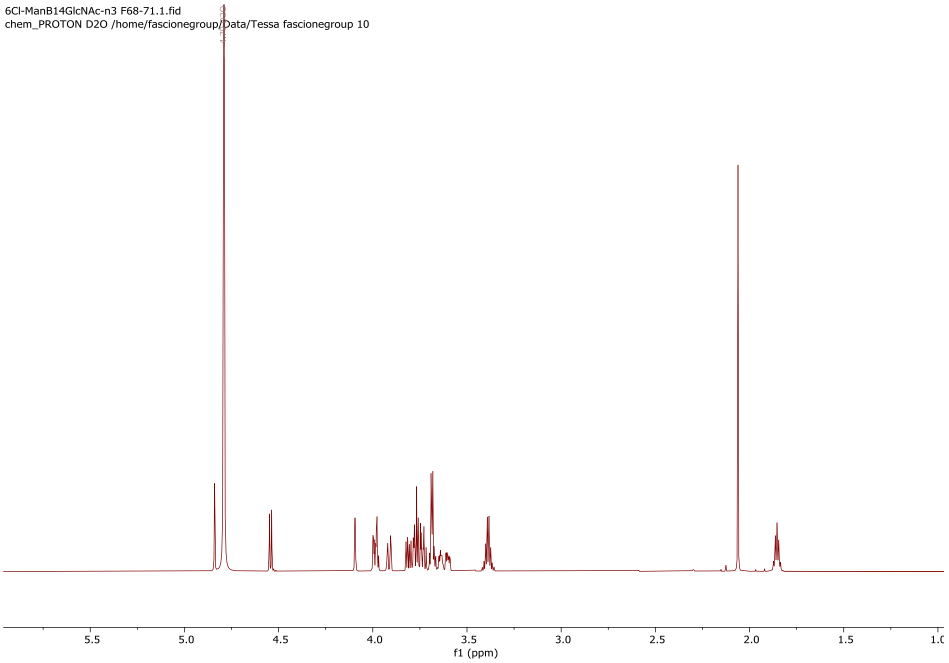

^{13}C

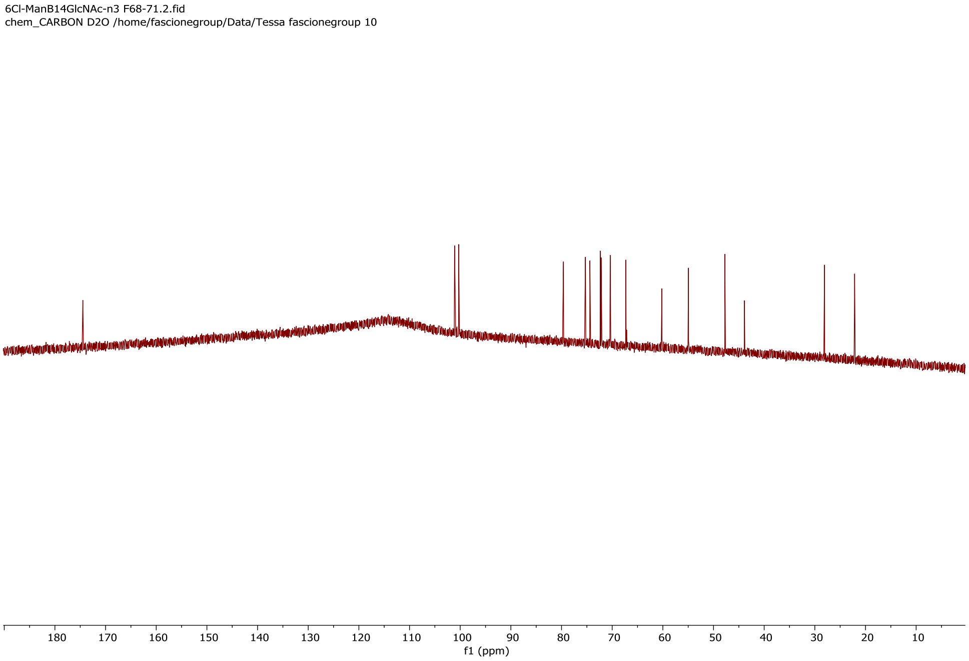

HMBC

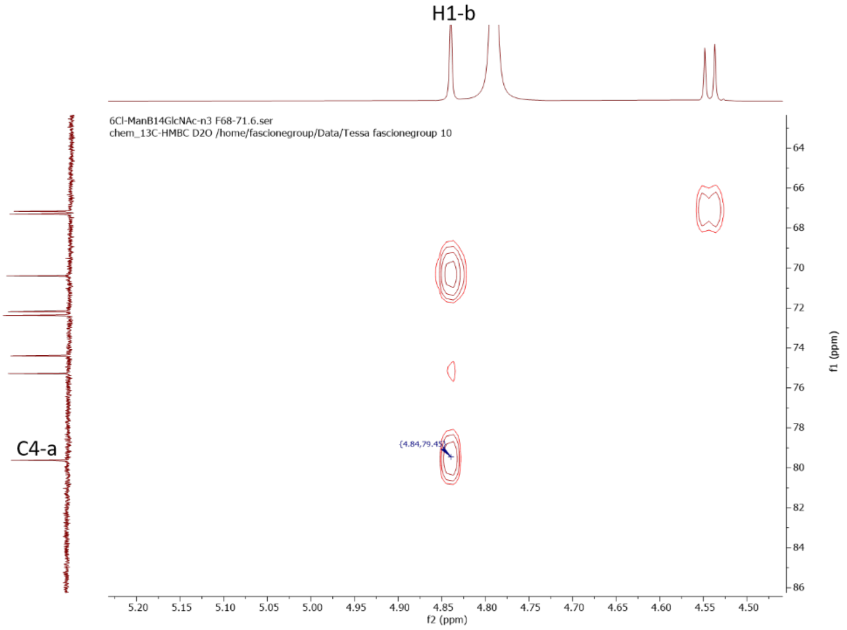

Manβ1,4-S-GlcNAc-N_3 34

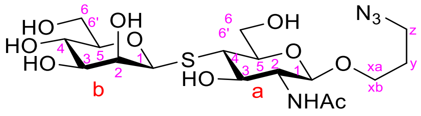

^1H

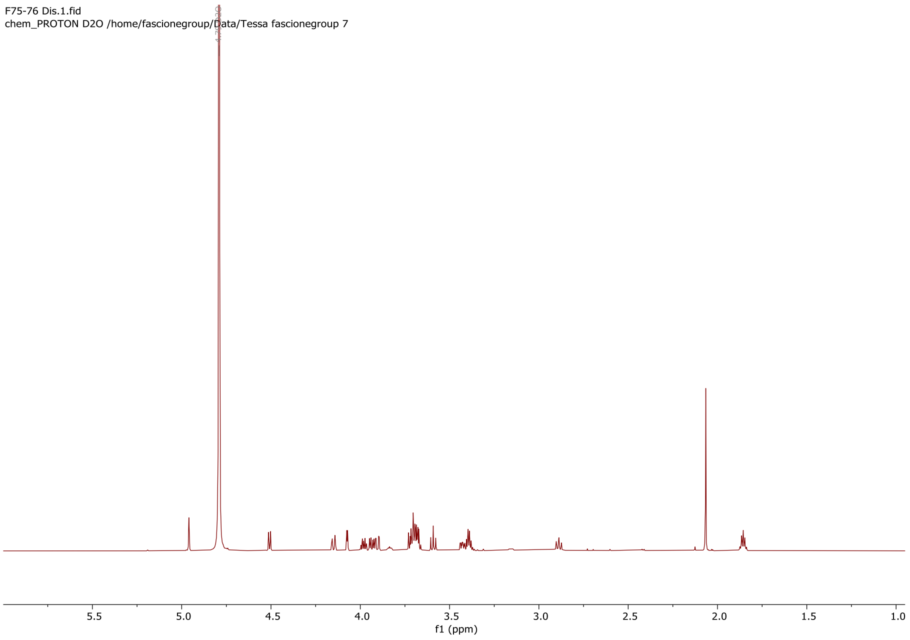

^{13}C

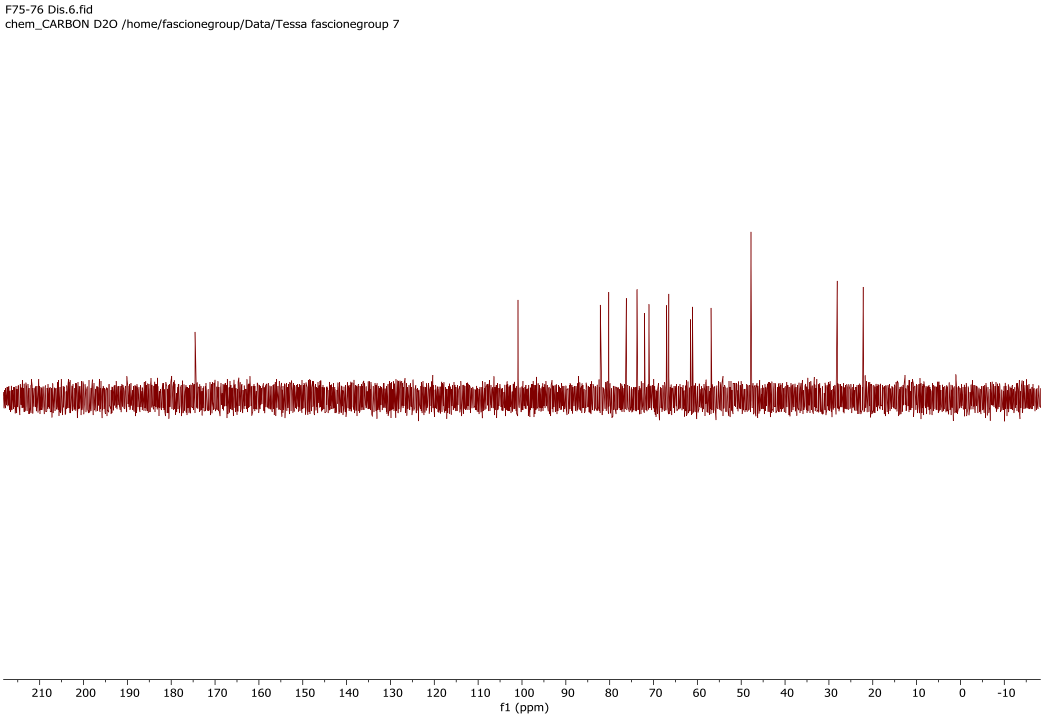
F75-76 Dis.6.fid
chem_CARBON D2O /home/fascionegroup/Data/Tessa fascionegroup 7
210 200 190 180 170 160 150 140 130 120 110 100 90 80 70 60 50 40 30 20 10 0 -10
f1 (ppm)

HSQC

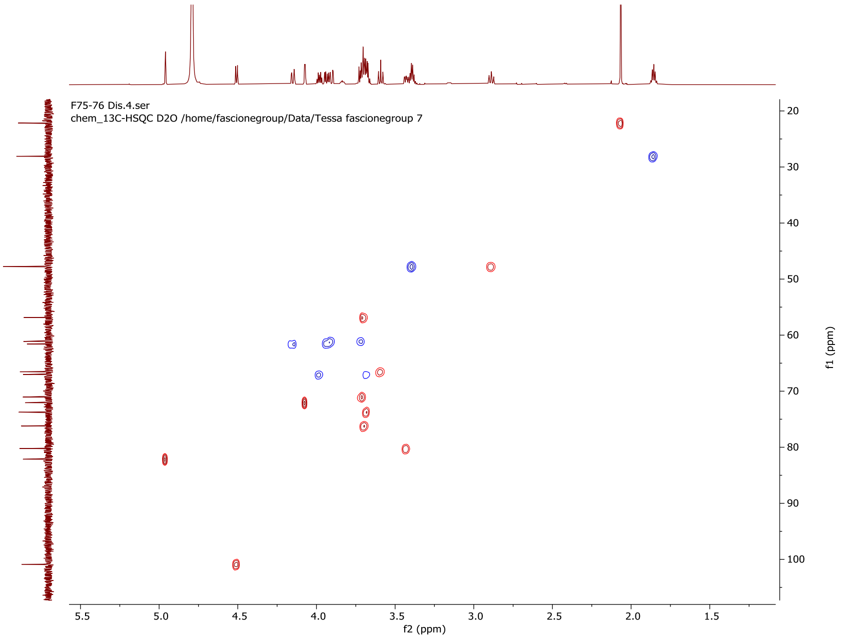
F75-76 Dis.4.ser
chem_13C-HSQC D2O /home/fascionegroup/Data/Tessa fascionegroup 7
5.5 5.0 4.5 4.0 3.5 3.0 2.5 2.0 1.5
f2 (ppm)
20 30 40 50 60 70 80 90 100
f1 (ppm)

HMBC

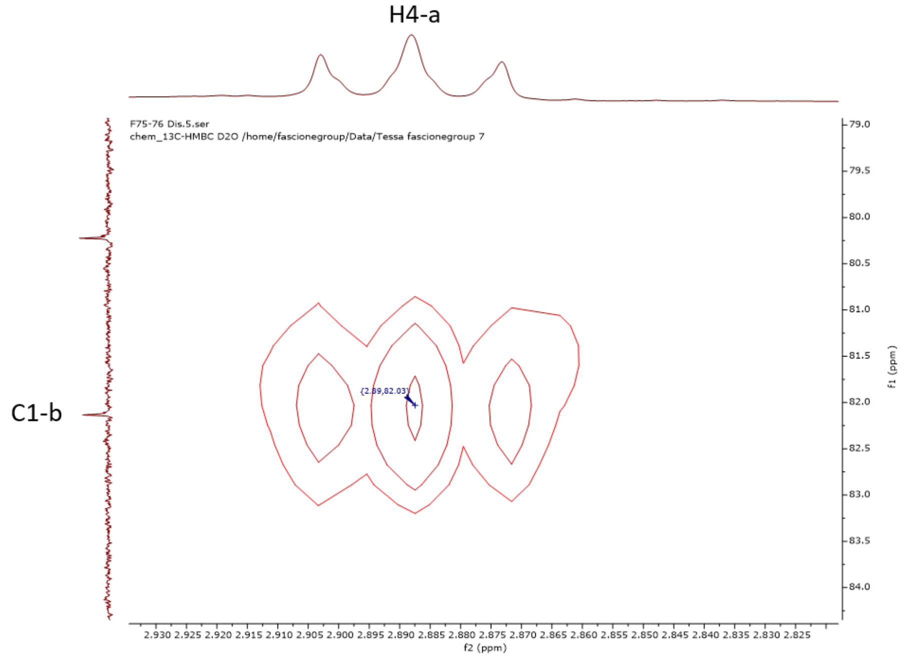

Man$_2$β1,4-S-GlcNAc-N$_3$ 35

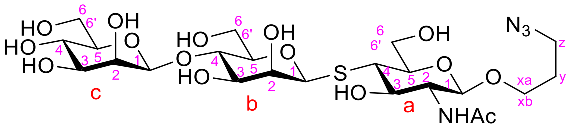

^{1}H

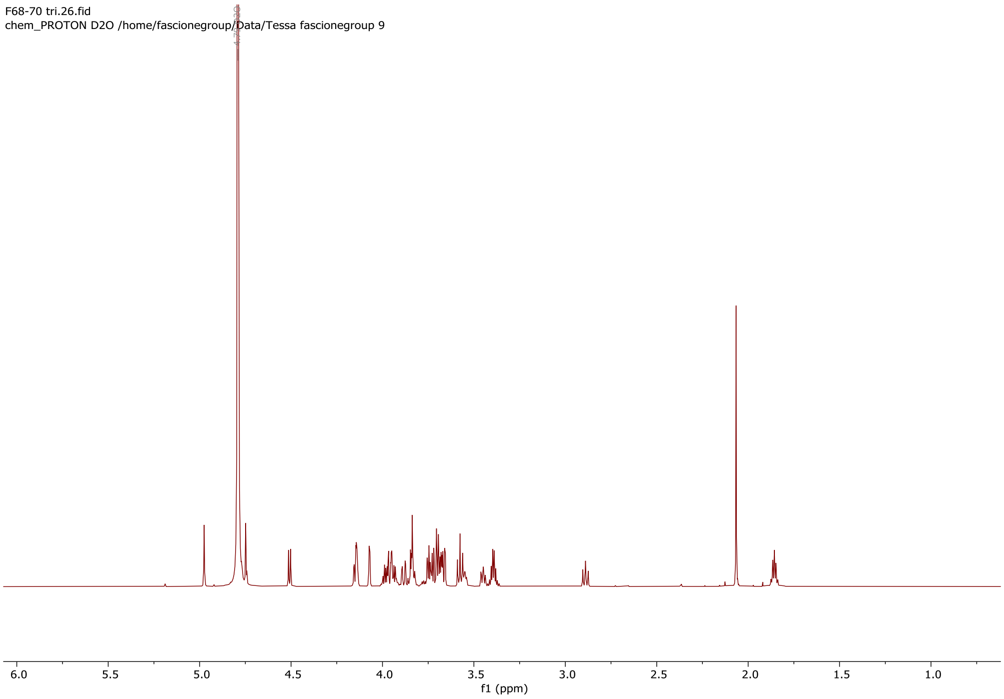

^{13}C

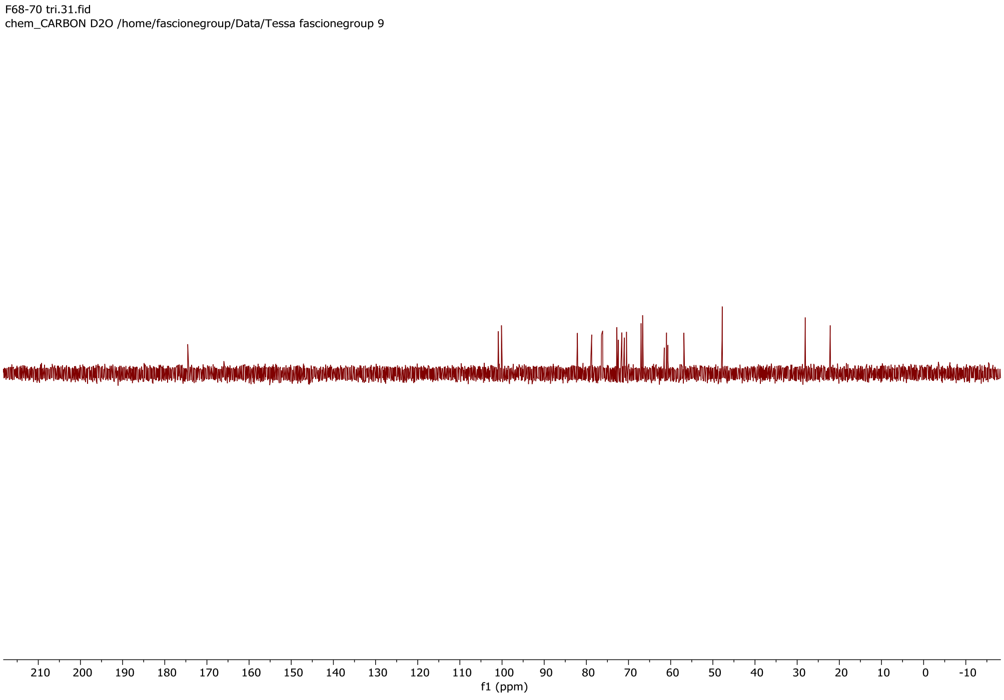

F68-70 tri.31.fid
chem_CARBON D2O /home/fascionegroup/Data/Tessa fascionegroup 9
210 200 190 180 170 160 150 140 130 120 110 100 90 80 70 60 50 40 30 20 10 0 -10
f1 (ppm)

HSQC

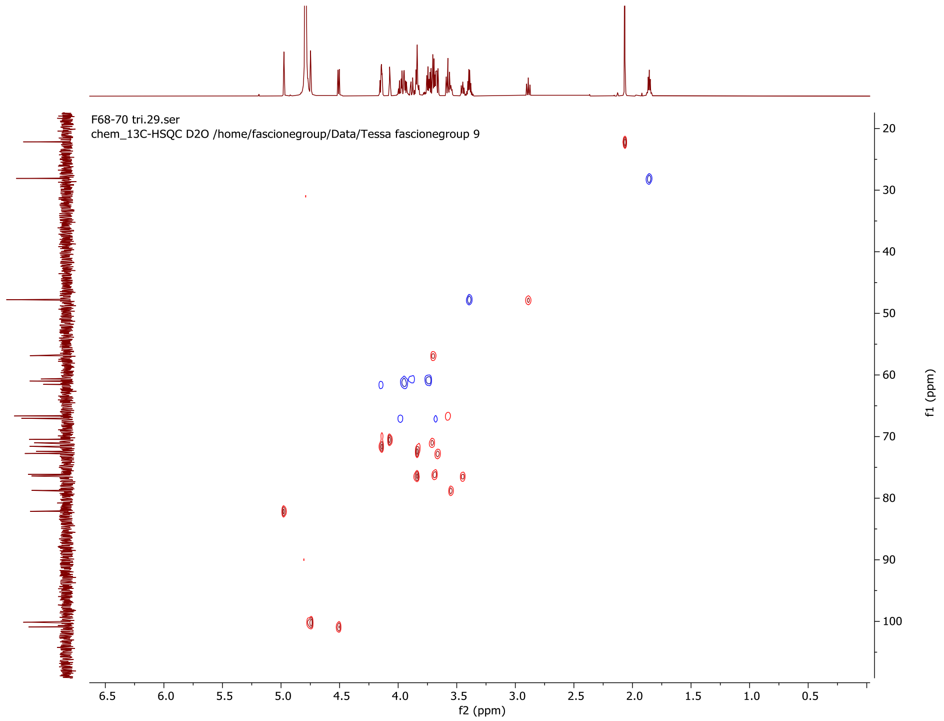

F68-70 tri.29.ser
chem_13C-HSQC D2O /home/fascionegroup/Data/Tessa fascionegroup 9
20 30 40 50 60 70 80 90 100
f1 (ppm)
6.5 6.0 5.5 5.0 4.5 4.0 3.5 3.0 2.5 2.0 1.5 1.0 0.5
f2 (ppm)

HMBC

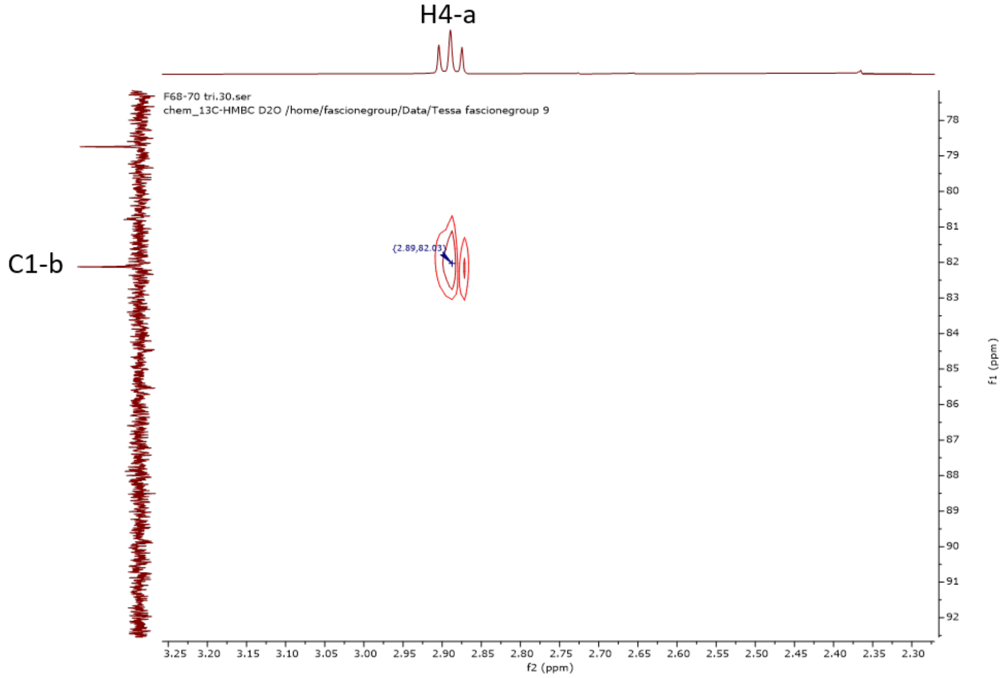
H4-a
C1-b
F68-70 tri.30.ser
chem_13C-HMBC D2O /home/fascionegroup/Data/Tessa fascionegroup 9
(2.89,82.03)
f2 (ppm)
f1 (ppm)

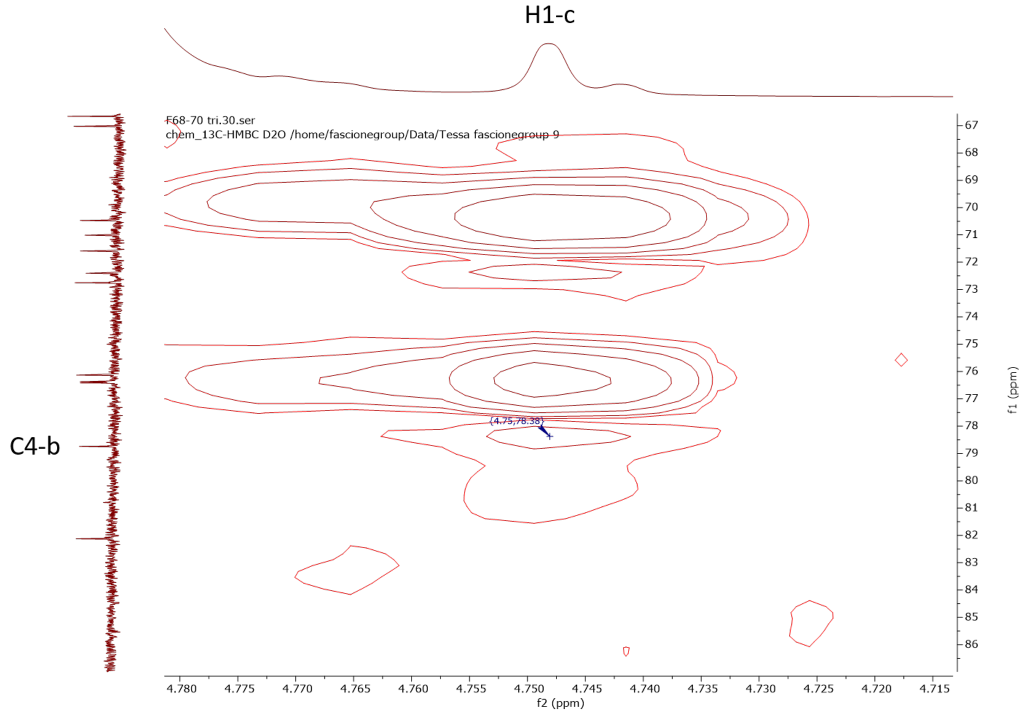
H1-c
C4-b
F68-70 tri.30.ser
chem_13C-HMBC D2O /home/fascionegroup/Data/Tessa fascionegroup 9
(4.75,78.38)
f2 (ppm)
f1 (ppm)

Man₃β1,4-S-GlcNAc-N₃ 36

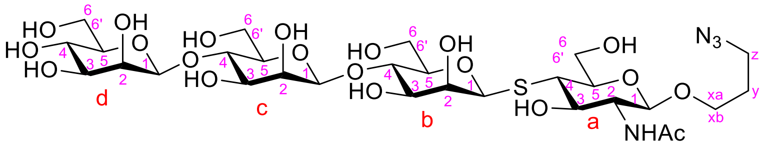

^{1}H

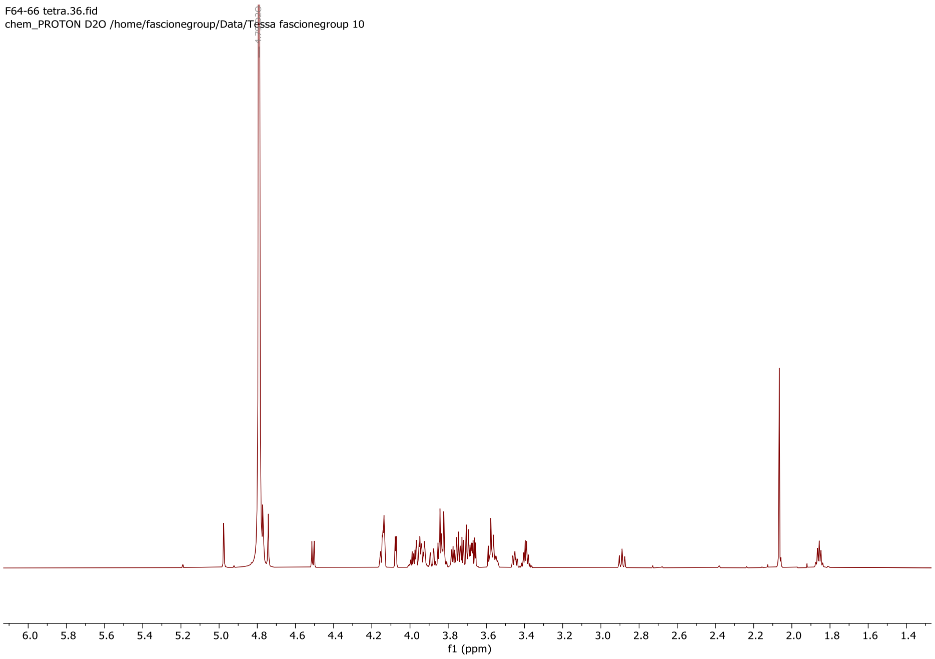

^{13}C

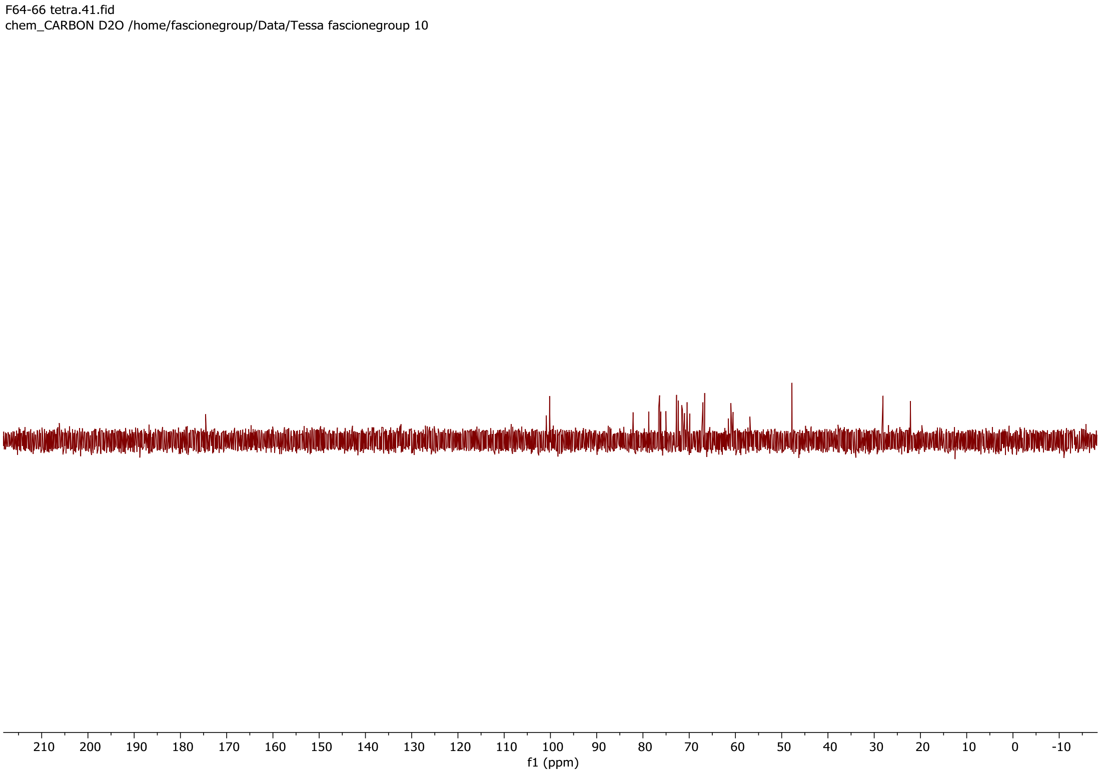

HSQC

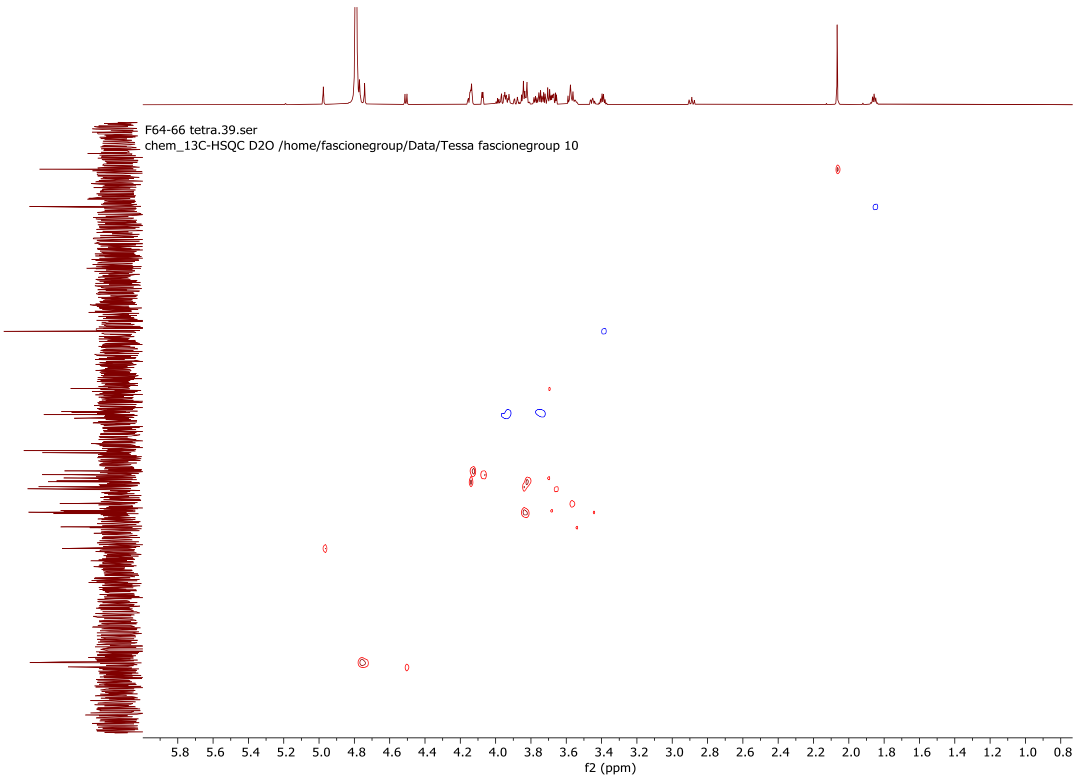

F64-66 tetra.39.ser
chem_13C-HSQC D2O /home/fascionegroup/Data/Tessa fascionegroup 10
5.8 5.6 5.4 5.2 5.0 4.8 4.6 4.4 4.2 4.0 3.8 3.6 3.4 3.2 3.0 2.8 2.6 2.4 2.2 2.0 1.8 1.6 1.4 1.2 1.0 0.8
f2 (ppm)

HMBC

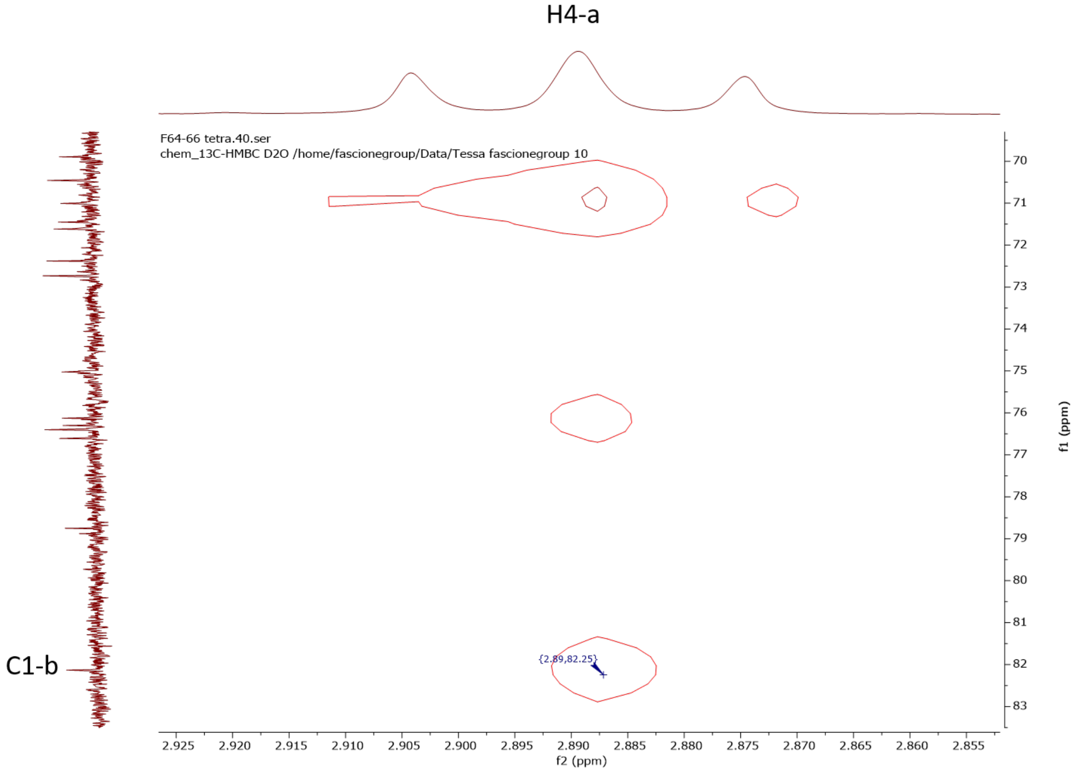

H4-a
F64-66 tetra.40.ser
chem_13C-HMBC D2O /home/fascionegroup/Data/Tessa fascionegroup 10
C1-b
{2.89,82.25}
70 71 72 73 74 75 76 77 78 79 80 81 82 83
f1 (ppm)
2.925 2.920 2.915 2.910 2.905 2.900 2.895 2.890 2.885 2.880 2.875 2.870 2.865 2.860 2.855
f2 (ppm)

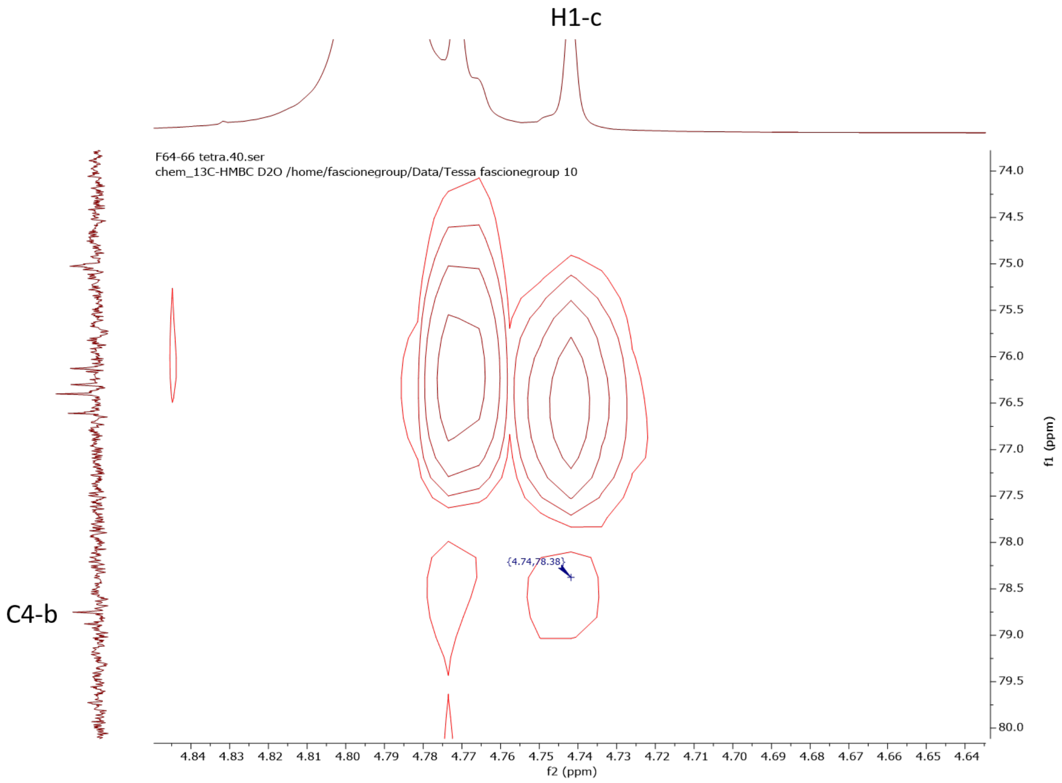
H1-c
C4-b
F64-66 tetra.40.ser
chem_13C-HMBC D2O /home/fascionegroup/Data/Tessa fascionegroup 10
{4.74,78.38}
f2 (ppm)
f1 (ppm)

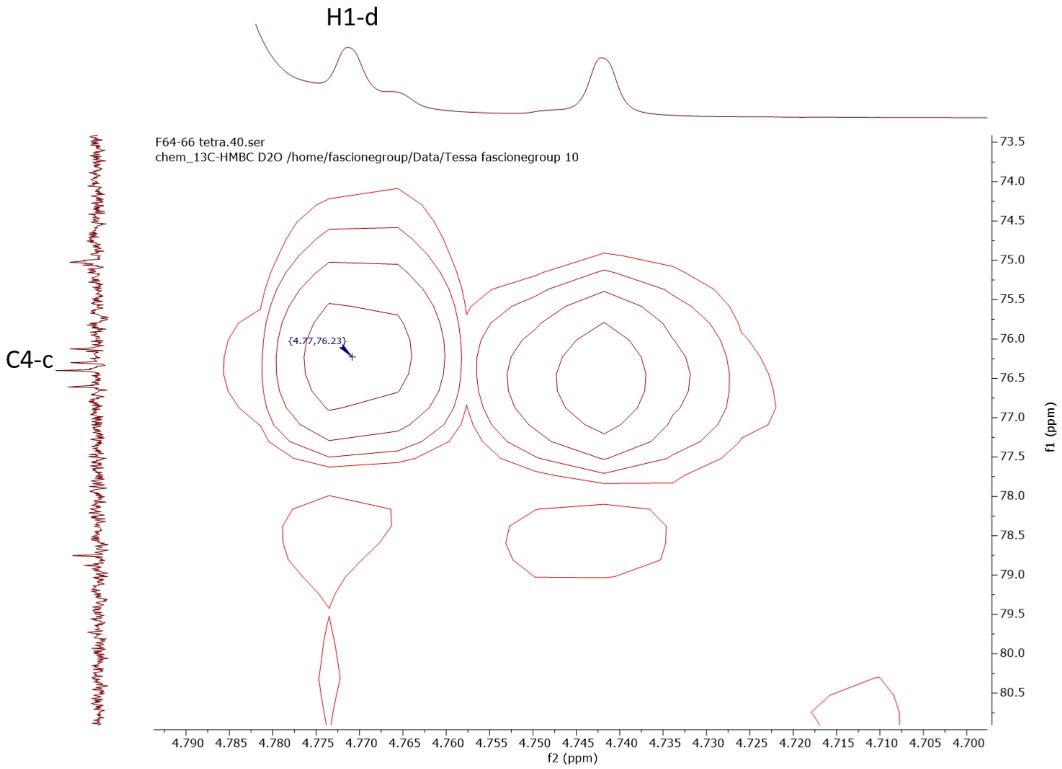
H1-d
C4-c
F64-66 tetra.40.ser
chem_13C-HMBC D2O /home/fascionegroup/Data/Tessa fascionegroup 10
{4.77,76.23}
f2 (ppm)
f1 (ppm)

Manβ1,4-6F-GlcNAc-N_3 25

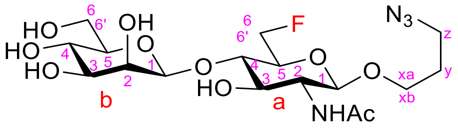

^{1}H NMR

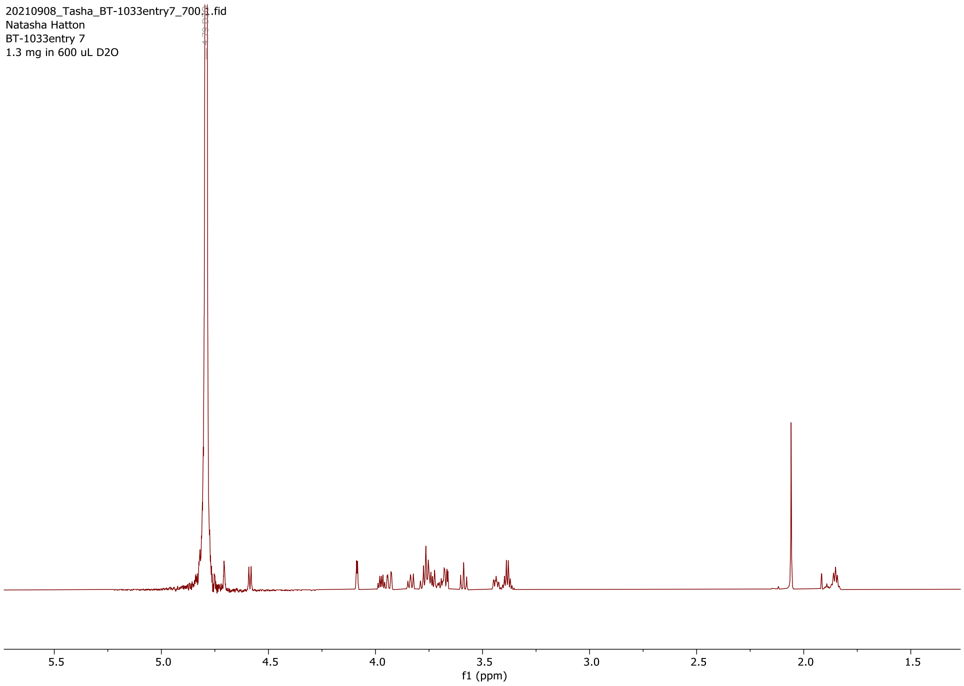

^{13}C NMR

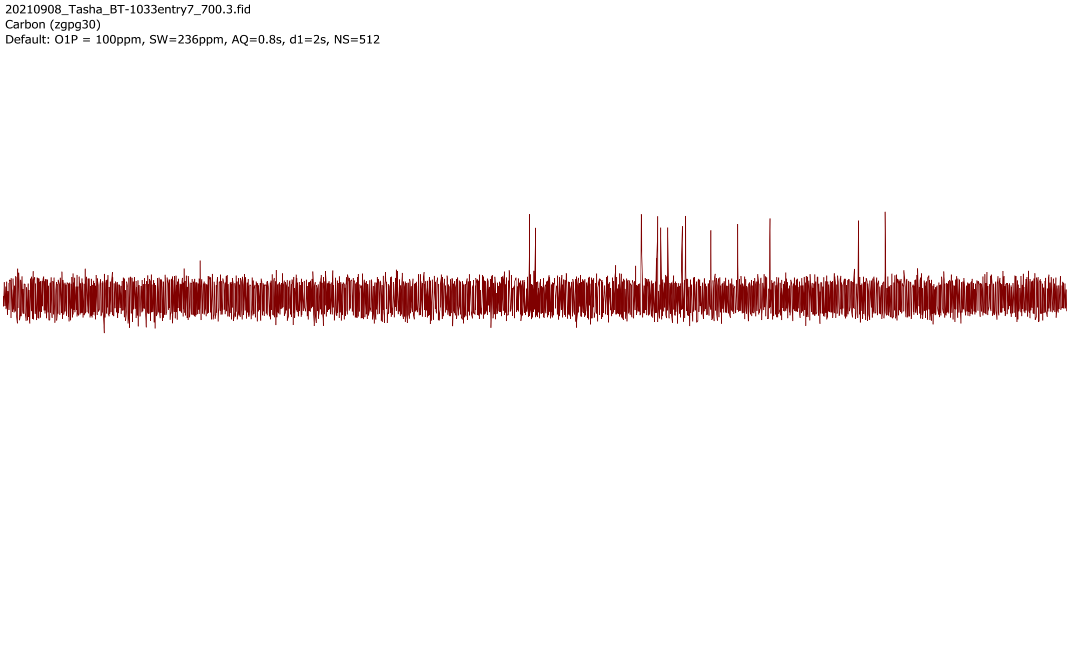

^{19}F

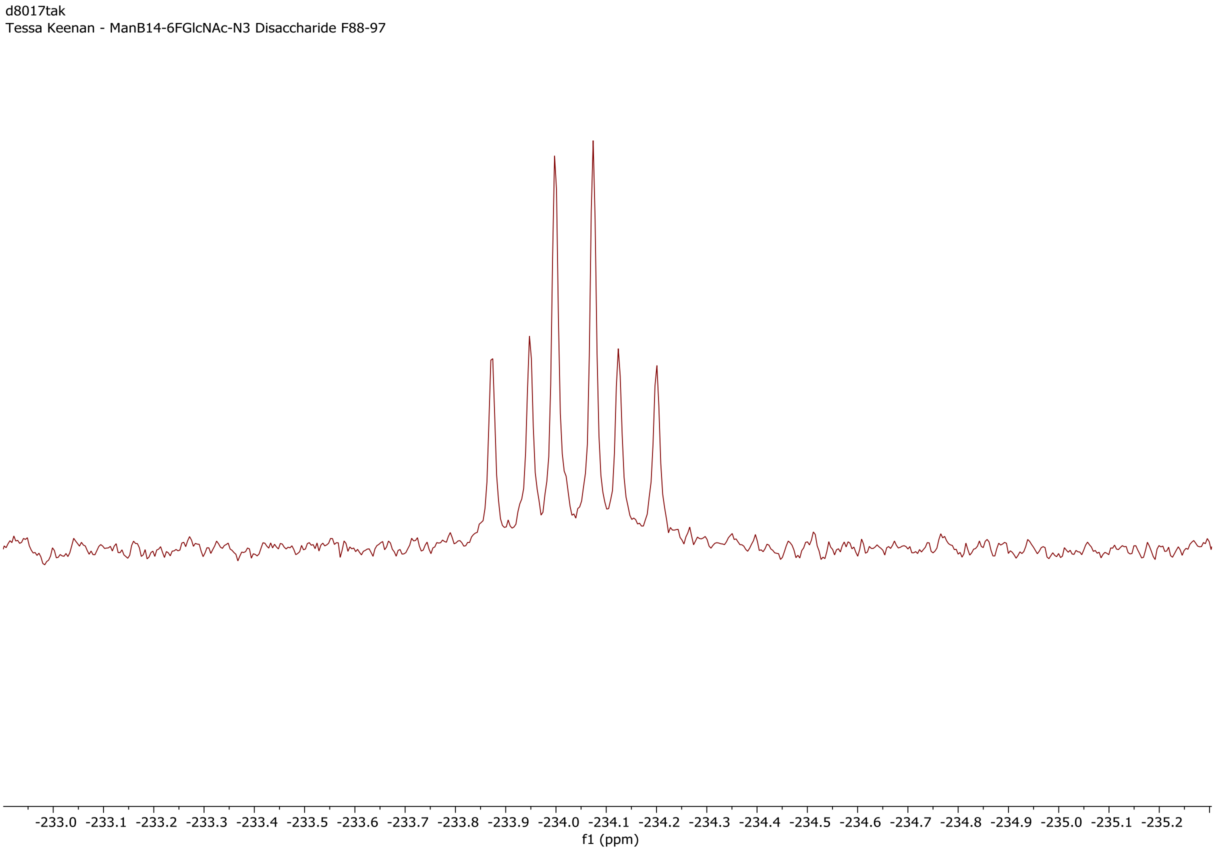

IPAP HSQC (^{13}C HSQC with multiplicity editing)

Spectrum 1

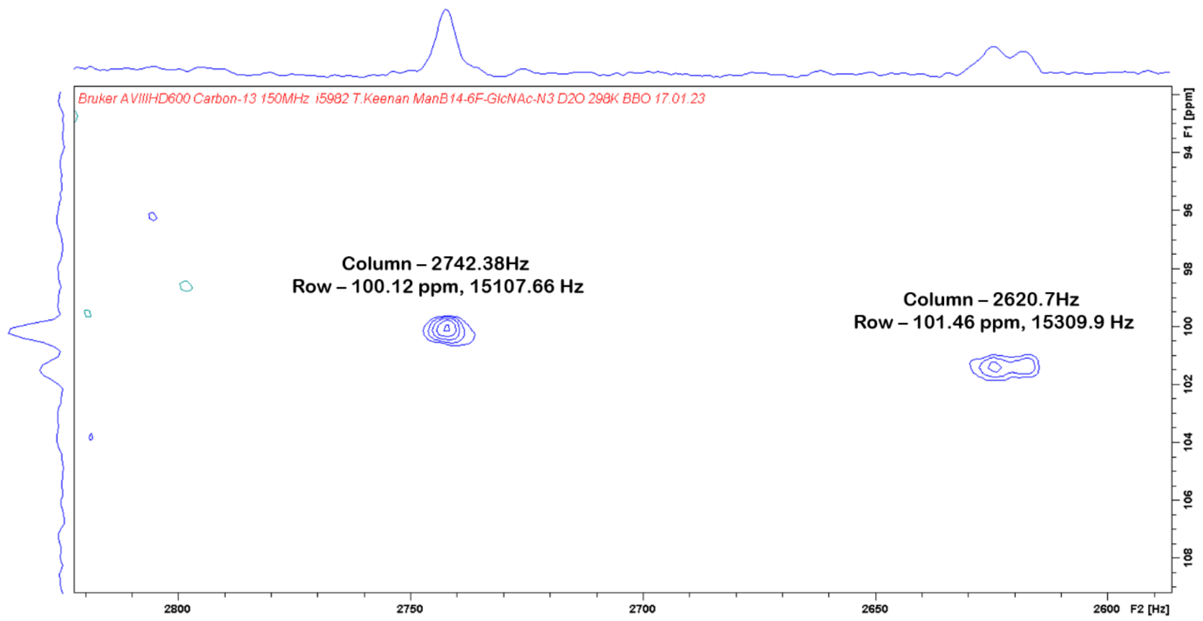

Spectrum 2

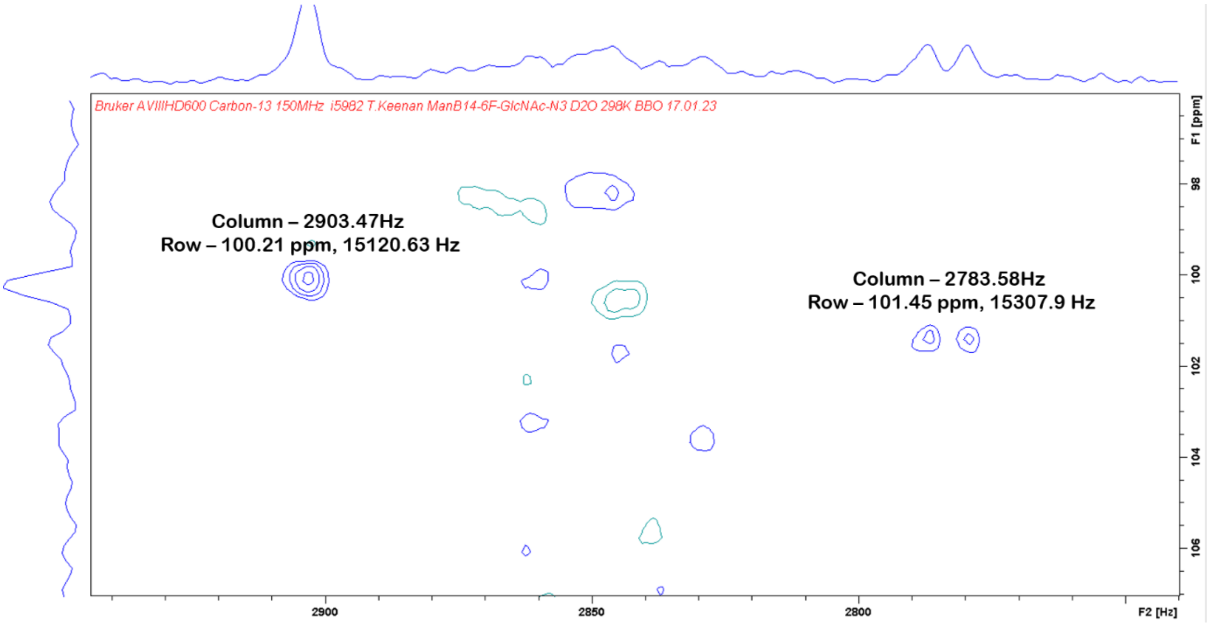

Table S4. Results of the two IPAP HSQC (13C HSQC with multiplicity editing) spectra for the two anomeric carbons and analysis of the results to give the $^{1}J_{CH}$ coupling constants.

Carbon	Spectrum one	Spectrum two	$^{1}J_{CH}$ coupling constant (Hz)
C-1 mannose	2742.38	2903.47	161.09
C-1 Glucosamine	2620.70	2783.58	162.88

Man$_2$β1,4-6F-GlcNAc-N_3 26

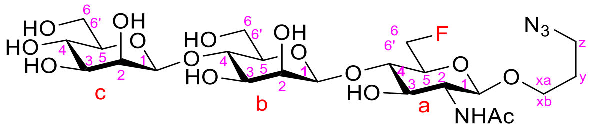

^1H

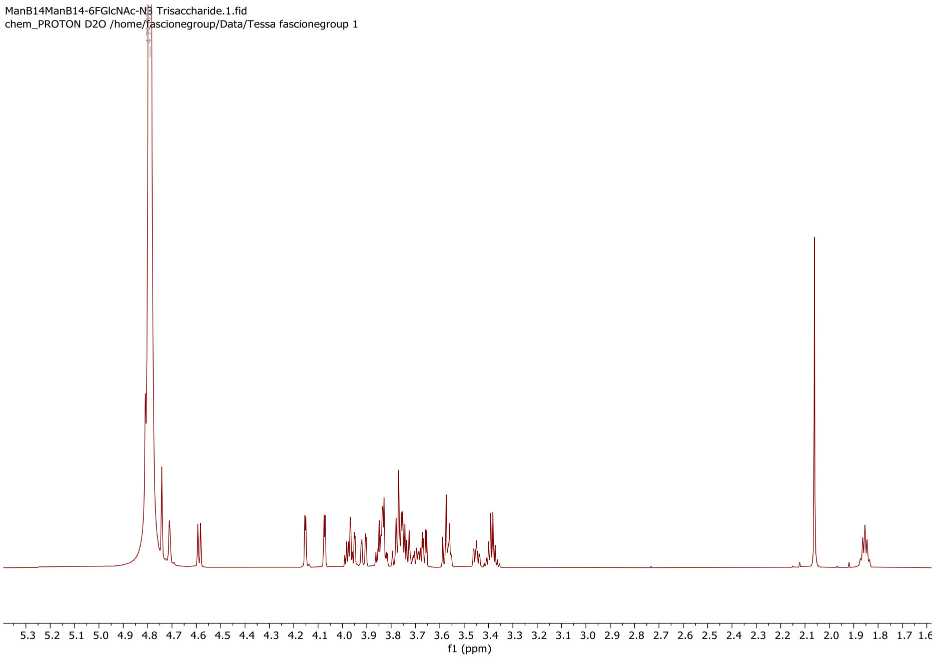

^{13}C

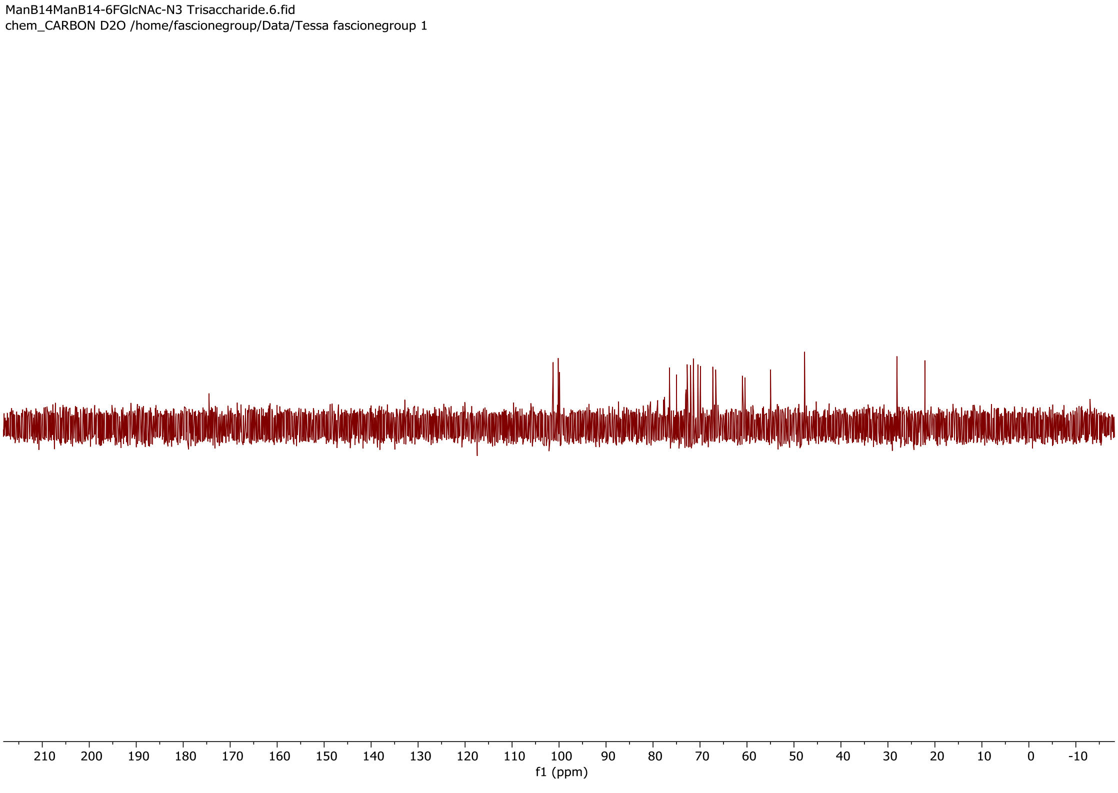

^{19}F

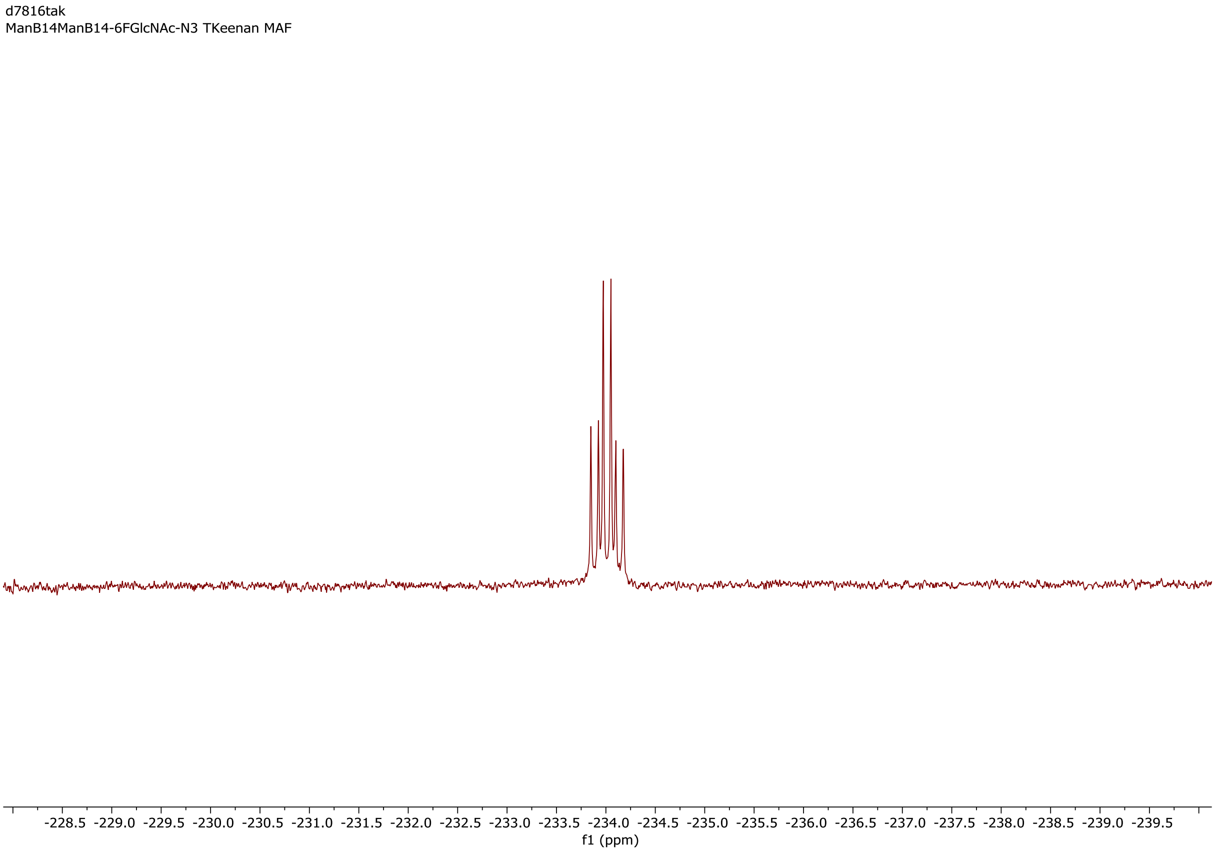

d7816tak
ManB14ManB14-6FGlcNAc-N3 TKeenan MAF
-228.5 -229.0 -229.5 -230.0 -230.5 -231.0 -231.5 -232.0 -232.5 -233.0 -233.5 -234.0 -234.5 -235.0 -235.5 -236.0 -236.5 -237.0 -237.5 -238.0 -238.5 -239.0 -239.5
f1 (ppm)

HSQC

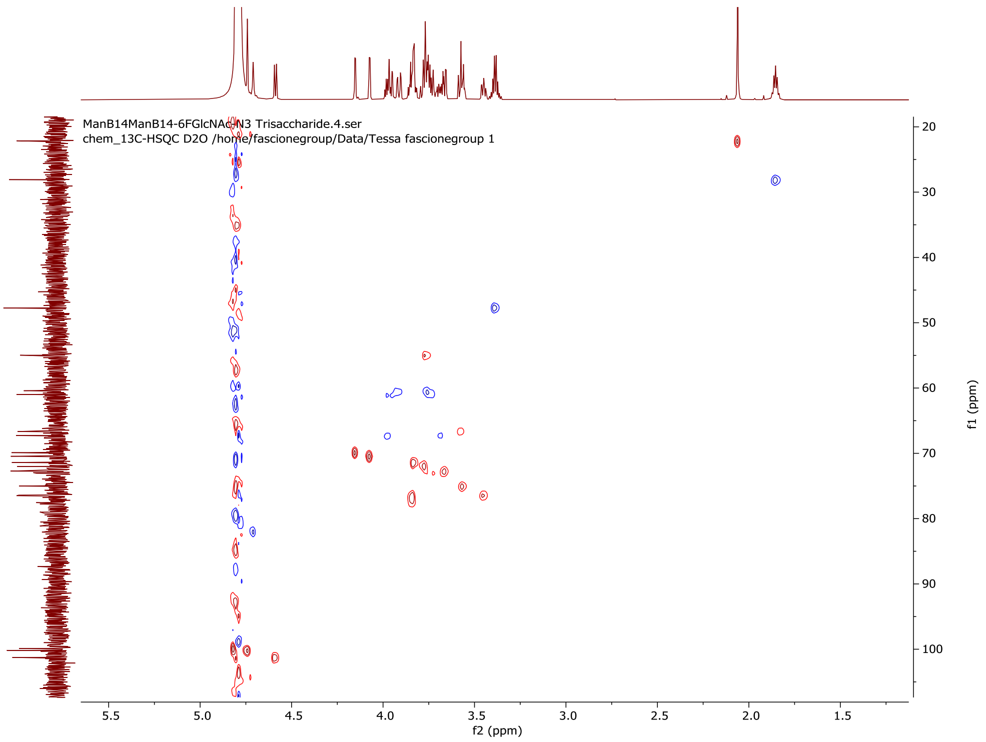

ManB14ManB14-6FGlcNAc-N3 Trisaccharide.4.ser
chem_13C-HSQC D2O /home/fascionegroup/Data/Tessa fascionegroup 1
5.5 5.0 4.5 4.0 3.5 3.0 2.5 2.0 1.5
f2 (ppm)
20 30 40 50 60 70 80 90 100
f1 (ppm)

HMBC

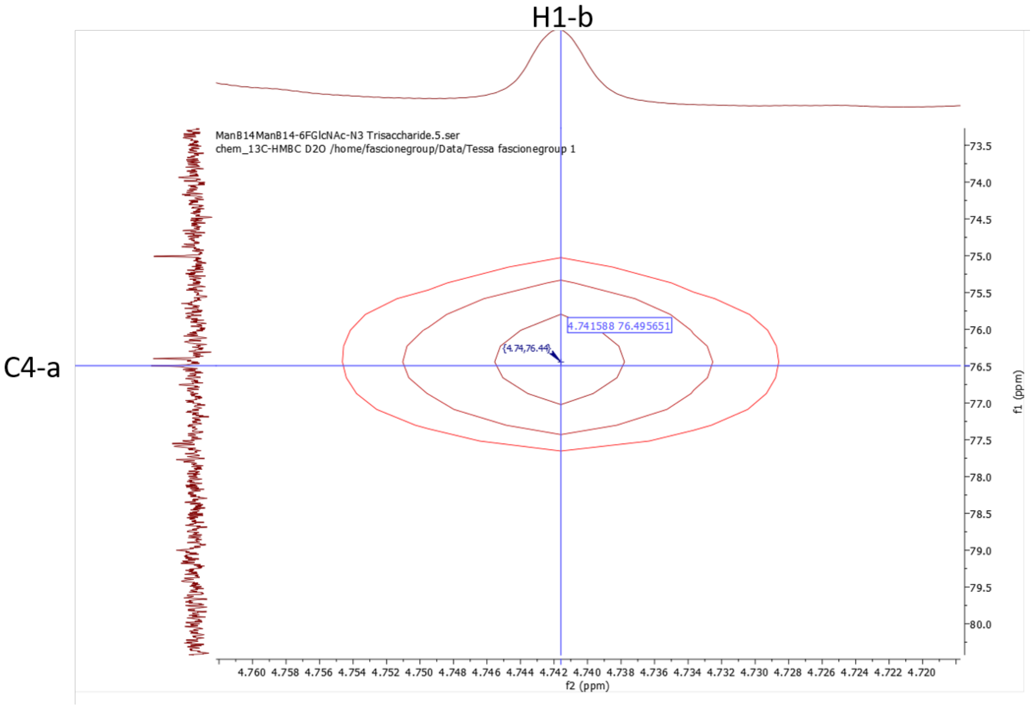

Man₃β1,4-6F-GlcNAc-N₃ 27

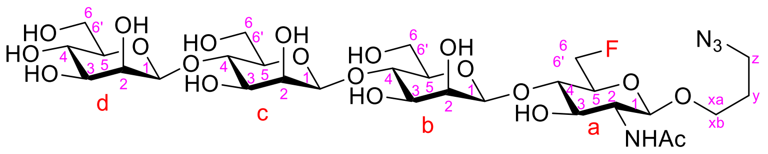

^1H

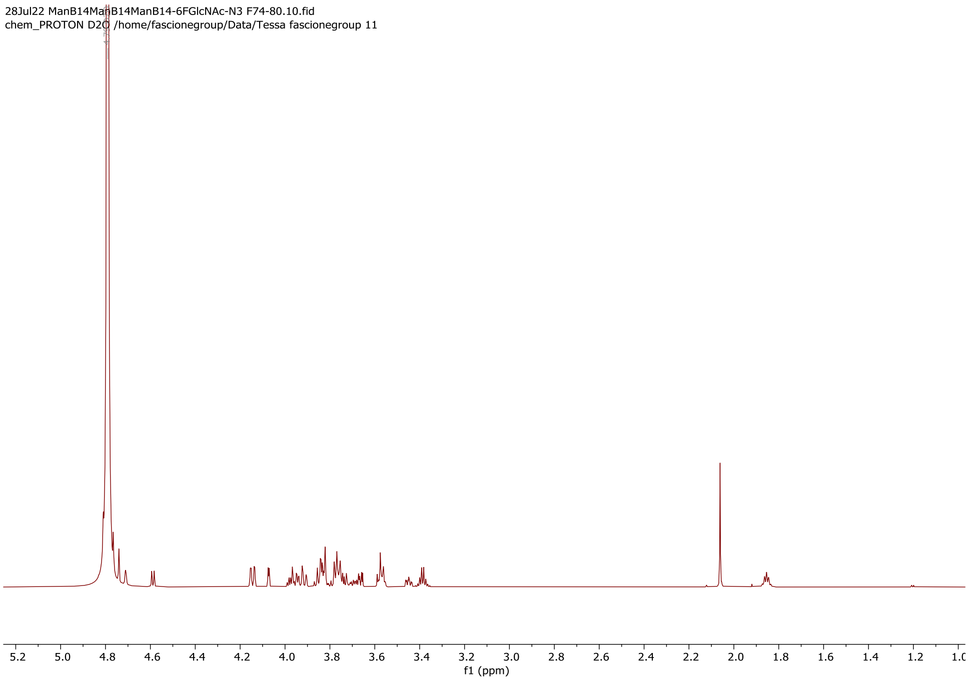

^{13}C

17Aug22_ManB14ManB14ManB14-6FGlcNAc-3 F74-80 rep carbon.1.fid
17Aug22_ManB14ManB14ManB14-6FGlcNAc-3 F74-80 rep carbon

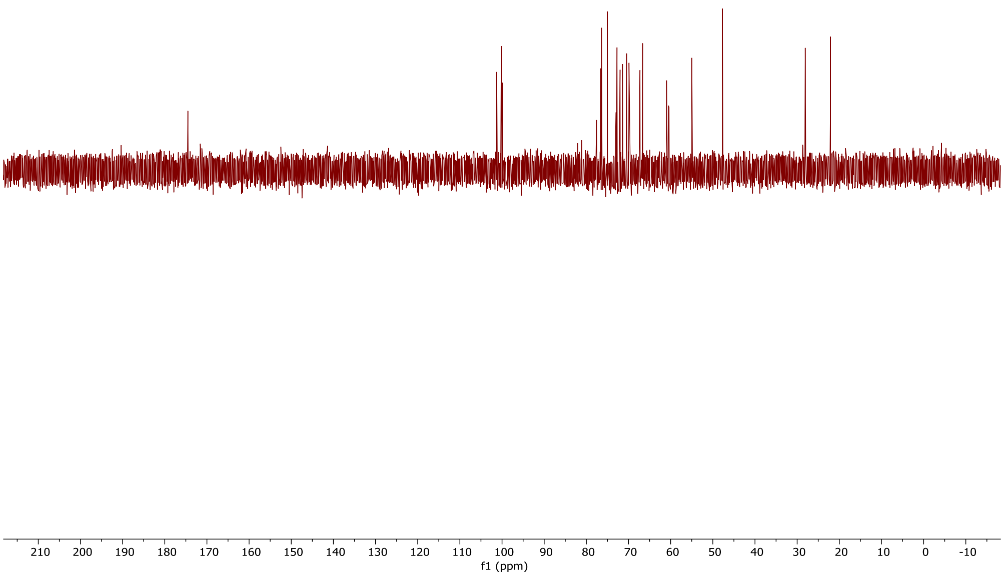

^{19}F

d8747tak
Tessa Keenan - ManB14ManB14ManB14-6FGlcNAc-N3 F74-80

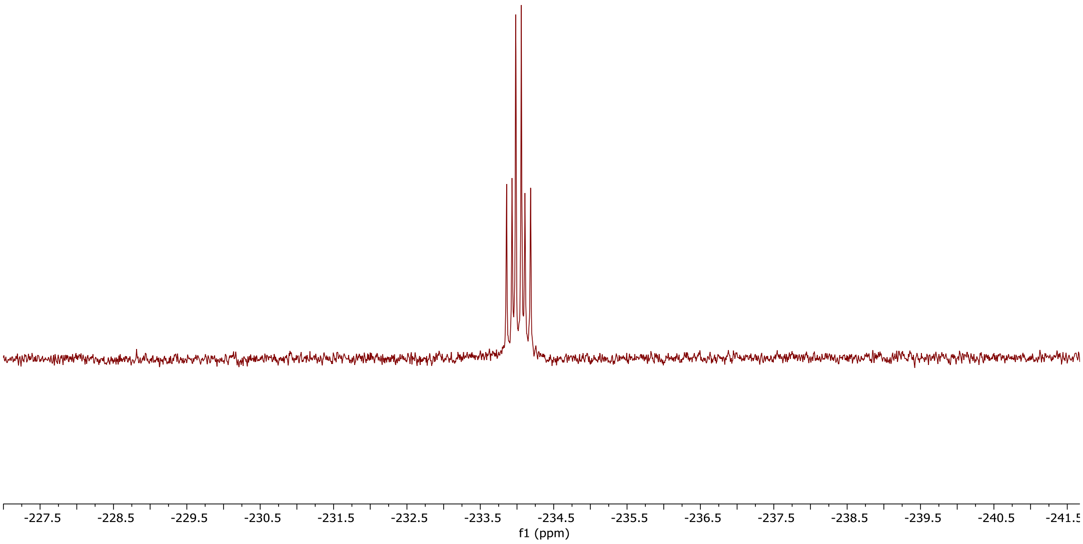

HSQC

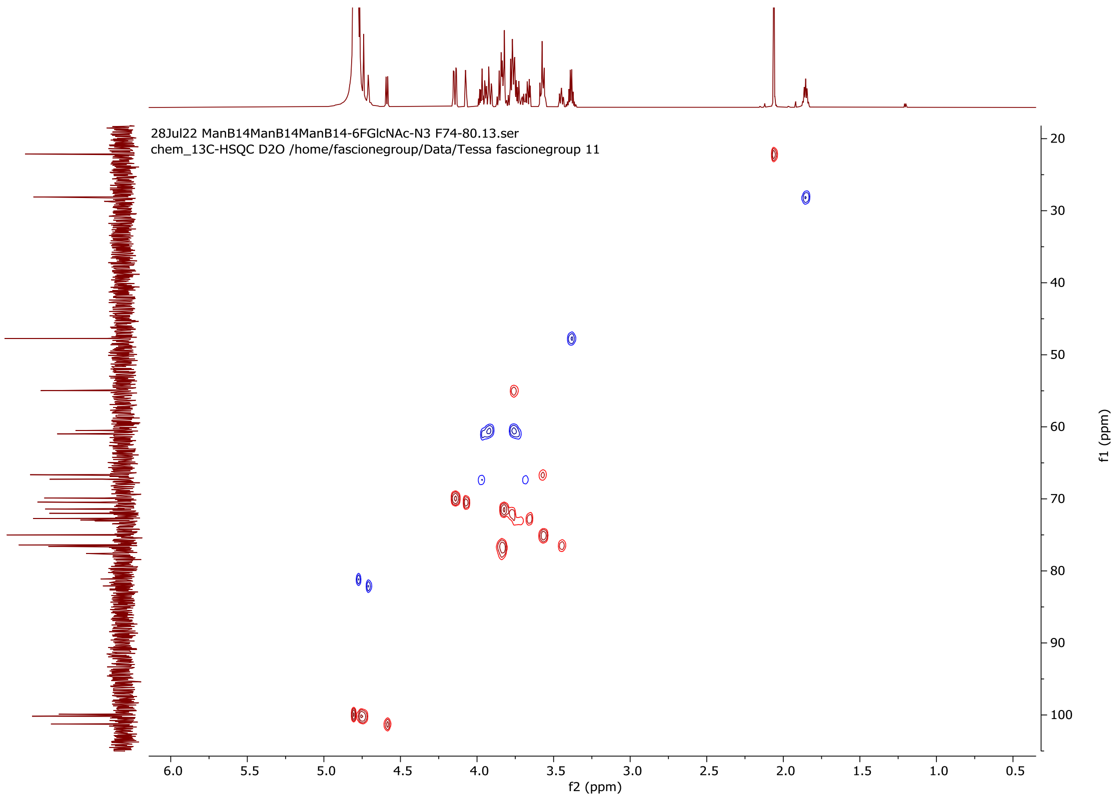

$Man_4β1,4$-6F-GlcNAc-N_3 37

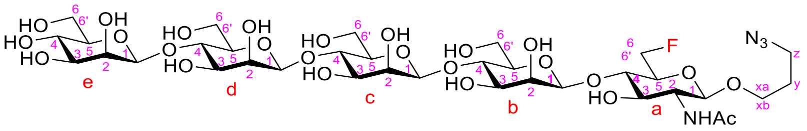

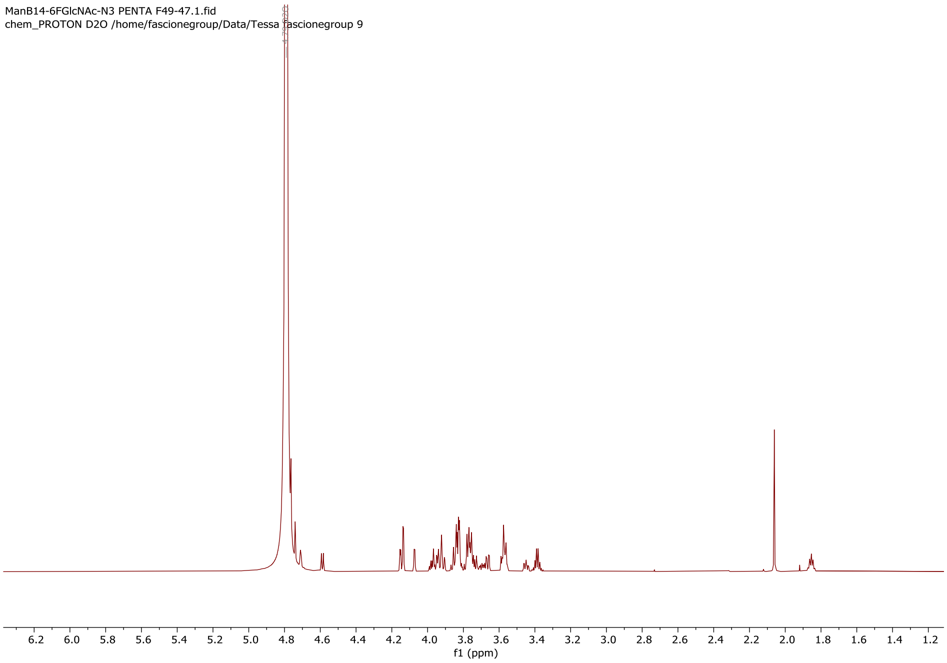

DEPT ^{13}C

ManB14-6FGlcNAc-N3 Penta F49-47 REPEAT CARBONS.3.fid
chem_DEPT-135 D2O /home/fascionegroup/Data/Tessa fascionegroup 9

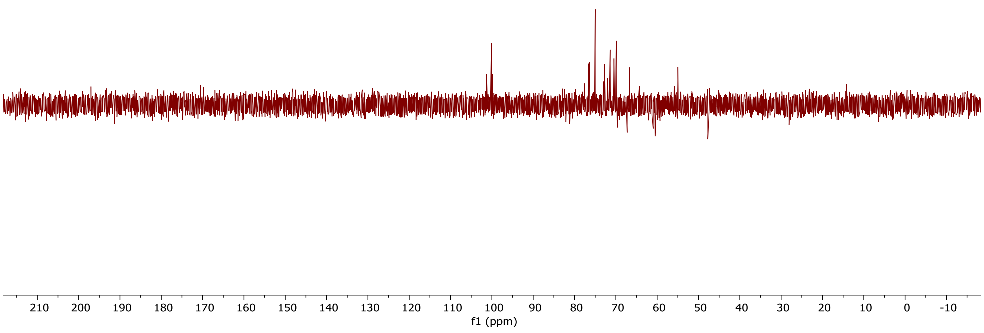

^{1}H- ^{1}H COSY

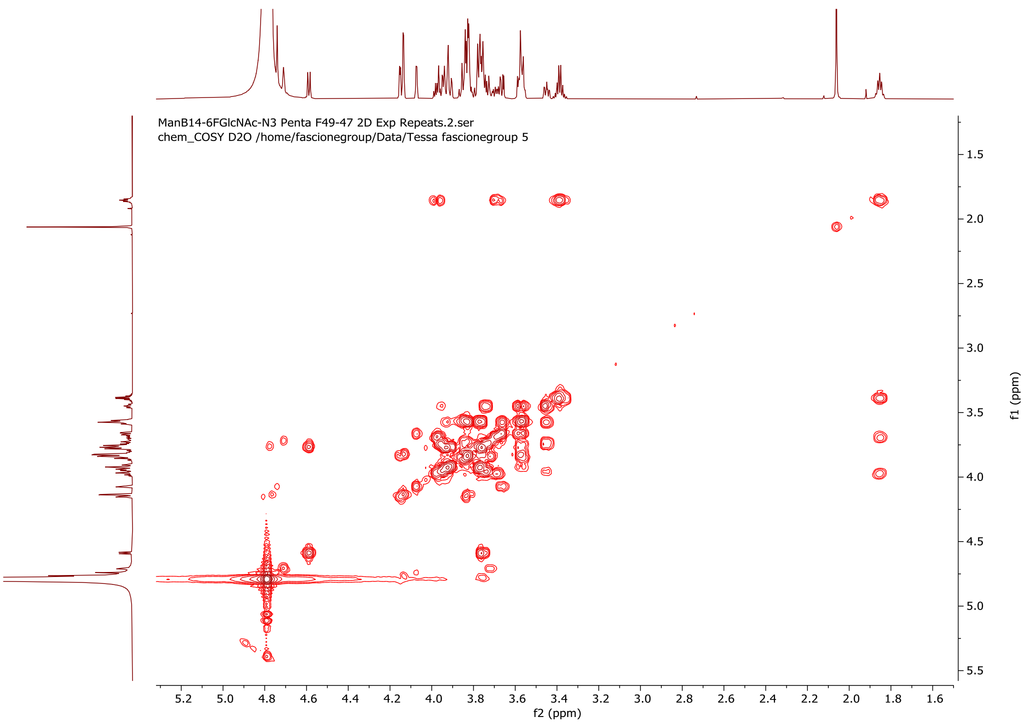

^{1}H-^{13}C HSQC

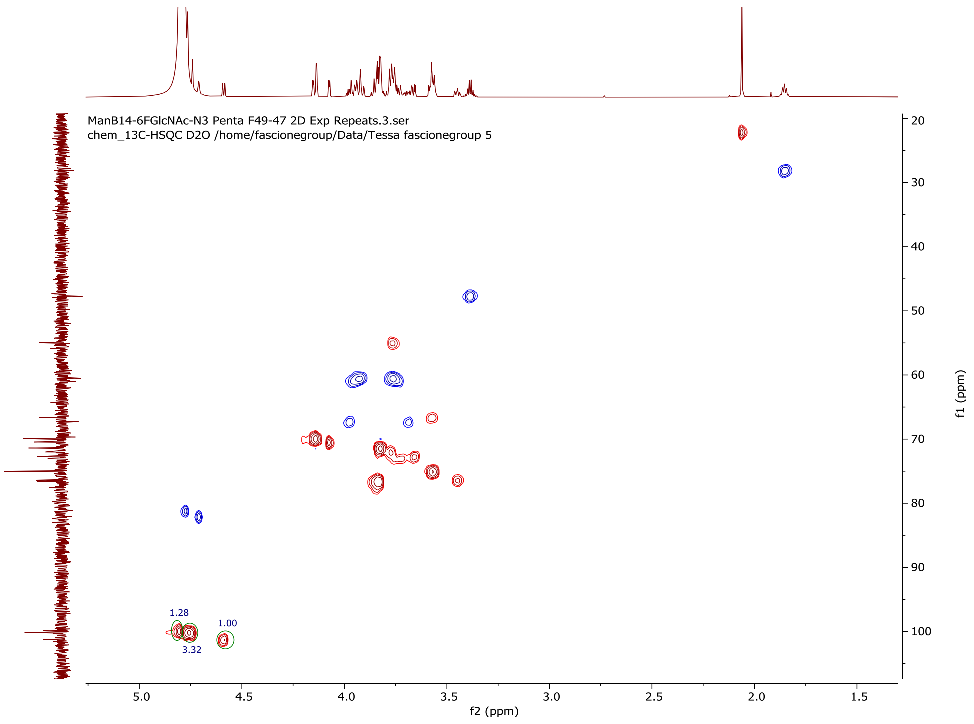

ManB14-6FGlcNAc-N3 Penta F49-47 2D Exp Repeats.3.ser
chem_13C-HSQC D2O /home/fascionegroup/Data/Tessa fascionegroup 5
1.28
1.00
3.32
f2 (ppm)
f1 (ppm)

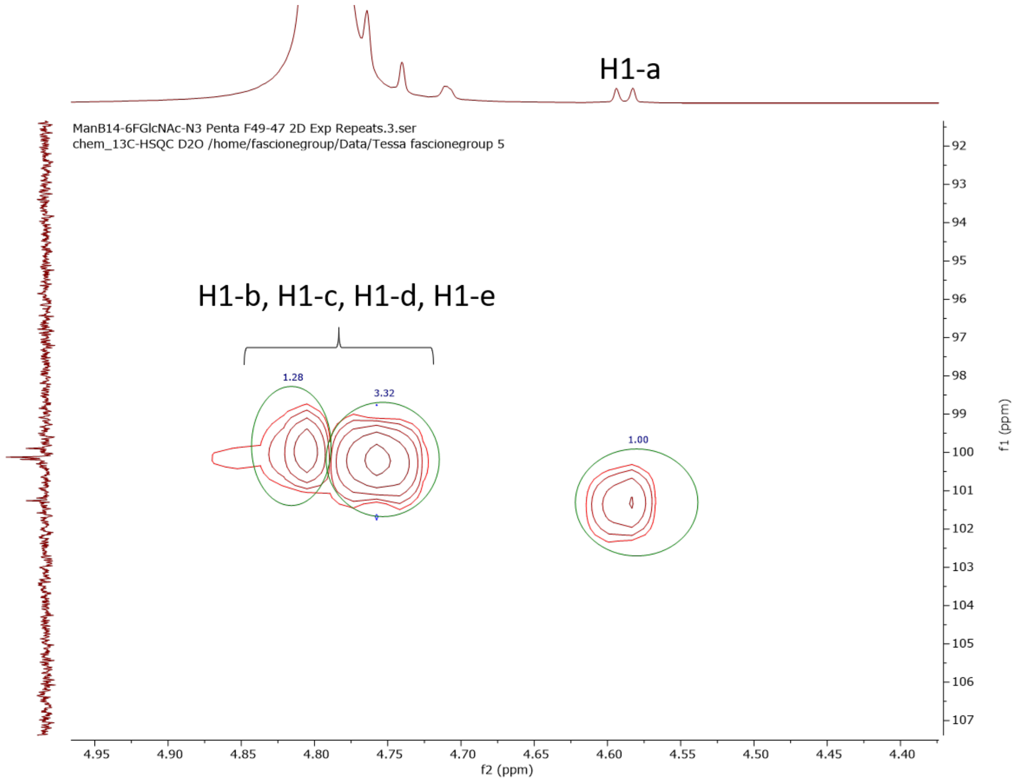

H1-a
ManB14-6FGlcNAc-N3 Penta F49-47 2D Exp Repeats.3.ser
chem_13C-HSQC D2O /home/fascionegroup/Data/Tessa fascionegroup 5
H1-b, H1-c, H1-d, H1-e
1.28
3.32
1.00
f2 (ppm)
f1 (ppm)

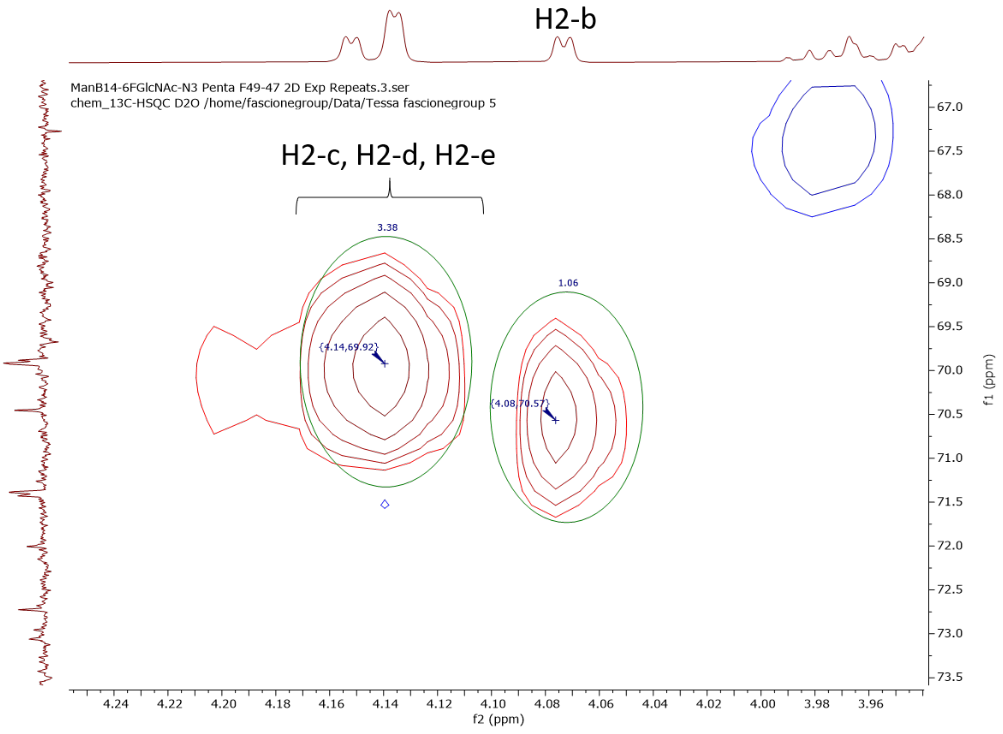
H2-b
ManB14-6FGlcNAc-N3 Penta F49-47 2D Exp Repeats.3.ser
chem_13C-HSQC D2O /home/fascionegroup/Data/Tessa fascionegroup 5
H2-c, H2-d, H2-e
3.38
1.06
{4.14,69.92}
{4.08,70.57}
f2 (ppm)
f1 (ppm)

^{19}F

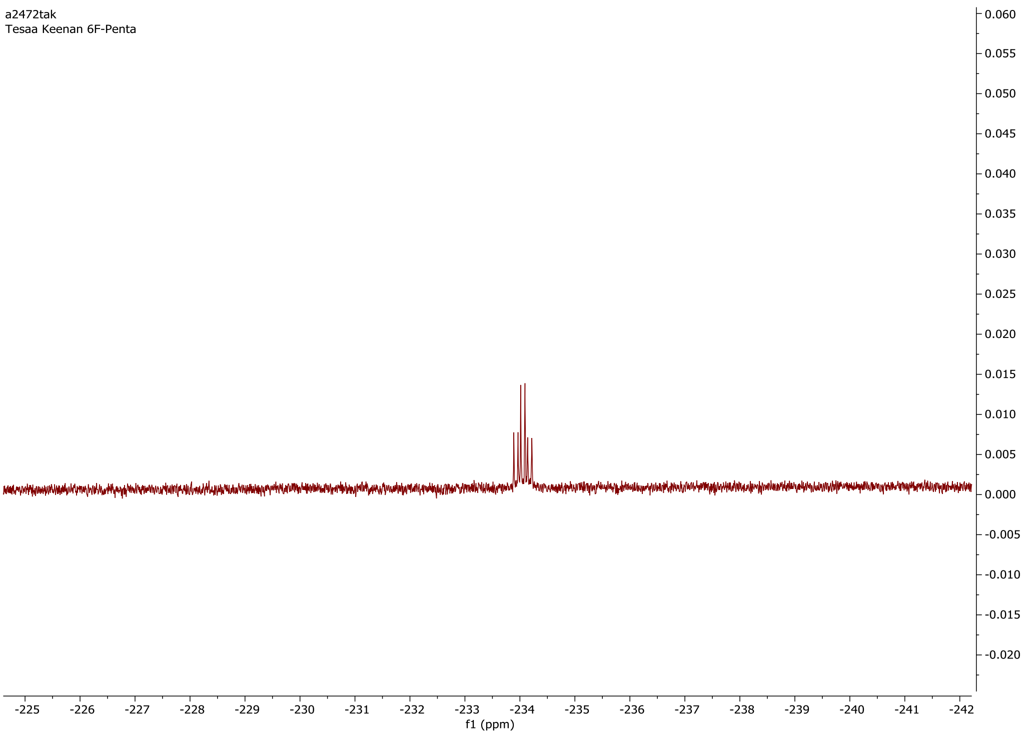
a2472tak
Tesaa Keenan 6F-Penta
f1 (ppm)

2-acetamido-1,3,4,6-tetra-O-acetyl-2-deoxy-β-D-galactopyranoside S2

^{1}H

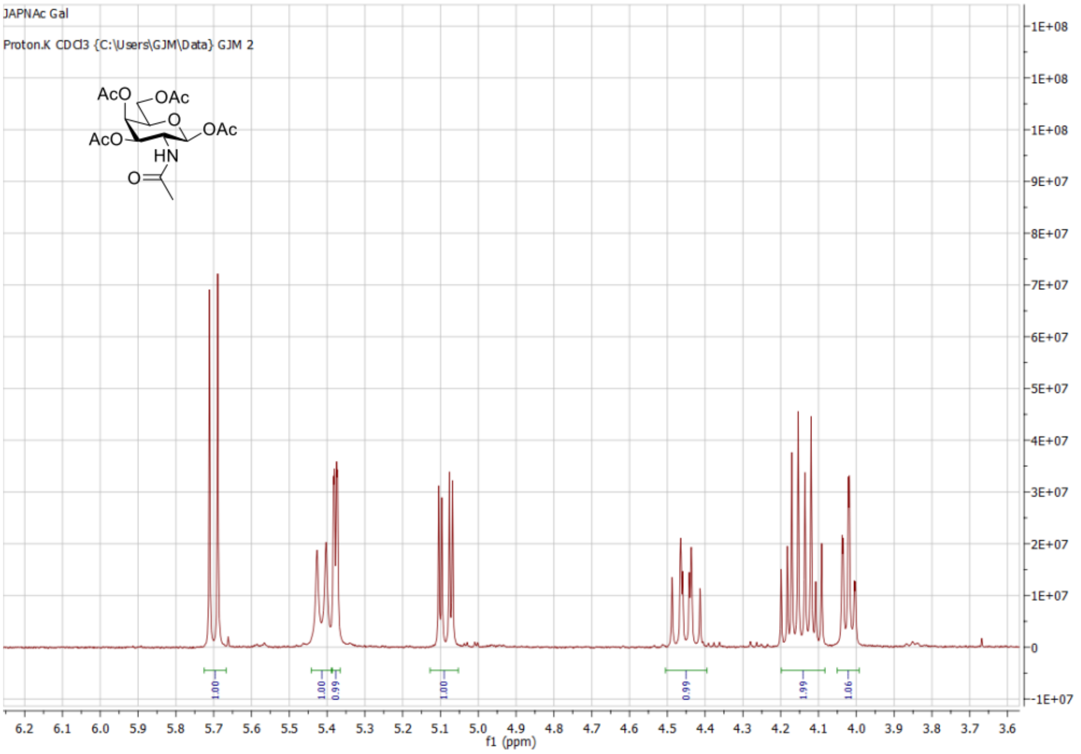

^{13}C

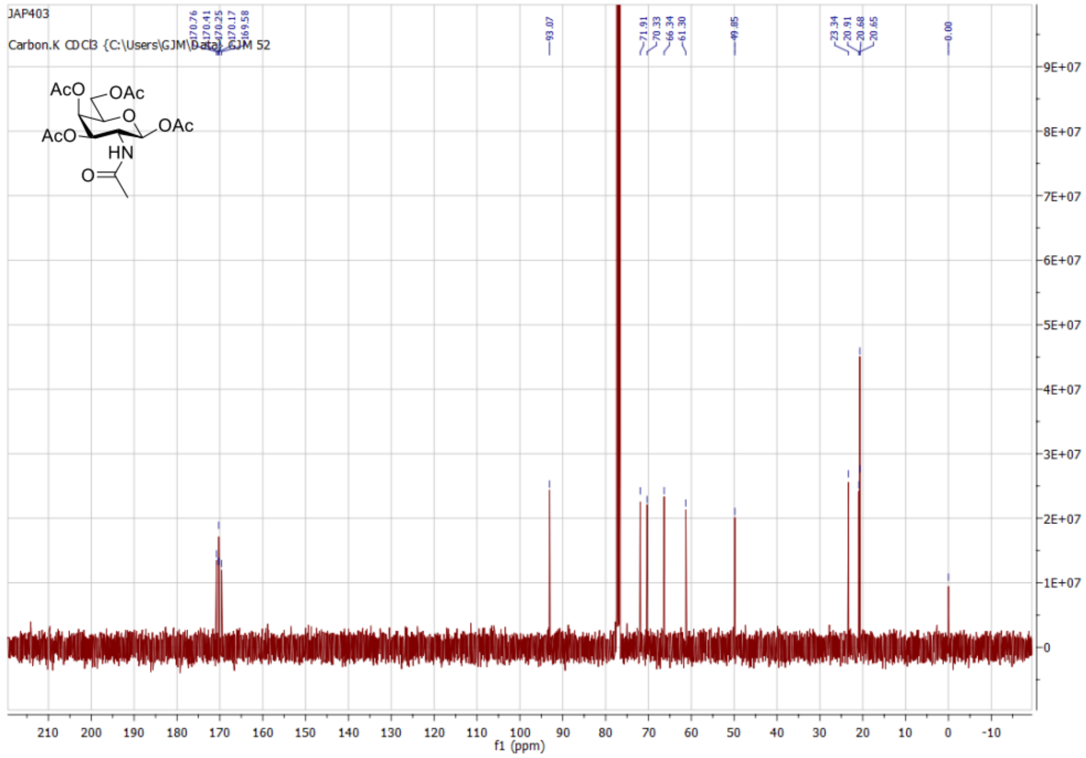

2-Methyl-4,5-dihydro-(3,4,6-tri-O-acetyl-1,2-dideoxy-α-D galactopyranoso)[2,1- d]-1,3-oxazole S3

^{1}H

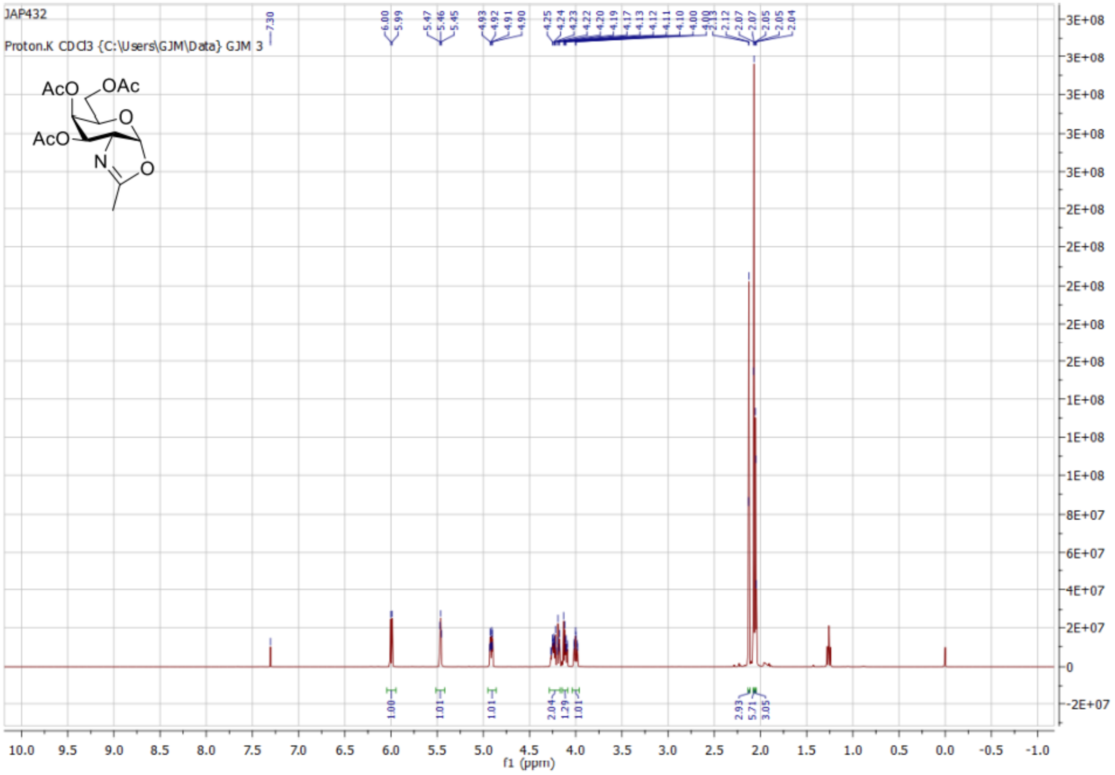

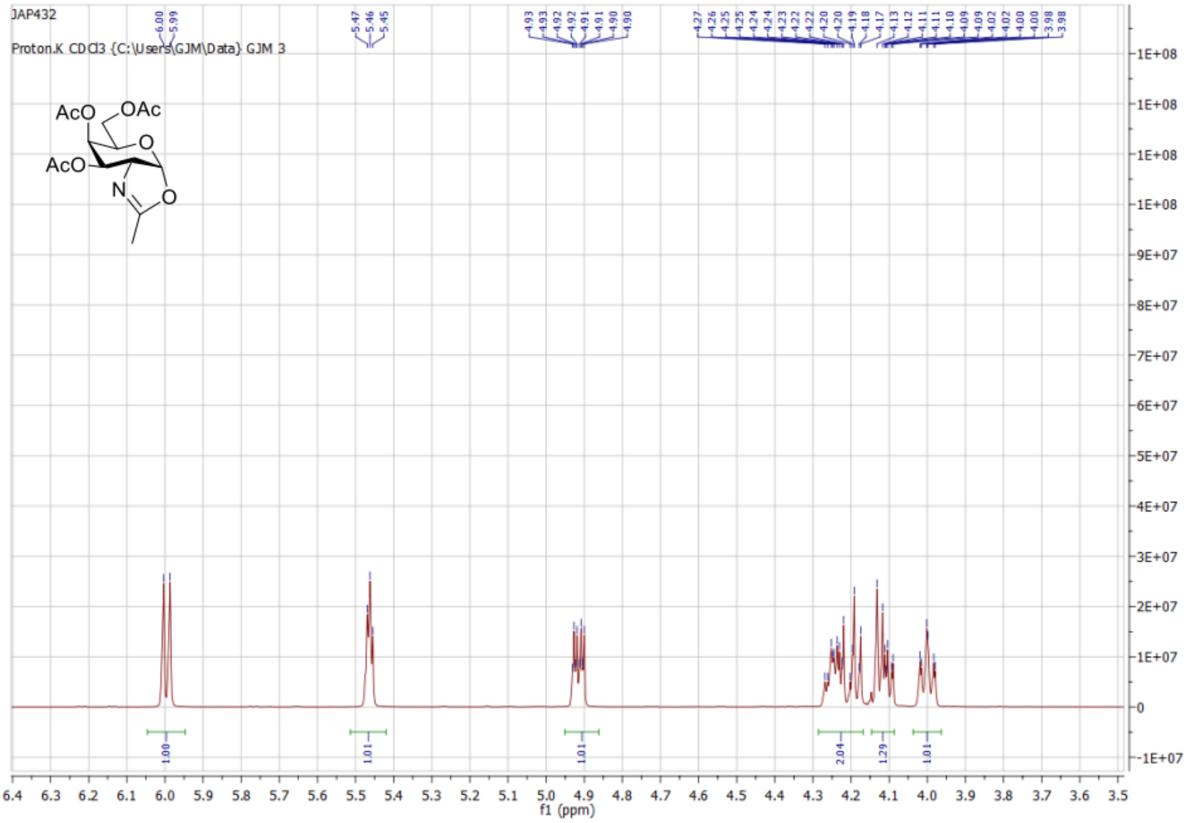

^{13}C

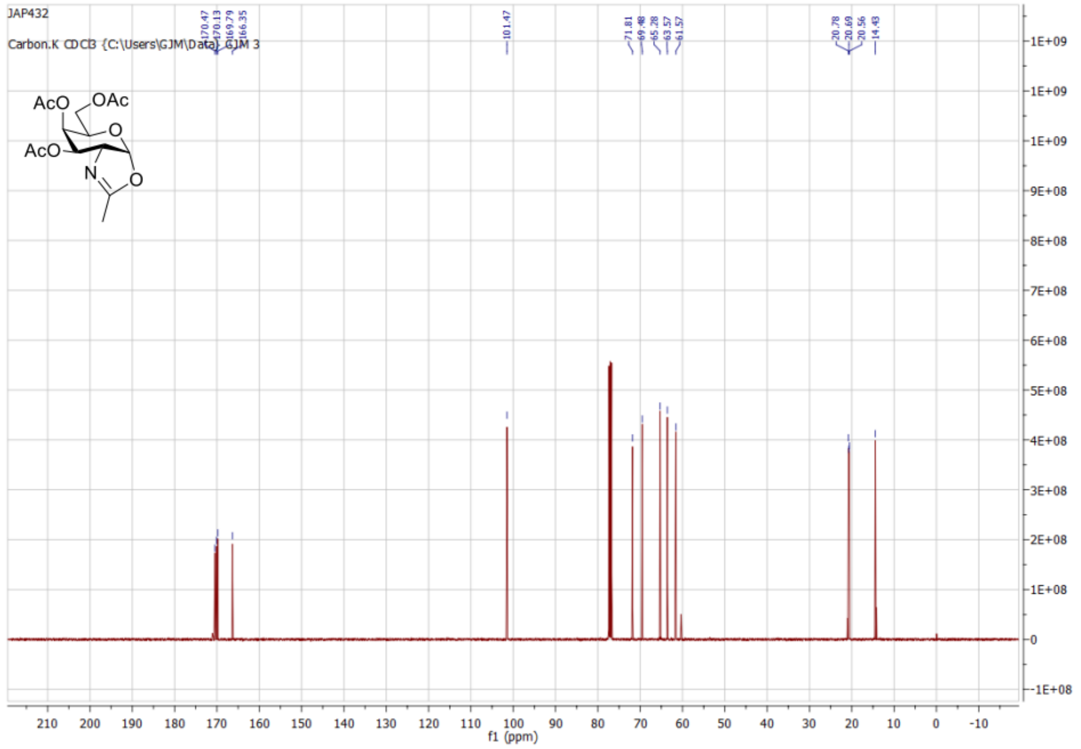

3-Bromopropyl (2-acetamido-3,4,6-tri-O-acetyl-2-deoxy)-β-D-galactopyranoside S4

^{1}H

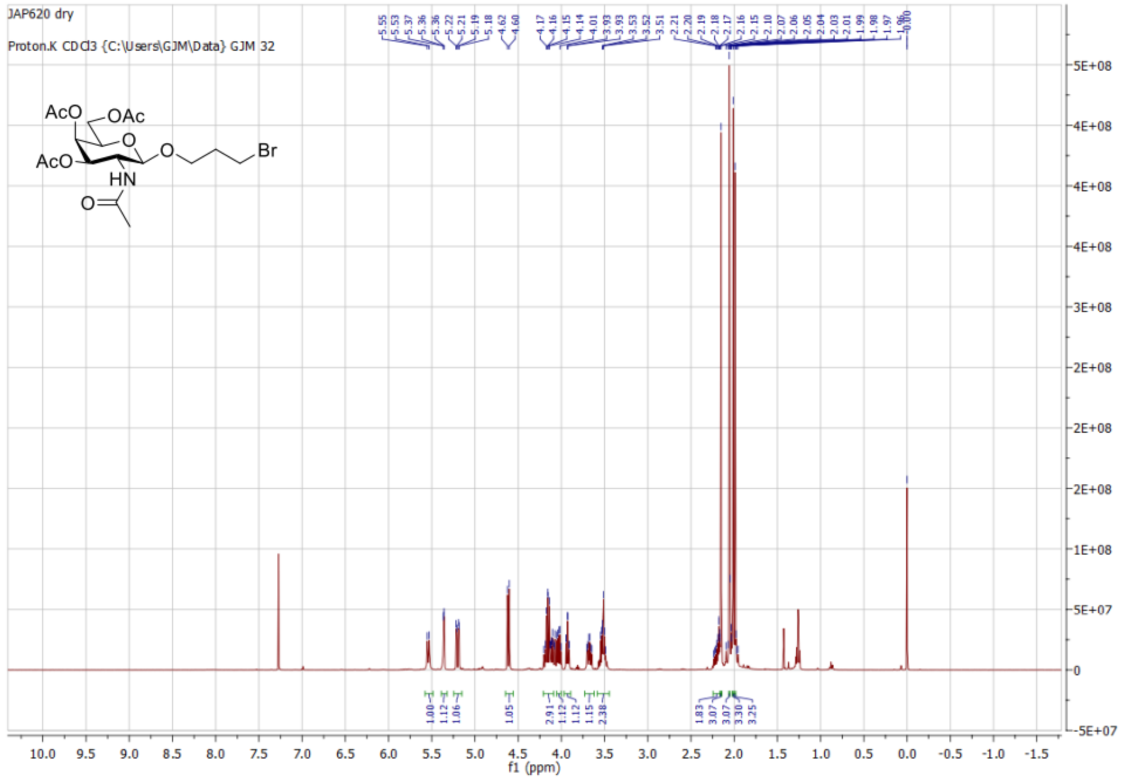

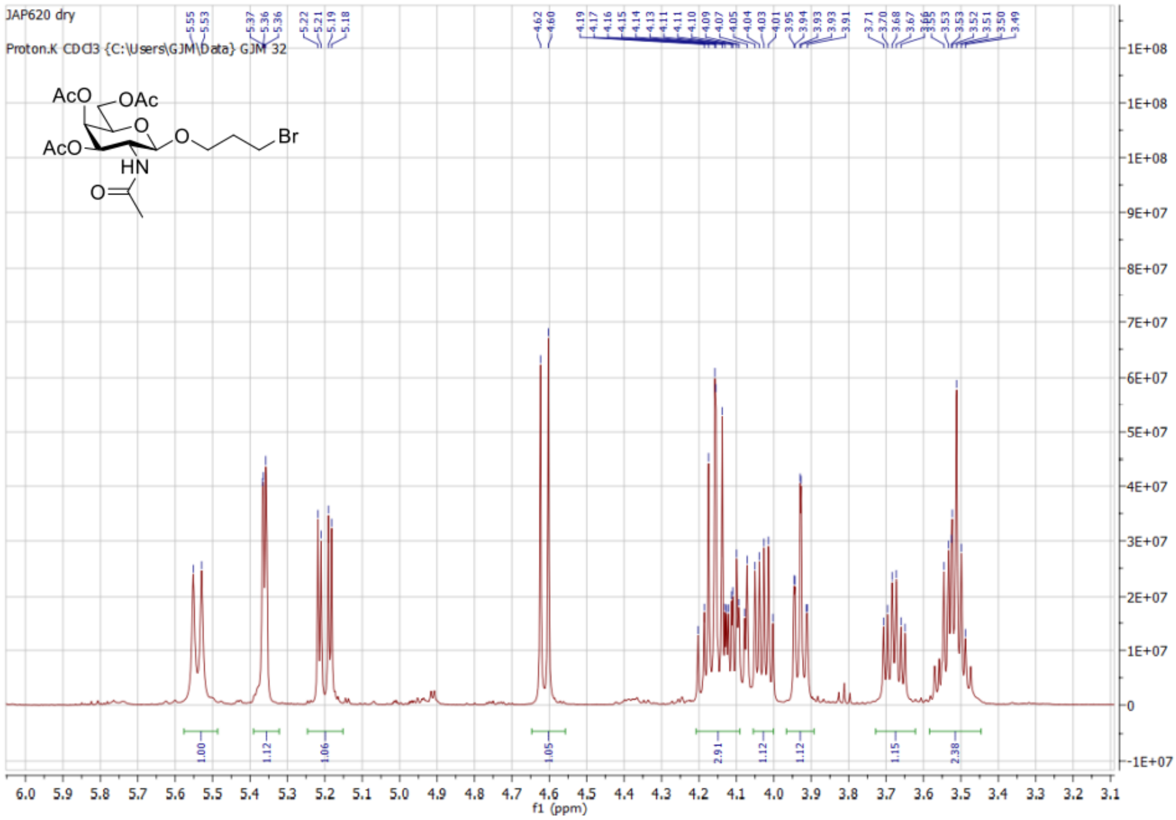
JAP620 dry
Proton.K CDCl3 {C:\Users\GJM\Data} GJM 32
AcO
OAc
AcO
HN
Br
f1 (ppm)

^{13}C

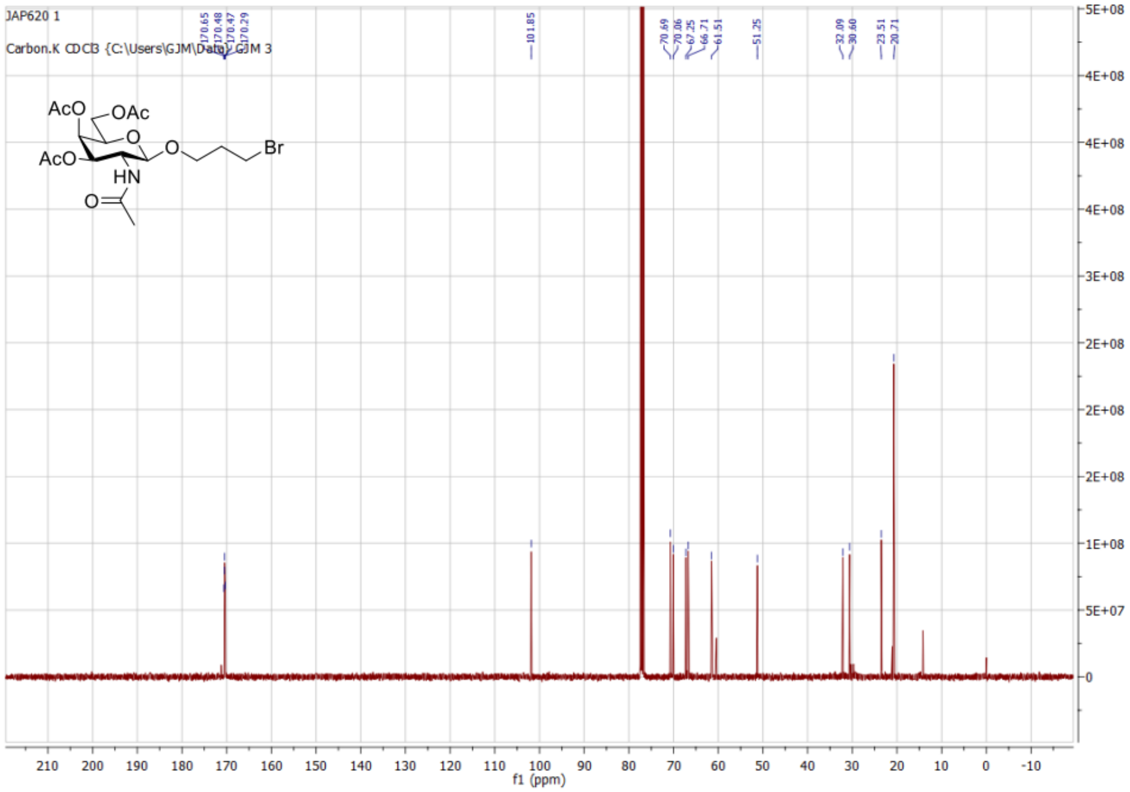
JAP620 1
Carbon.K CDCl3 {C:\Users\GJM\Data} GJM 3
AcO
OAc
AcO
HN
Br
f1 (ppm)

COSY

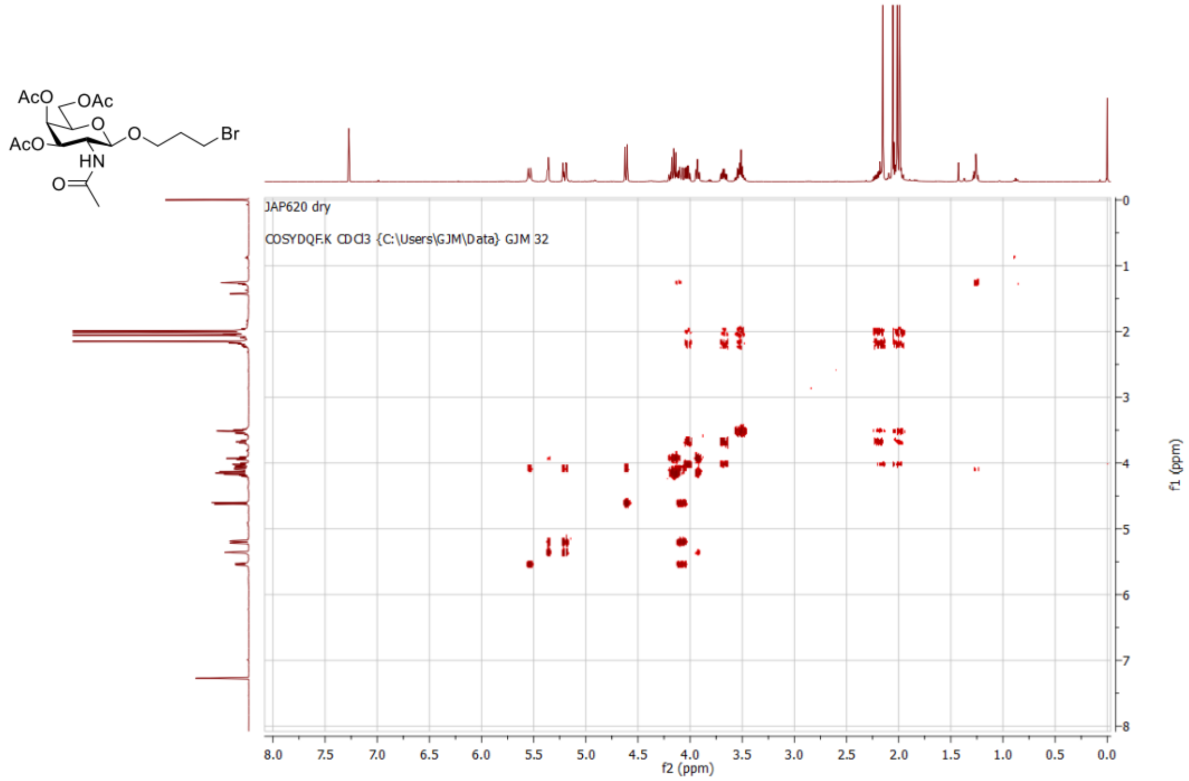
AcO
OAc
O
O
Br
AcO
HN
O
JAP620 dry
COSYDQF.K CDCl3 {C:\Users\GJM\Data} GJM 32
f2 (ppm)
f1 (ppm)

HSQC

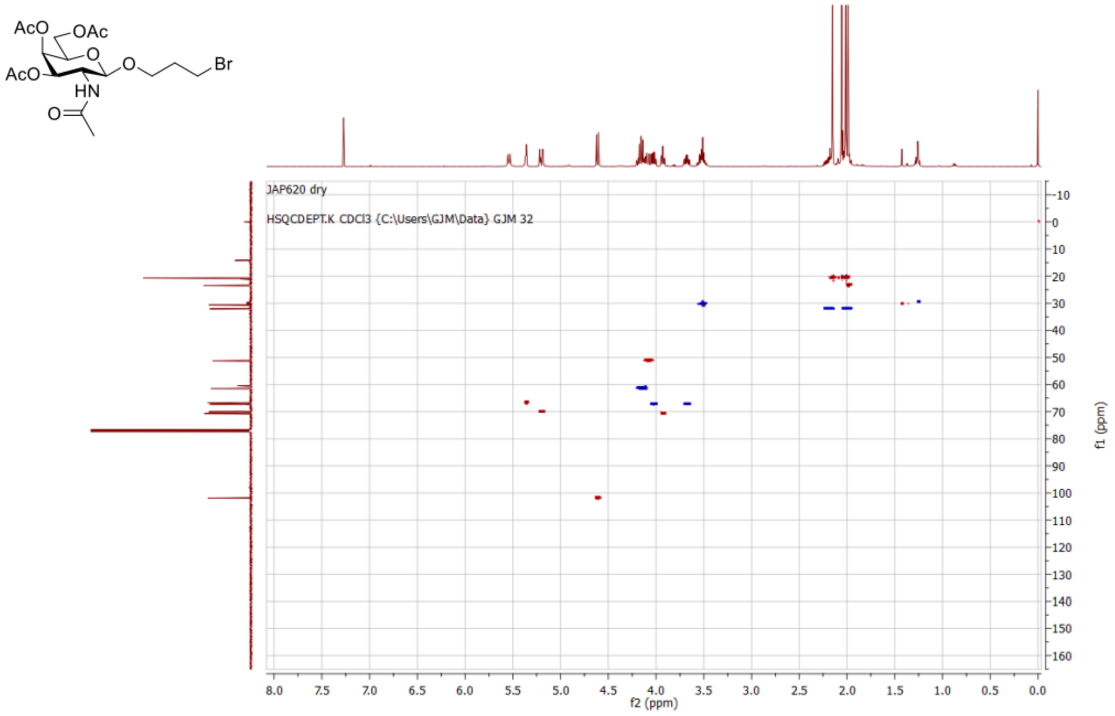
AcO
OAc
O
O
Br
AcO
HN
O
JAP620 dry
HSQCDEPT.K CDCl3 {C:\Users\GJM\Data} GJM 32
f2 (ppm)
f1 (ppm)

3-Azidopropyl (2-acetamido-3,4,6-tri-O-acetyl-2-deoxy)-β-D-galactopyranoside S5

^{1}H

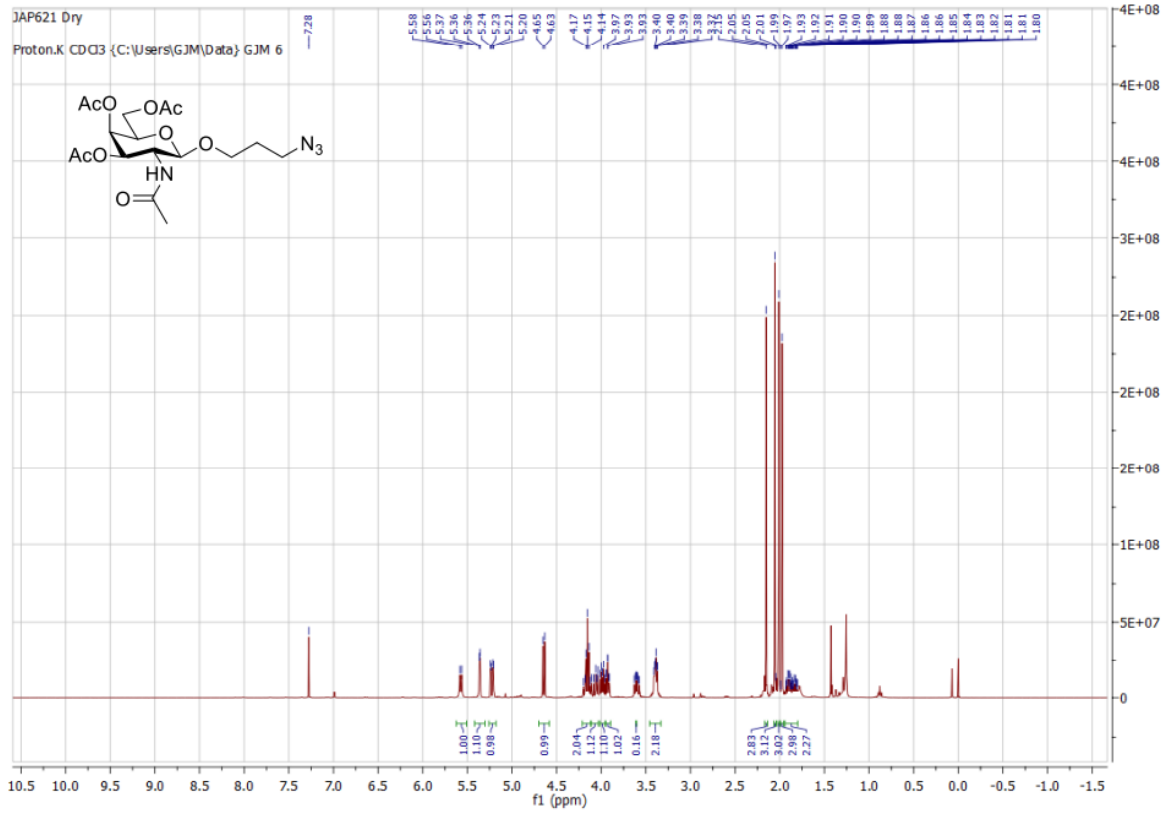

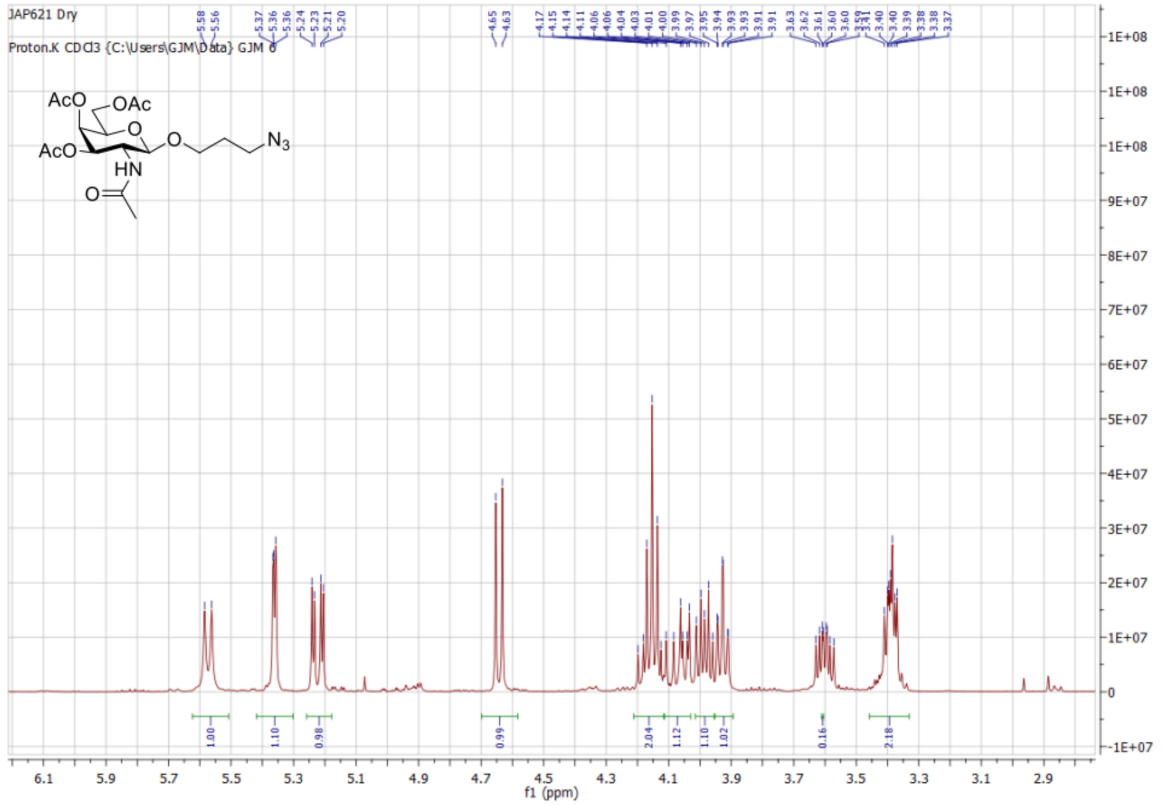

^{13}C

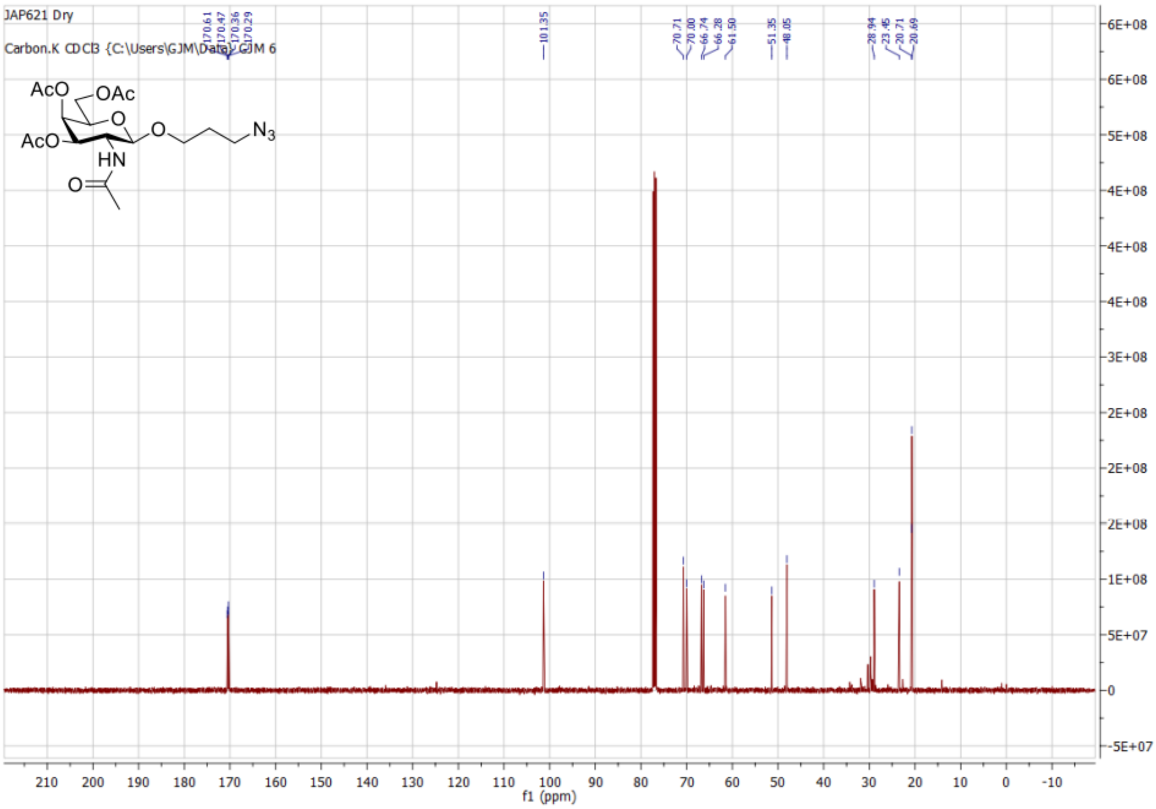
JAP621 Dry
Carbon.K CDCl3 {C:\Users\GJM\Data} GJM 6
170.61
170.47
170.36
170.29
101.35
70.71
70.00
66.74
66.28
61.50
51.35
48.05
28.94
23.45
20.71
20.69
AcO
OAc
AcO
O
O
N3
HN
O
f1 (ppm)

COSY

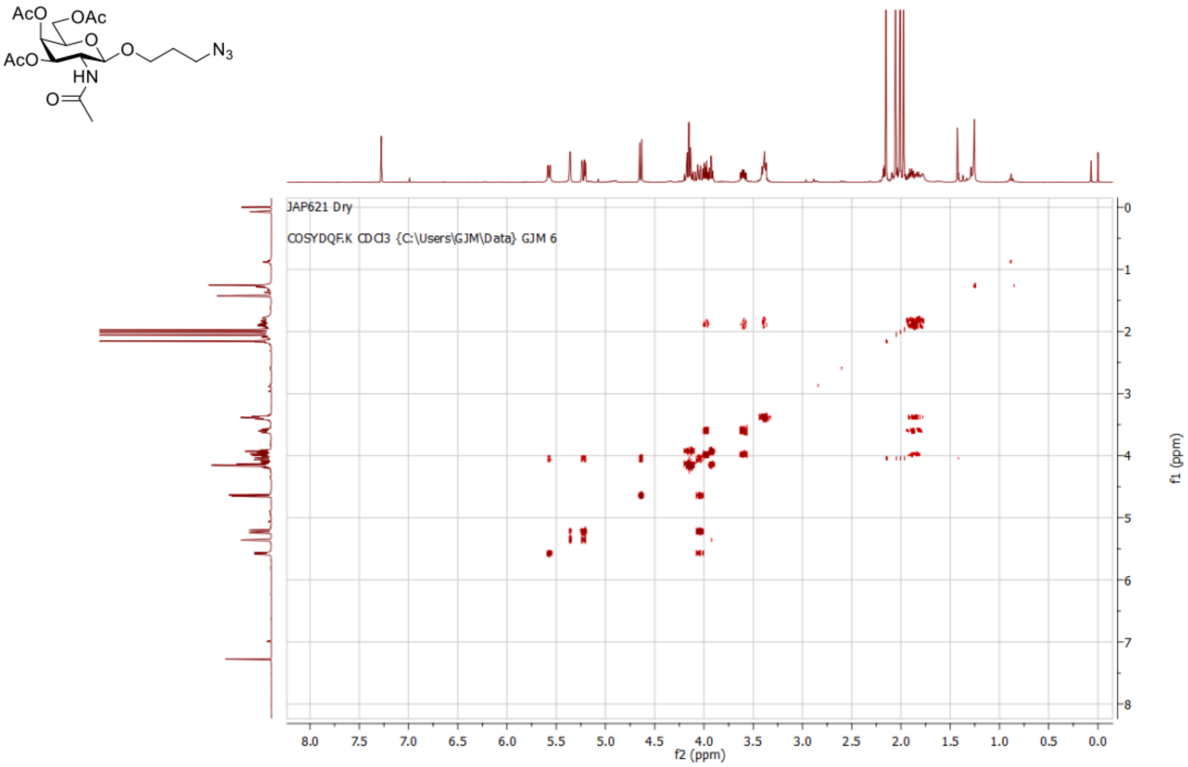
AcO
OAc
AcO
O
O
N3
HN
O
JAP621 Dry
COSYDQF.K CDCl3 {C:\Users\GJM\Data} GJM 6
f2 (ppm)
f1 (ppm)

HSQC

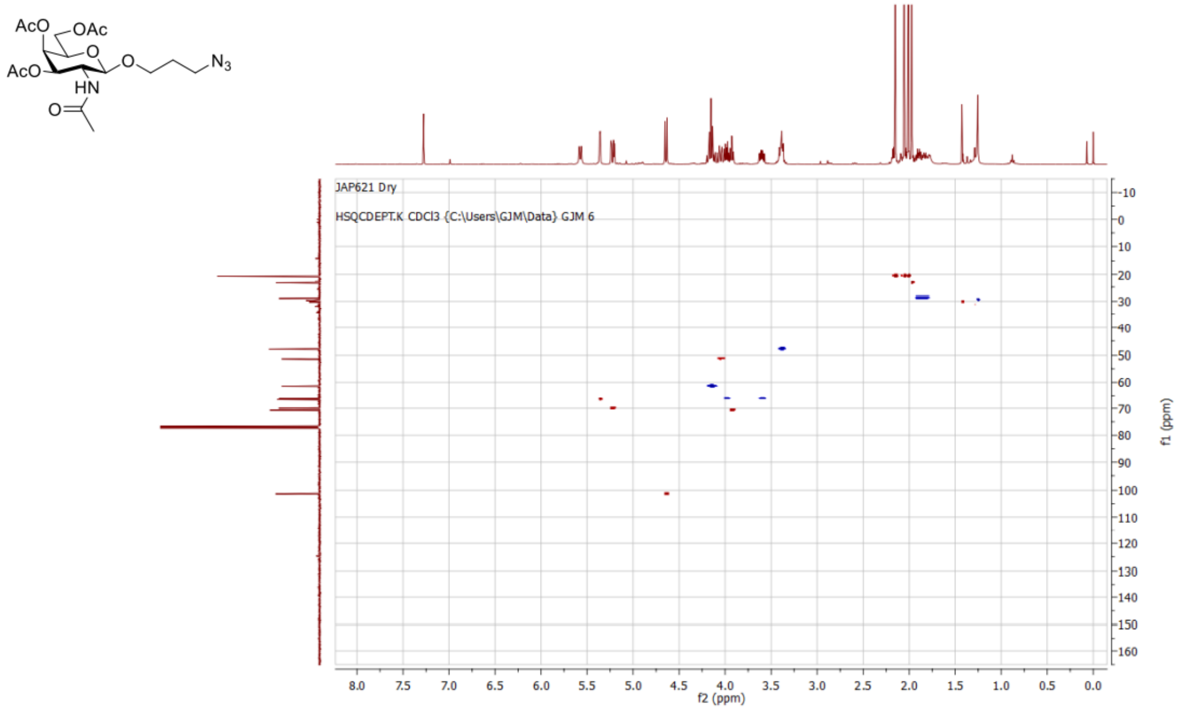

3-Azidopropyl (2-acetamido-3,6-di-O-benzoyl-2-deoxy)-β-D-galactopyranoside S6

^{1}H

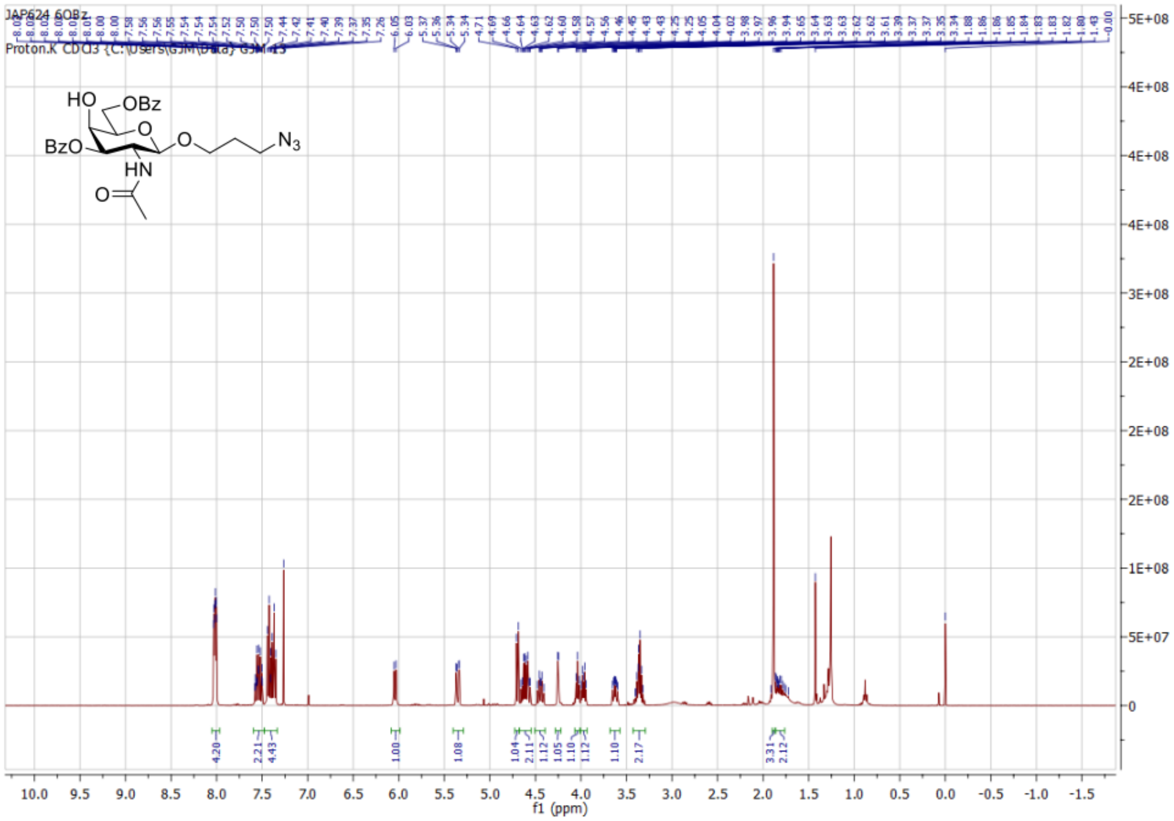

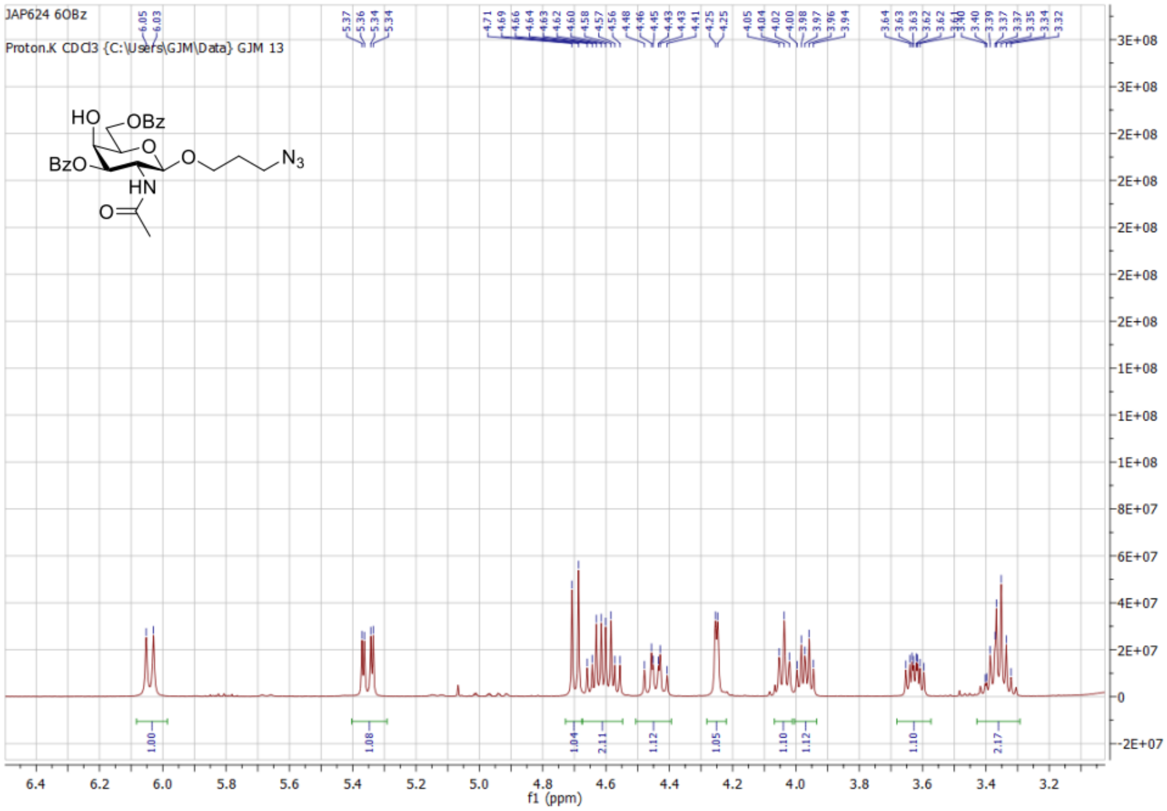

JAP624 6OBz
Proton.K CDCl3 {C:\Users\GJM\Data} GJM 13
HO
OBz
BzO
O
O
N_3
HN
O
f1 (ppm)

^{13}C

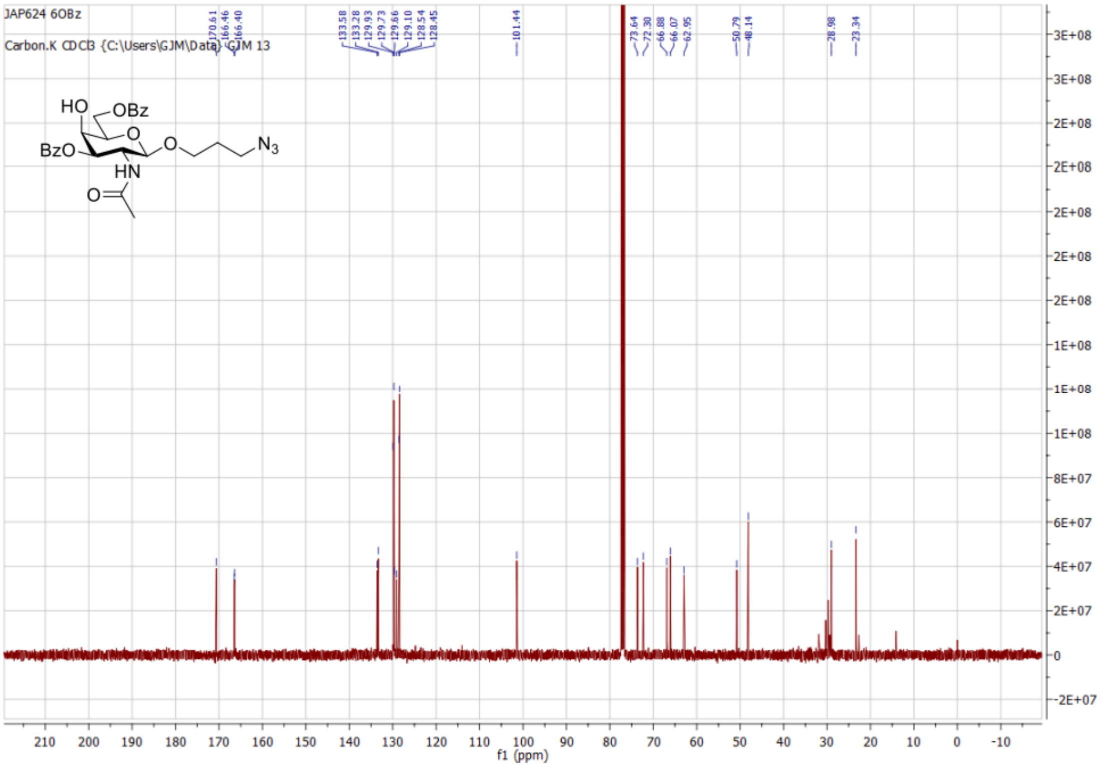

JAP624 6OBz
Carbon.K CDCl3 {C:\Users\GJM\Data} GJM 13
170.61
166.46
166.40
133.58
133.28
129.93
129.73
129.66
129.10
128.54
128.45
101.44
73.64
72.30
66.88
66.07
62.95
50.79
48.14
28.98
23.34
HO
OBz
BzO
O
O
N_3
HN
O
f1 (ppm)

COSY

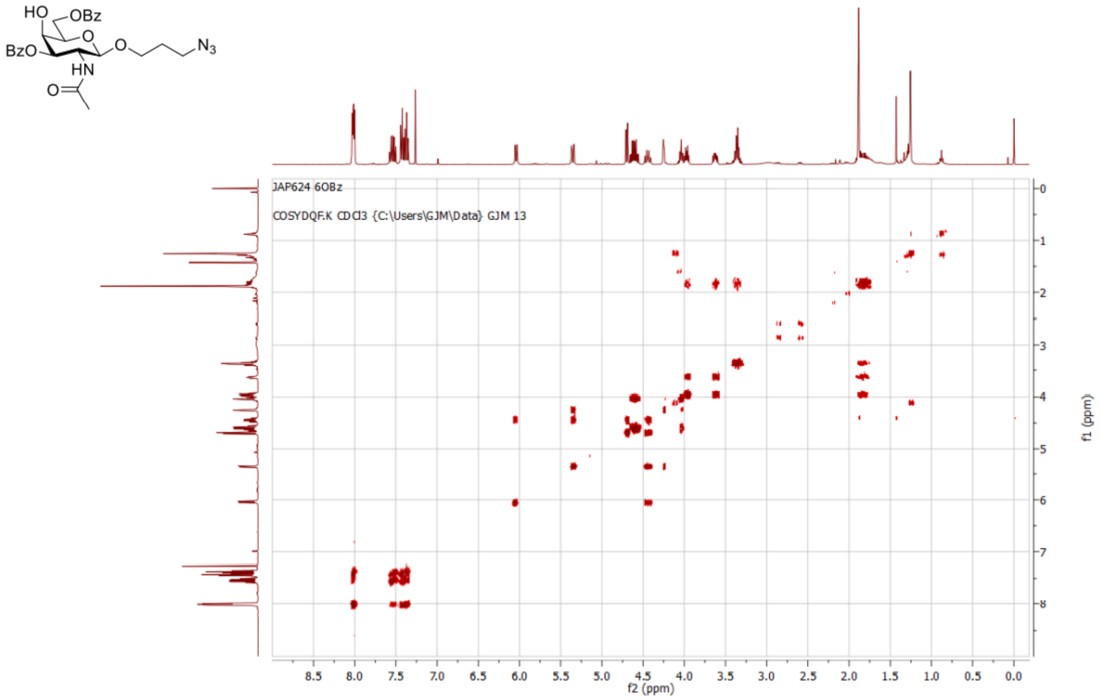
HO
OBz
BzO
O
HN
O
N_3
JAP624 6OBz
COSYDQF.K CDCl3 {C:\Users\GJM\Data} GJM 13
f2 (ppm)
f1 (ppm)

HSQC

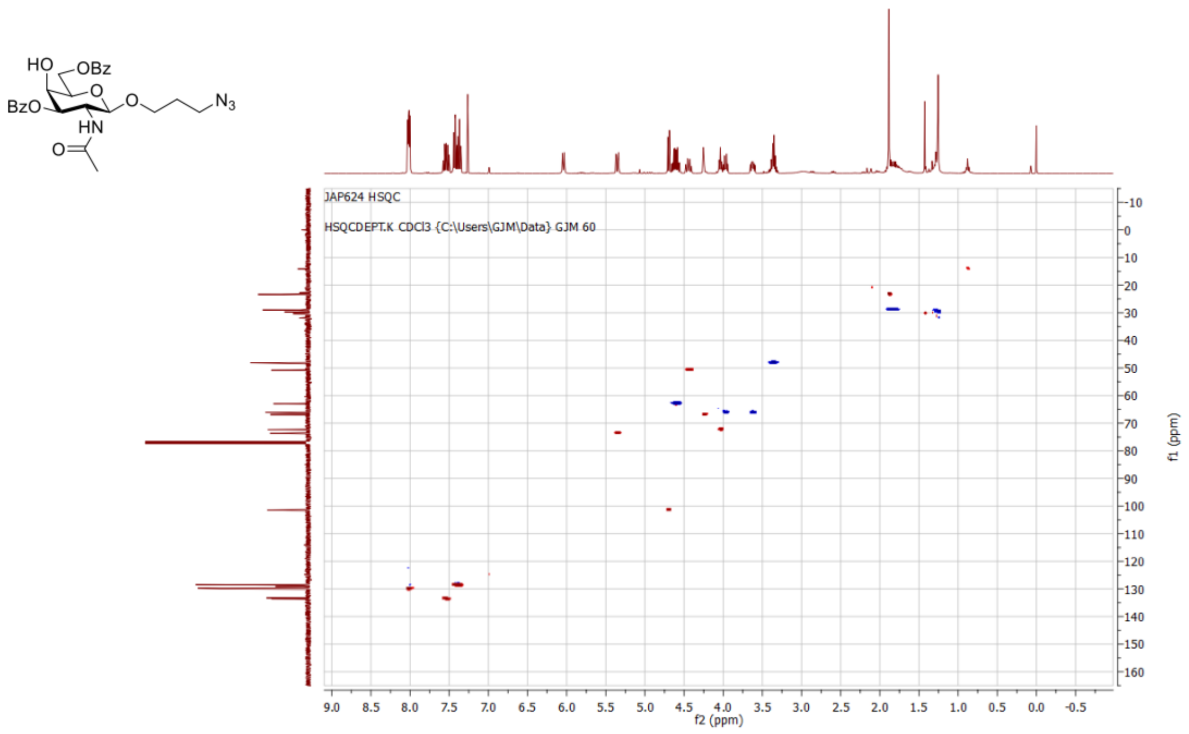
HO
OBz
BzO
O
HN
O
N_3
JAP624 HSQC
HSQCDEPT.K CDCl3 {C:\Users\GJM\Data} GJM 60
f2 (ppm)
f1 (ppm)

HMBC

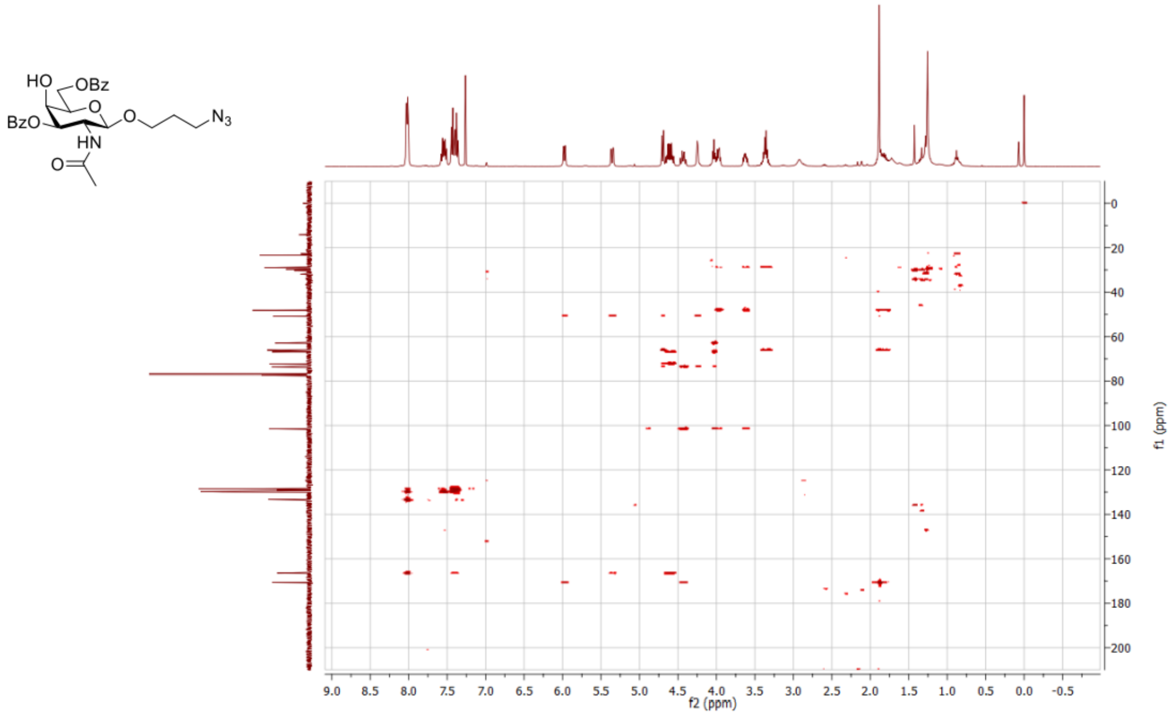

3-Azidopropyl (2-acetamido-4-S-acetyl-3,6-di-O-benzoyl-2-deoxy)-β-D-glucopyranoside S7

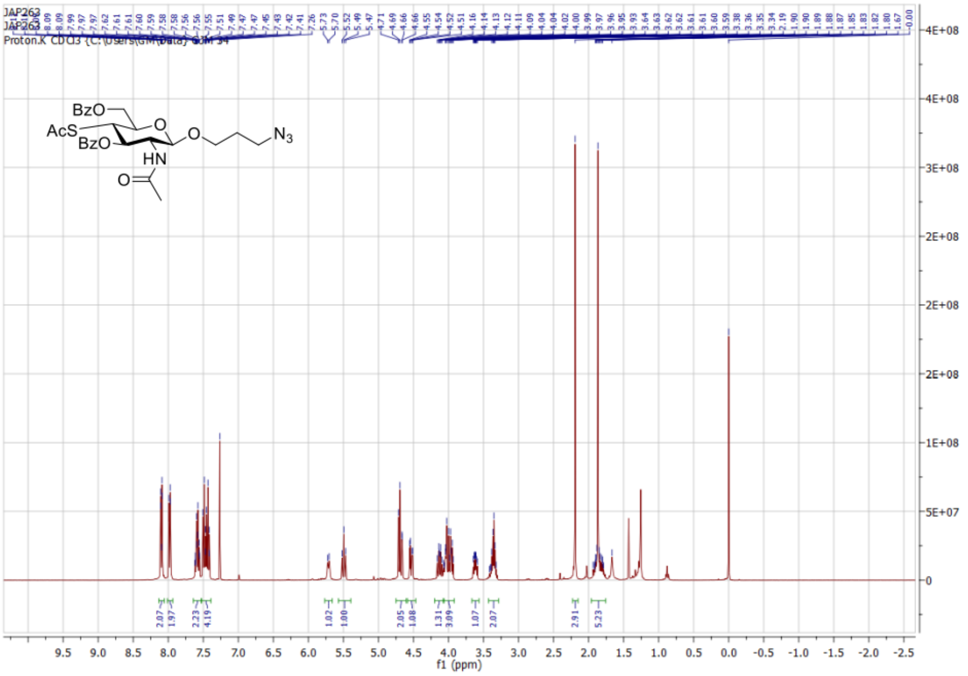

^{1}H

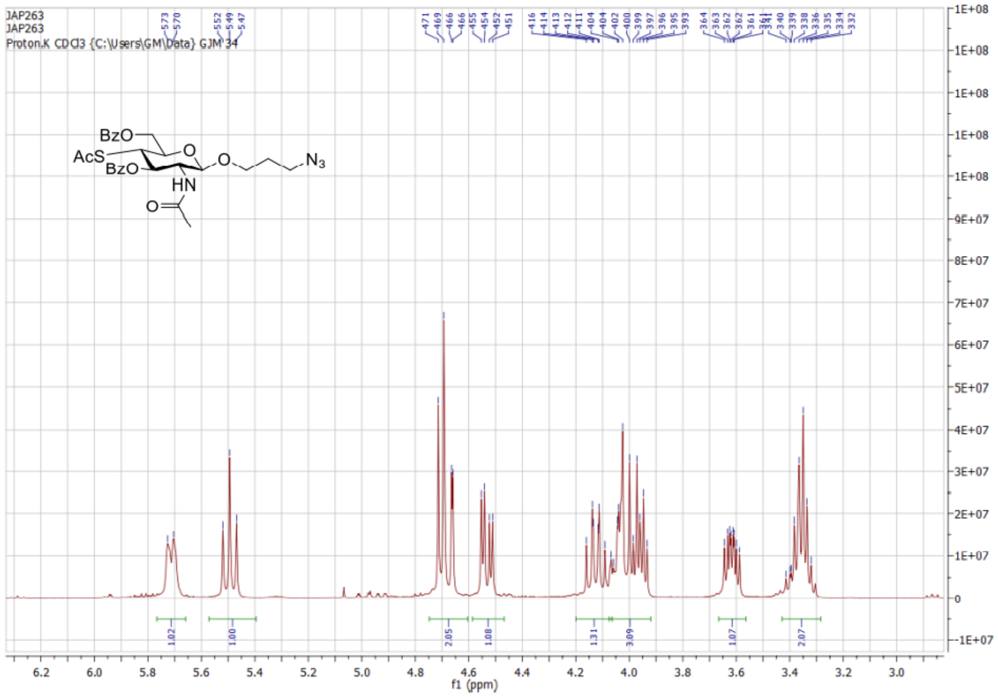
JAP263
JAP263
Proton.K CDCl3 {C:\Users\GM\Data} GJM 34
573
570
552
549
547
471
469
466
466
455
454
452
451
416
414
413
412
411
404
404
402
400
399
397
396
395
393
364
363
362
362
361
361
341
340
339
338
336
335
334
332
BzO
AcS
BzO
O
O
N_3
HN
O
1.02
1.00
2.05
1.08
1.31
3.09
1.07
2.07
f1 (ppm)

^{13}C

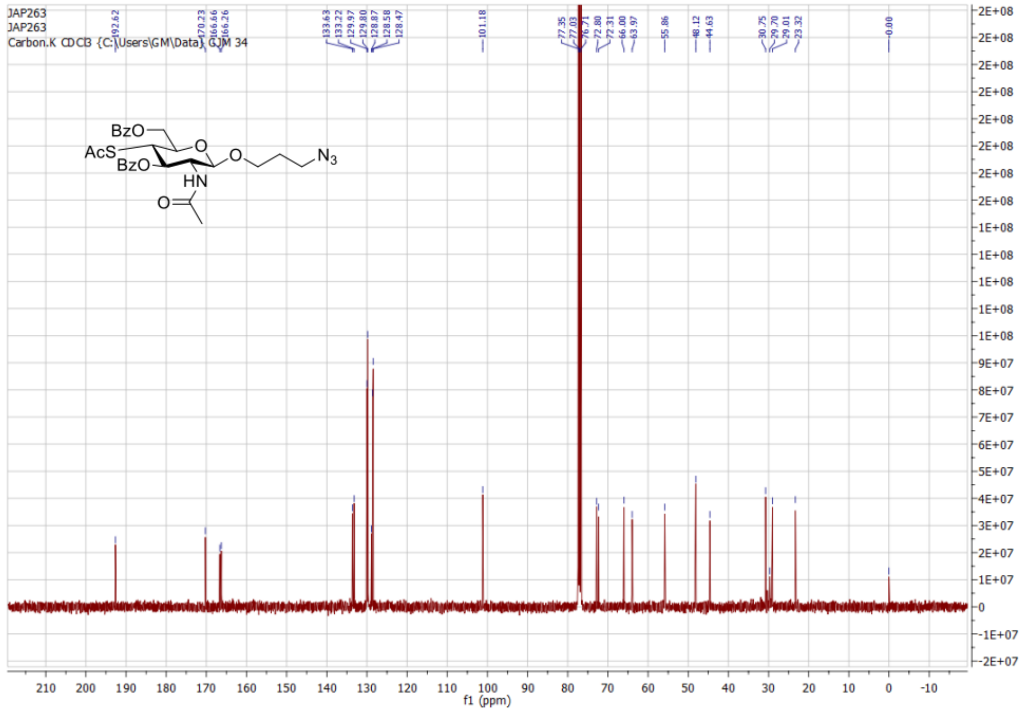
JAP263
JAP263
Carbon.K CDCl3 {C:\Users\GM\Data} GJM 34
192.62
170.23
166.66
166.26
133.63
133.22
129.97
129.80
128.87
128.58
128.47
101.18
77.35
77.03
76.71
72.80
72.31
66.00
63.97
55.86
48.12
44.63
30.75
29.70
29.01
23.32
0.00
BzO
AcS
BzO
O
O
N_3
HN
O
f1 (ppm)

COSY

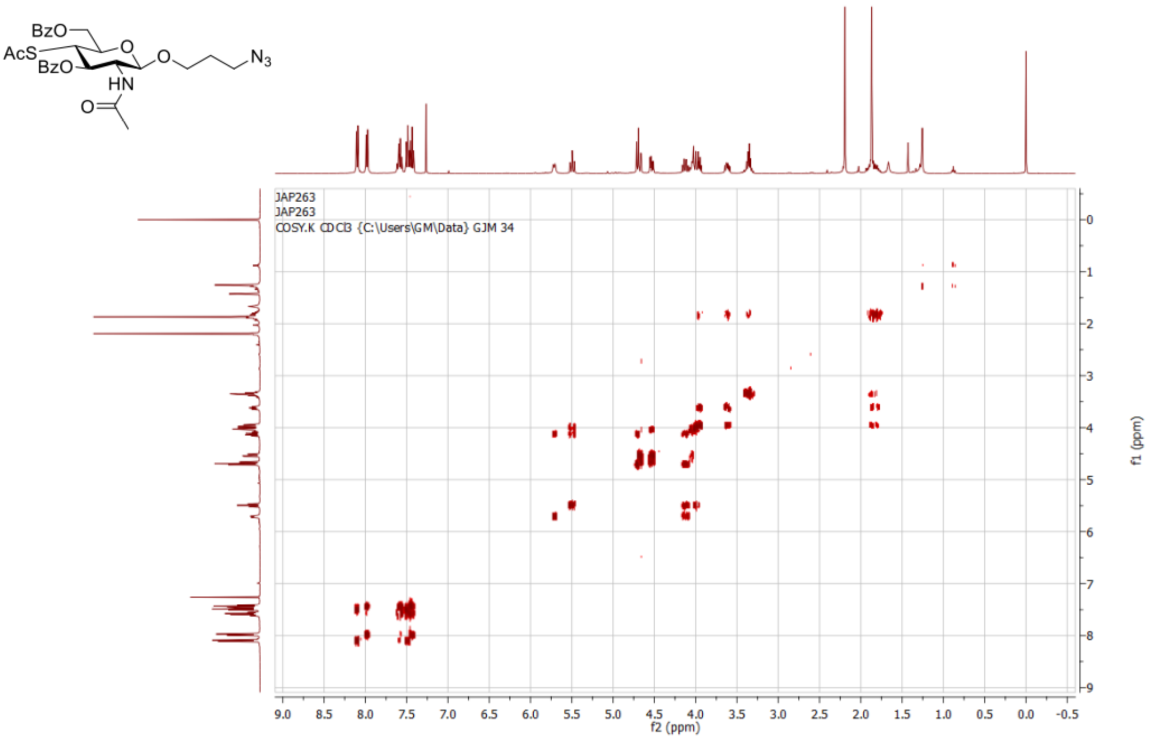
BzO
AcS
BzO
O
O
N3
HN
O
JAP263
JAP263
COSY.K CDCl3 {C:\Users\GM\Data} GJM 34
f2 (ppm)
f1 (ppm)

HSQC

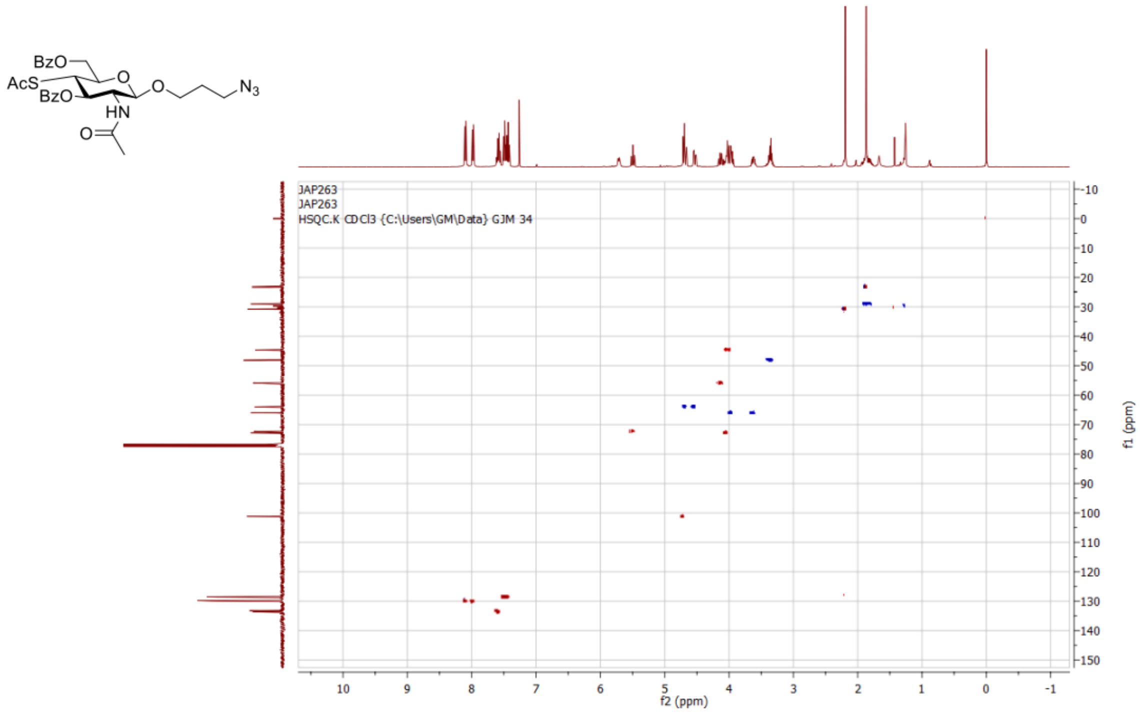
BzO
AcS
BzO
O
O
N3
HN
O
JAP263
JAP263
HSQC.K CDCl3 {C:\Users\GM\Data} GJM 34
f2 (ppm)
f1 (ppm)

Bis[3-azidopropyl (2-acetamido-2,4-dideoxy)-β-D-glucopyranos-4-yl] disulphide 33

^{1}H

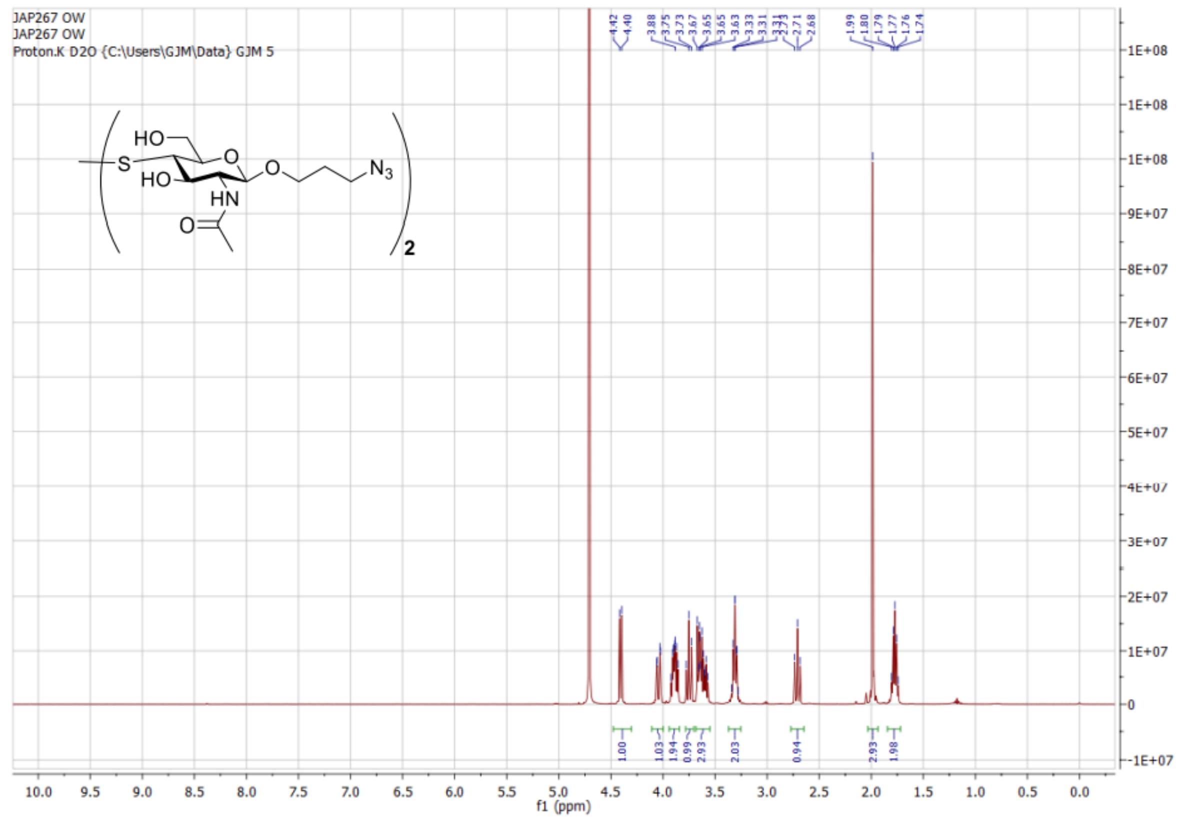

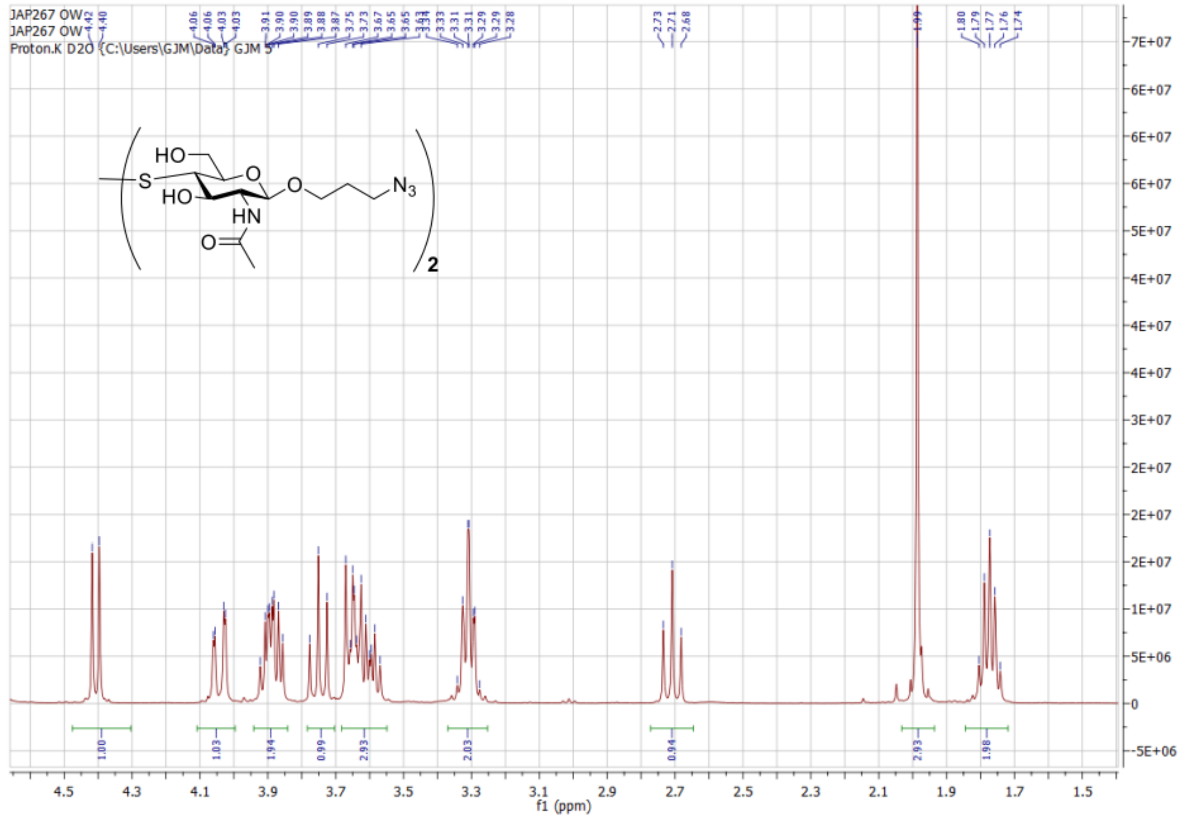

^{13}C

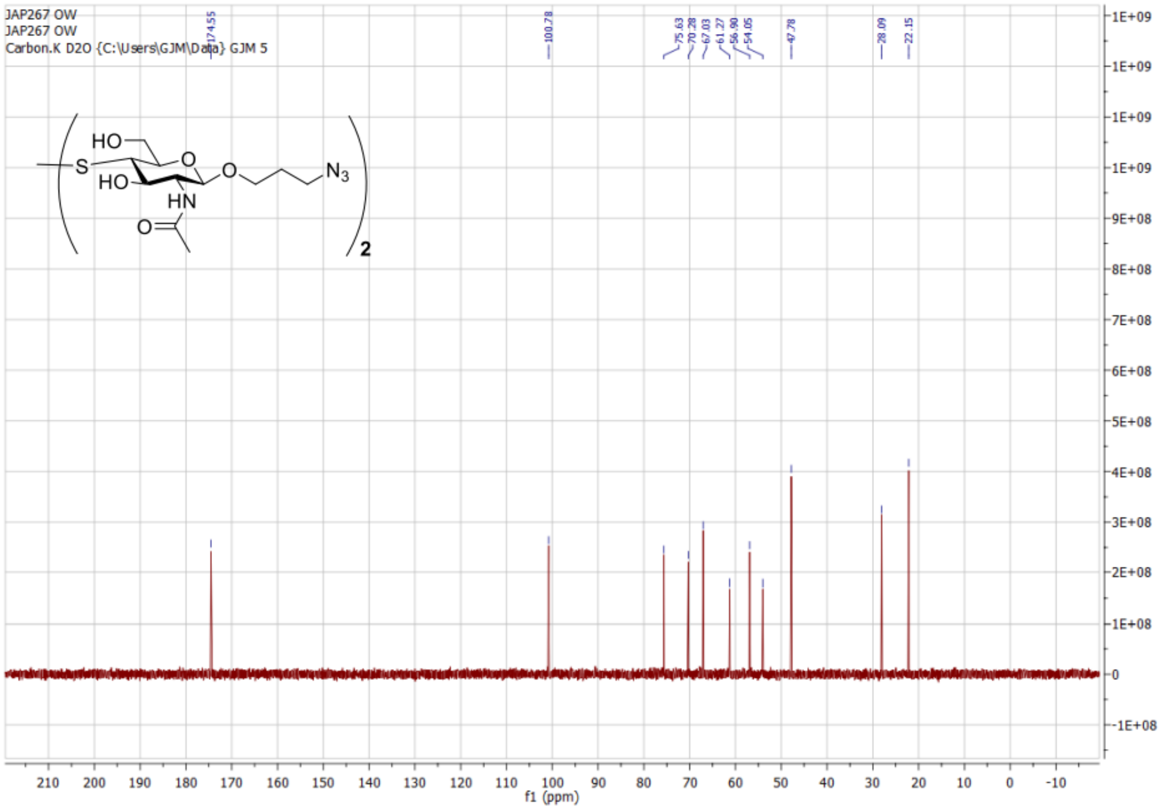
JAP267 OW
JAP267 OW
Carbon.K D2O {C:\Users\GJM\Data} GJM 5
174.55
100.78
75.63
70.28
67.03
61.27
56.90
54.05
47.78
28.09
22.15
f1 (ppm)

COSY

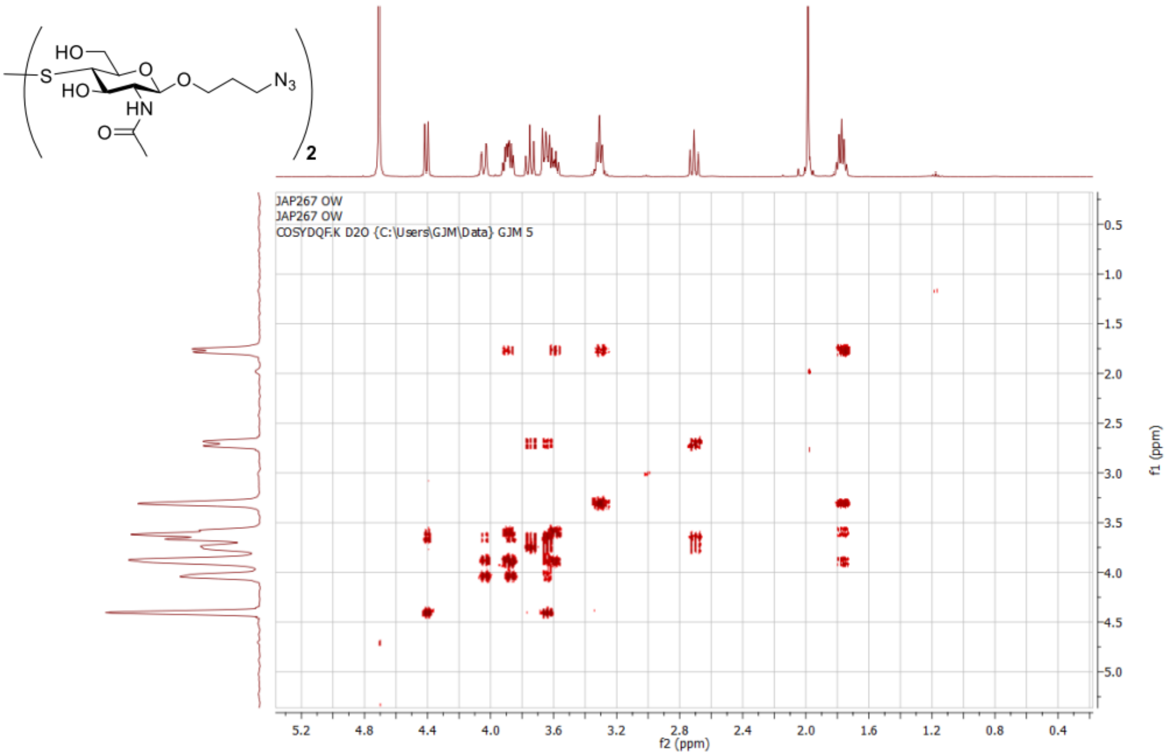
JAP267 OW
JAP267 OW
COSYDQF.K D2O {C:\Users\GJM\Data} GJM 5
f2 (ppm)
f1 (ppm)

HSQC

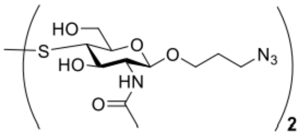

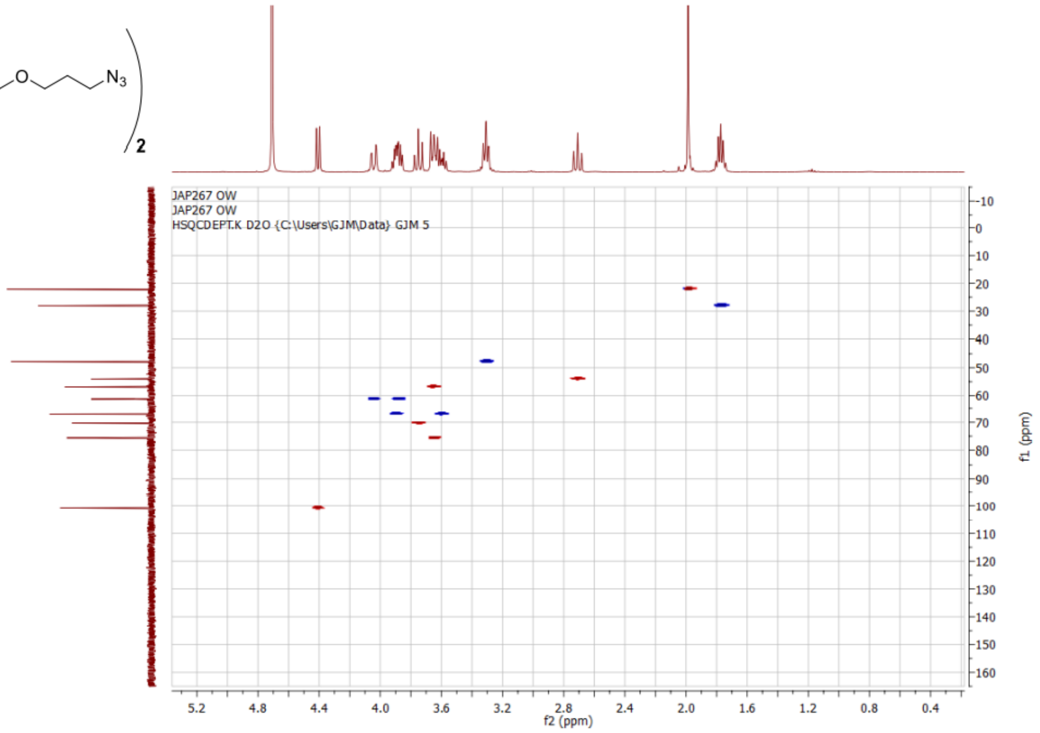

1-(4-(hydroxymethyl)benzyl)-3-methyl-imidazolium trifluoromethanesulfonate S8.

^{1}H

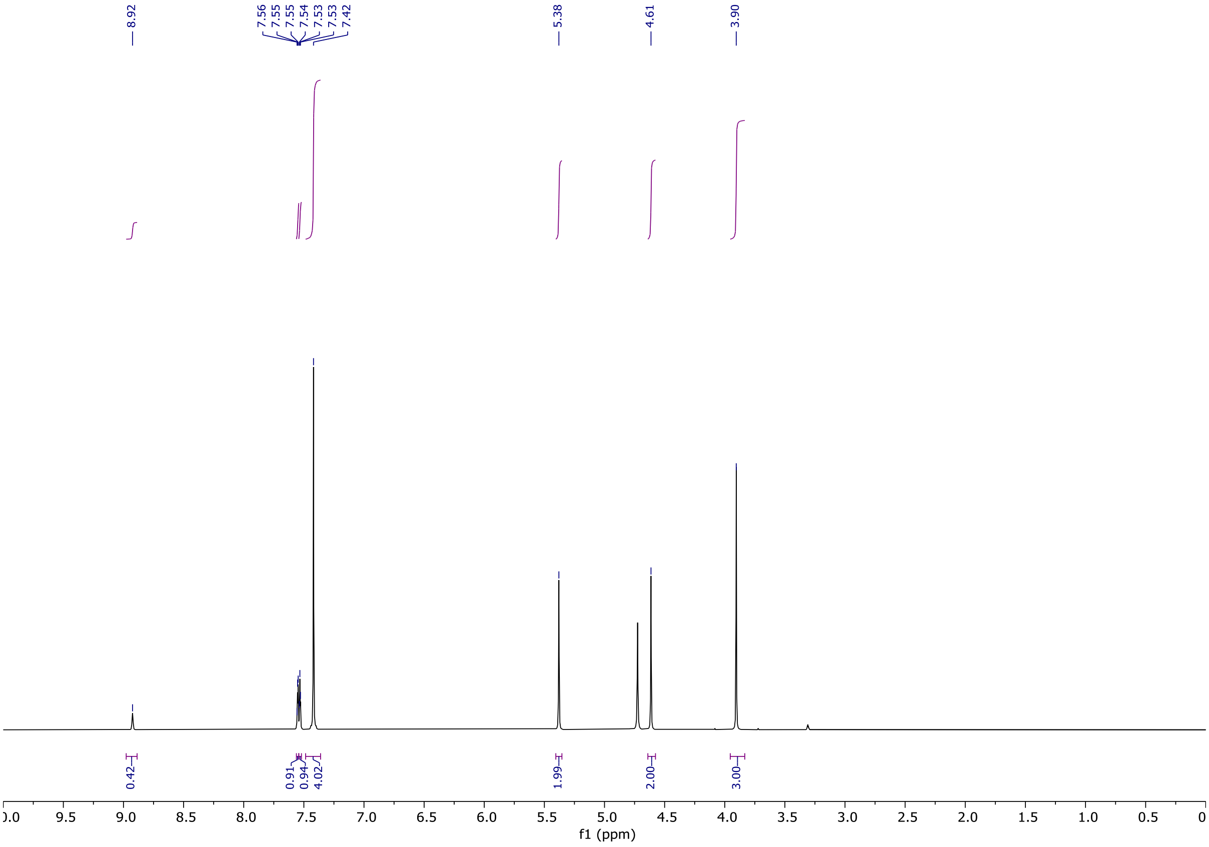

^{13}C

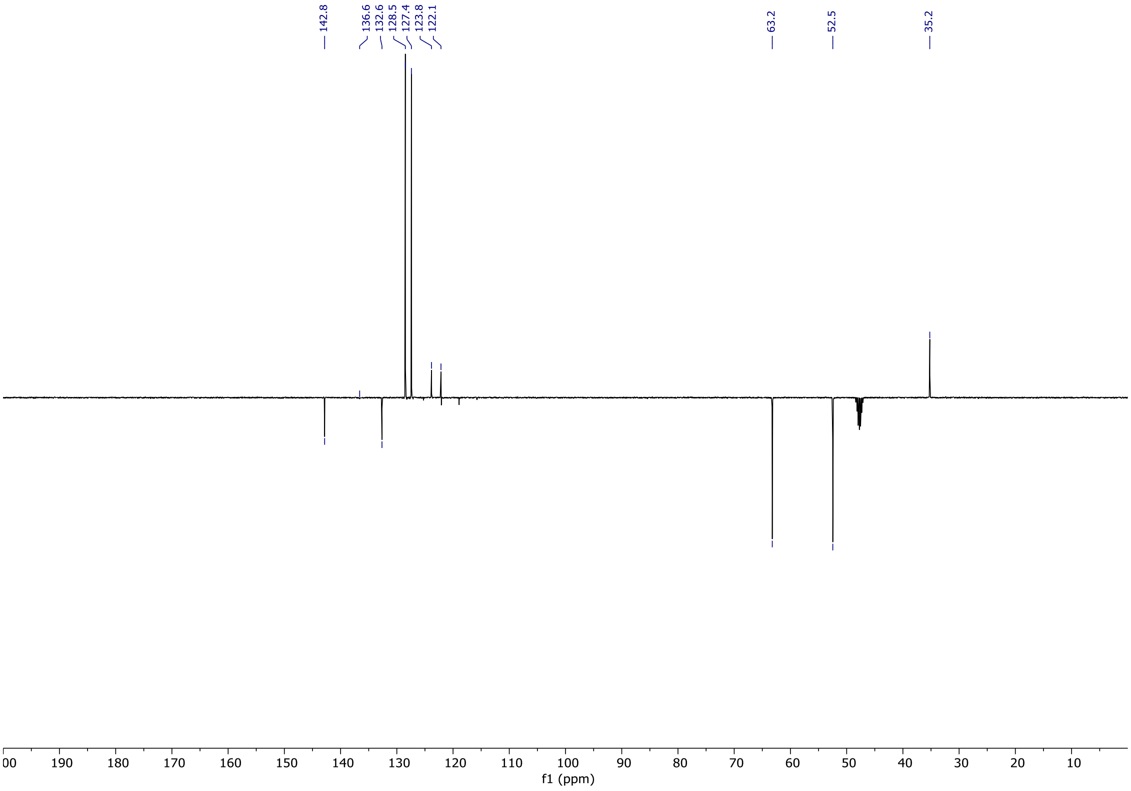

1-(4-((((4-nitrophenoxy)carbonyl)oxy)methyl)benzyl-3-methyl-imidazolium trifluoromethanesulfonate. S9

^{1}H

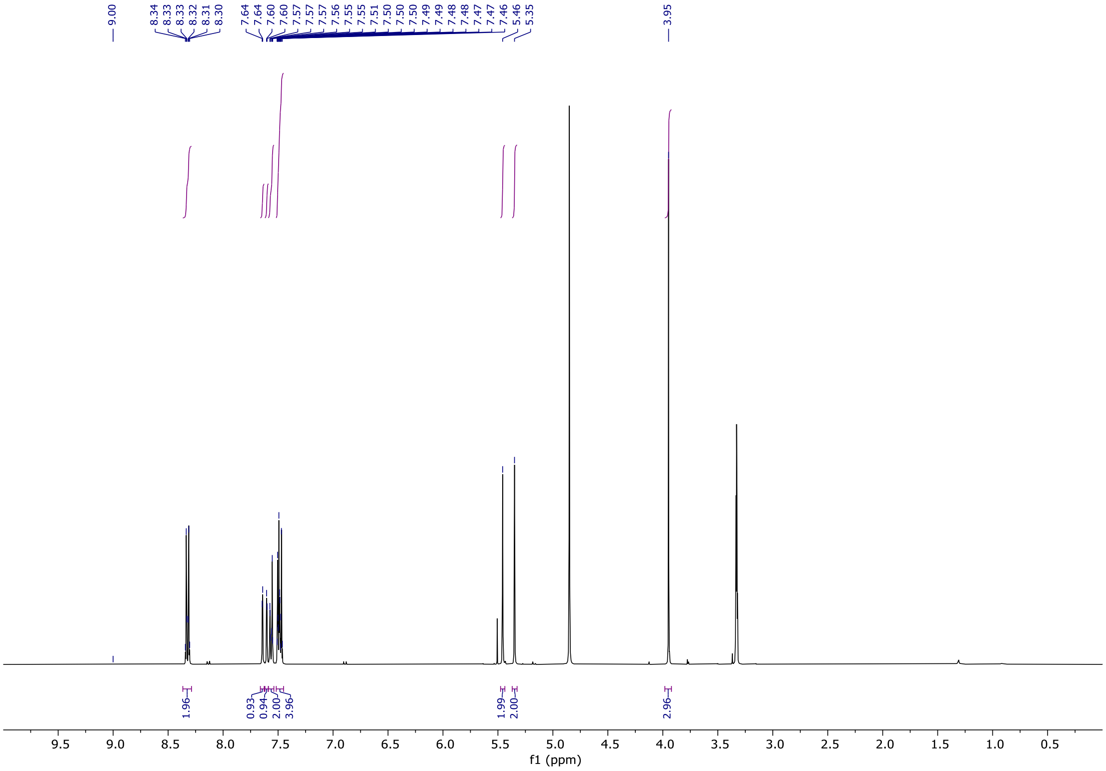

^{13}C

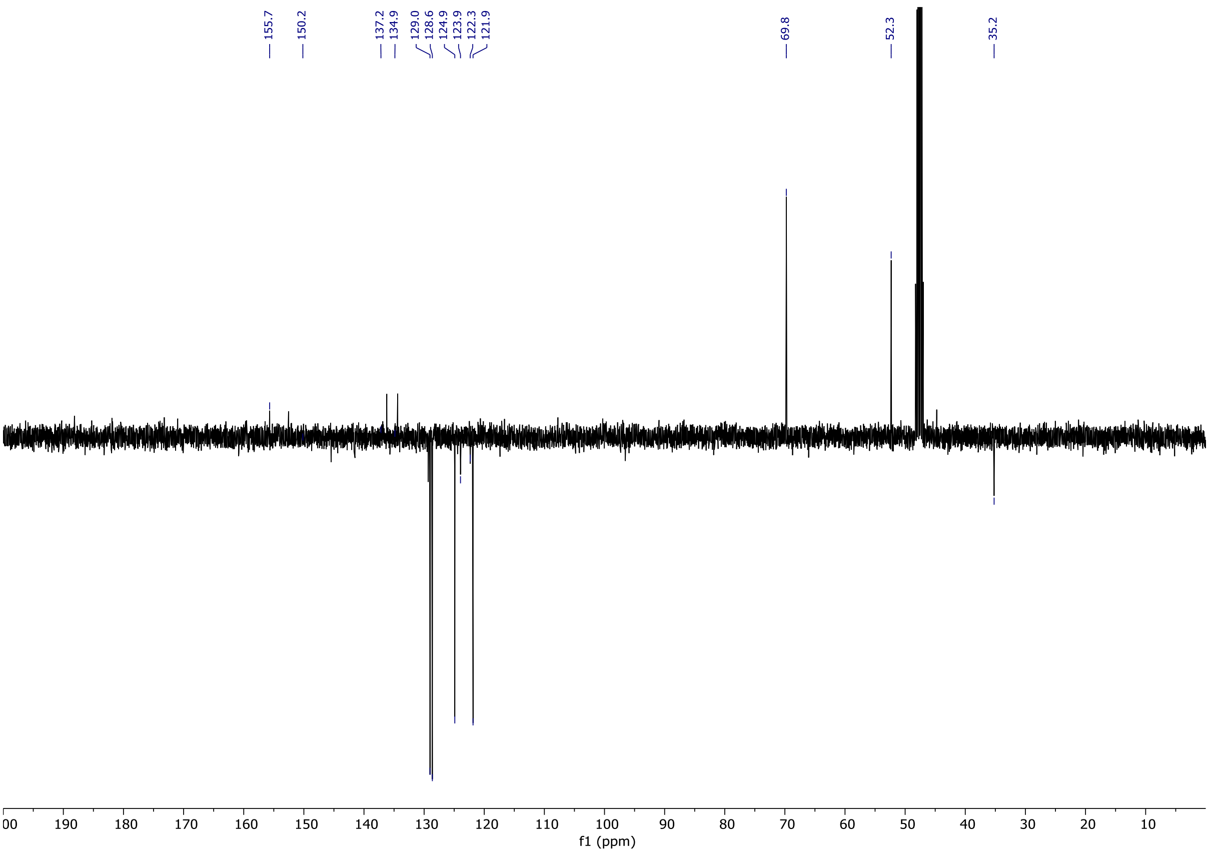

1-(4-(((but-3-yn-1-ylcarbamoyl)oxy)methyl)benzyl)-3-methyl-imidazolium trifluoromethanesulfonate 1.

^{1}H

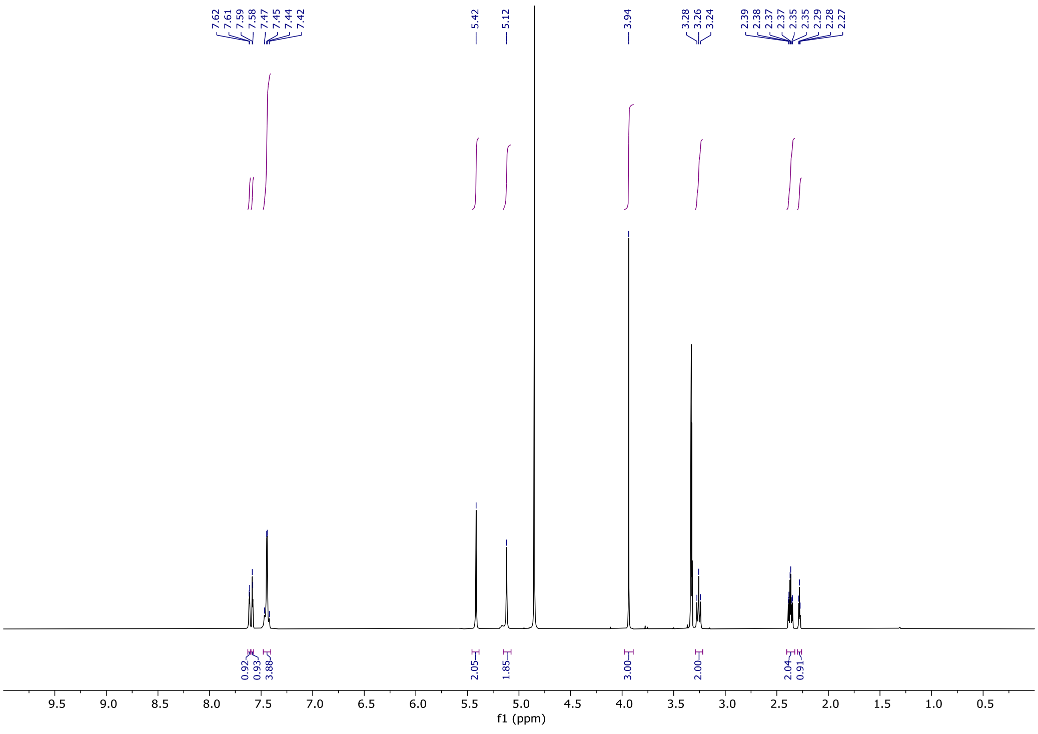

^{13}C

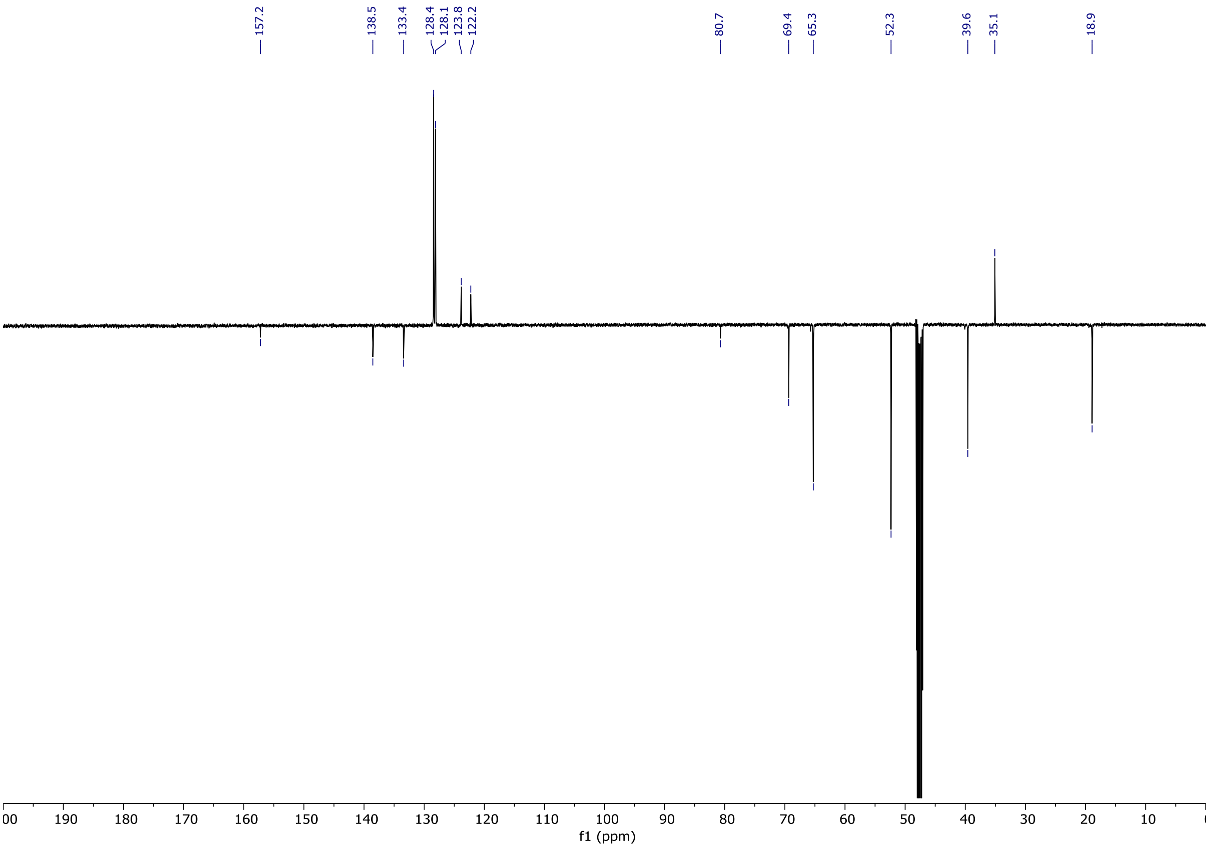

157.2
138.5
133.4
128.4
128.1
123.8
122.2
80.7
69.4
65.3
52.3
39.6
35.1
18.9
f1 (ppm)

10. Determination of BT1033 specific activity

For the acceptor standard curves, stock solutions of 5 mM acceptor (11 or 33) were prepared in HPLC-grade H_2O. The stock solution for SH-GlcNAc-N_3 33, also contained TCEP reducing agent (2.5 mM). A two-fold serial dilution of the respective stock solutions was performed to produce a series of standards ranging from 5 mM to 0.15625 mM, for each acceptor. The standards were prepared in triplicate. 10 µL of each standard was incubated at 37 °C for 30 min and then lyophilised. Lyophilised samples were resuspended in 24 µL of H_2O, then 0.5 µL of Itag solution (250 mM in DMSO) was added to each sample. Then 10 µL of a stock solution containing 500 mM $CuSO_4$. 5 H_2O and 500 mM THPTA in H_2O, was added to each sample. Samples were mixed by vortexing and then 5 µL of a 1 M sodium ascorbate solution was added to each sample. Samples were further mixed by vortexing and then incubated for 2 h at 20 °C 300 rpm. 1 mL of H_2O was added to each sample and the samples were lyophilised. Samples were resuspended in 100 µL of H_2O. and analysed by C_{18}-LC-MS in positive-ion mode. Spectra were processed in Compass 1.3 DataAnalysis, Version 4.1 software (Bruker Daltonics). The peak area for each standard was calculated from the extracted ion chromatogram (EIC) of the ITag-labelled acceptor (SH-GlcNAc-N_3 $[M+H]^+$- *m/z* 618 or GlcNAc-N_3 $[M+H]^+$ - *m/z* 602). The mean peak area was then plotted as a function of acceptor concentration to produce a calibration curve (Figure S34).

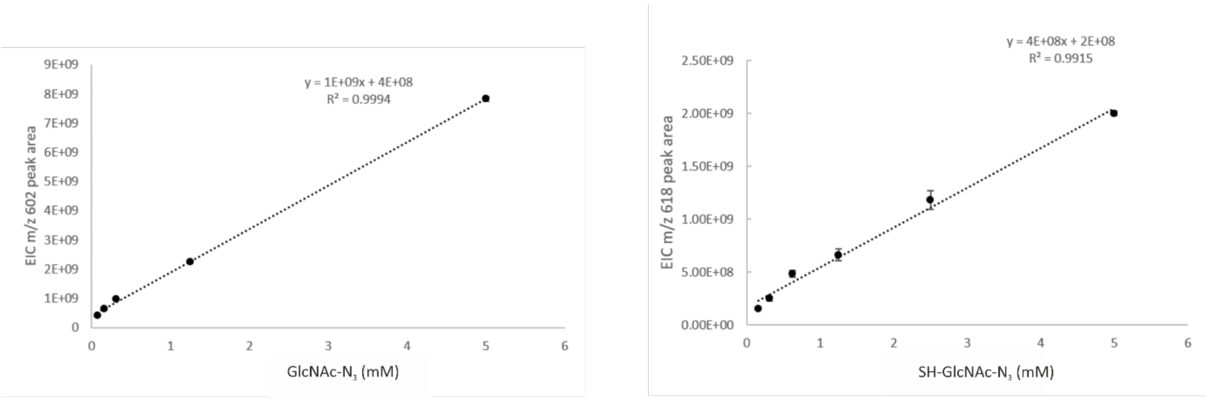

Figure S34. Standard curve of GlcNAc-N3 (A) or SH-GlcNAc-N3 (B) concentration plotted against the peak area for the extracted ion chromatogram (EIC) for m/z 602 or m/z 618 respectively. Error bars denote standard error of the mean of 3 replicates.

To investigate specific activity, a series of reactions was assembled on a 10 µL scale containing 10 mM Man-1P 2, 3 mM acceptor 11 or 33, 10 mM $MgCl_2$, 40 mM NaOAc buffer pH 5.5 and 1 µL of BT1033 (stock concentrations ranging from 13 mg/mL – 0.025 mg/mL). For reactions containing SH-GlcNAc-N_3 33, TCEP reducing agent was added to a final concentration of 1.5 mM. Reactions were incubated at 37 °C for 30 min. To stop the reaction, 10 µL of ice cold EtOH was added and the samples were

incubated at -20 °C. Samples were then lyophilised, following the addition of 1 mL of HPLC-grade H_2O. Lyophilised samples were resuspended in 24 µL of H_2O, then 0.5 µL of Itag solution (250 mM in DMSO) was added to each sample. Then 10 µL of a stock solution containing 500 mM $CuSO_4$. 5 H_2O and 500 mM THPTA in H_2O, was added to each sample. Samples were mixed by vortexing and then 5 µL of a 1 M sodium ascorbate solution was added to each sample. Samples were further mixed by vortexing and then incubated for 2 h at 20 °C 300 rpm. 1 mL of H_2O was added to each sample and the samples were lyophilised. Samples were resuspended in 100 µL of H_2O. and analysed by C_{18}-LC-MS in positive-ion mode. In all of the reactions, the molecular ion of the respective ITag-labelled acceptor (SH-GlcNAc-N_3 $[M+H]^+$- *m/z* 618 or GlcNAc-N_3 $[M+H]^+$ - *m/z* 602) was monitored in order to quantify reaction progress. The peak area for the acceptor was calculated in each reaction from the extracted ion chromatogram (EIC) of the ITag-labelled acceptor (SH-GlcNAc-N_3 - *m/z* 618 or GlcNAc-N_3 - *m/z* 602) and the absolute concentration of acceptor was obtained from the standard curve (Figure S34). Conversion (%) to product was calculated as follows:

$$\frac{\text{Starting [acceptor] mM} - \text{final [acceptor] mM}}{\text{Starting [acceptor] mM}} \quad \text{X} \quad 100$$

Conversion (%) was plotted against enzyme concentration (Figure S35). Specific activity (Table S5) was calculated at the enzyme concentration that resulted in ~ 15% conversion (i.e. the initial change in conversion).

Table S5 Specific activity of BT1033 to GlcNAc-N_3 11 and SH-GlcNAc-N_3 33.

**Acceptor**	**Specific activity (µmol. min^{-1}. mg^{-1})**
GlcNAc-N_3	3.20
SH-GlcNAc-N_3	1.59

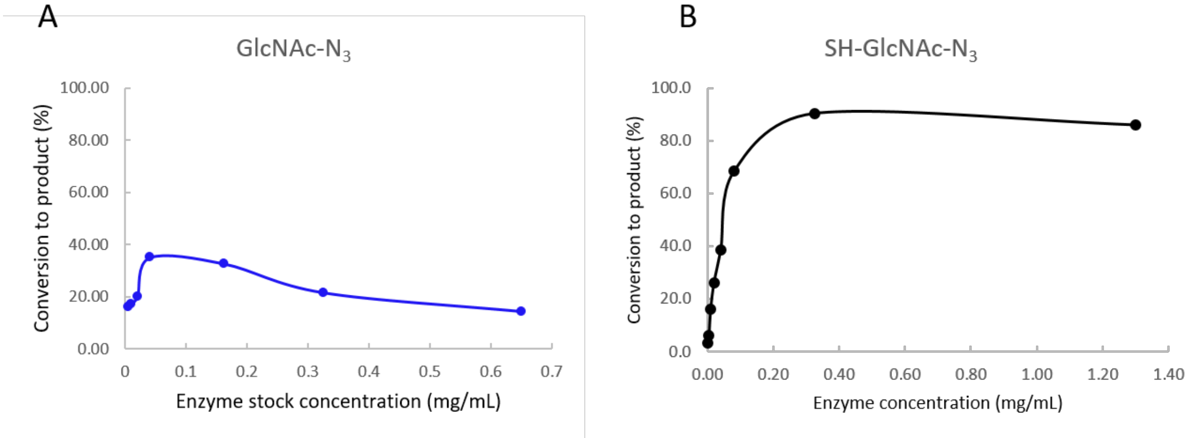

Figure S35. BT1033 titration curve for reactions with GlcNAc-N_3 (A) or SH-GlcNAc-N_3 (B).

11. Molecular modelling

The BT1033 amino acid sequence was obtained from UniProt and the PDB file was obtained from the AlphaFold Protein Structure Database (UniProt entry: Q8A8Y4). Uhgb_MP amino acid sequence and PDB file was obtained from RCSB Protein Data Bank (PDB) (entry 4UDJ). Molecular graphics and analyses were performed with UCSF Chimera.[9] NCBI BLAST web interface (https://blast.ncbi.nlm.nih.gov/Blast.cgi) was used for sequence alignment of BT1033 to Uhgb_MP (Figure S36).

Range 1 : 5 to 327

Score	Expect	Method	Identities	Positives	Gaps
454:bits (1167)	2E-166	Compositional matrix adjust	214/323 (66%)	255/323 (78%)	2/323 (0%)

```
BT1033    2 NKIQIPWEERPVGCTDVMWRYSQNPVIGRYHIPSSNSIFNSAVVPFKDGFAGVFRCDNKA 61
            +K+ IPWEERP GC DV+WR   NP+I R  +P+SNSIFNSAVVPF DGFAGVFRCD+ +
Uhgb_MP   5 SKVIIPWEERPAGCKDVLWRSVANPIIPRDLLPTSNSIFNSAVVPFGDGFAGVFRCDDTS 64

BT1033   62 VQMNIFTGFSKDGIHWDISHEPIQFKAGNTEMIESEYKYDPRVTWIEDRYWVTWCNGYHG 121
             +M +  GFSKD I+W+I  EP++F+  + E+    Y YDPRV +IEDRY+VTWCNGYHG
Uhgb_MP  65 RRMRLHVGFSKDAINWNIKEEPLKFQCDDEEIGTWVYGYDPRVCFIEDRYYVTWCNGYHG 124

BT1033  122 PTIGIAYTFDFVDFFQCENAFLPFNRNGVLFPQKIDGKYAMLSRPSDNGHTPFGDIYISY 181
            PTIG+AYTFDF  F Q ENAF+PFNRNGVLFP+KI+G++AMLSRPSDNGHTPFGDI+ S 
Uhgb_MP 125 PTIGVAYTFDFETFHQLENAFIPFNRNGVLFPRKINGRFAMLSRPSDNGHTPFGDIFYSE 184

BT1033  182 SPDMKYWGEHRCVMKVTPFPESAWQCTKIGAGSVPFLTDEGWLLFYHGVITTCNGFRYAM 241
            SPDM++WG HR VM    F  SAWQCTKIGAG +P  T E GWLL YHGV+ +CNG+ Y+ 
Uhgb_MP 185 SPDMEFWGRHRHVMSPAAFEVSAWQCTKIGAGPIPVETPEGWLLIYHGVLHSCNGYVYSF 244

BT1033  242 GSAILDKDHPEKVLYRTREYLIGPAAPYELQGDVPNVVFPCAALQDGE--RVAVYYGAAD 299
            GSA+LD D P KV +R+  YL+ P  PYE  GDVPNV FPCAAL D E   R+A+YYG AD
Uhgb_MP 245 GSALLDLDEPWKVKFRSGPYLLAPREPYECMGDVPNVCFPCAALHDNETGRIAIYYGCAD 304

BT1033  300 TVVGMAFGYIQEIIDFTKRTSII 322
            TV G+AFGYI EII+FTKRTSII
Uhgb_MP 305 TVTGLAFGYIPEIIEFTKRTSII 327
```

Figure S36. An amino acid sequence alignment of BT1033 and Uhgb_MP. The conserved catalytic Asp is highlighted in turquoise.

12. References

(1) Beswick, L.; Ahmadipour, S.; Hofman, G.-J.; Wootton, H.; Dimitriou, E.; Reynisson, J.; Field, R. A.; Linclau, B.; Miller, G. J. Exploring anomeric glycosylation of phosphoric acid: Optimisation and scope for non-native substrates. *Carbohydrate Research* **2020**, *488*.

(2) Ahmadipour, S.; Wahart, A. J.; Dolan, J. P.; Beswick, L.; Hawes, C. S.; Field, R. A.; Miller, G. J. Synthesis of C6-modified mannose 1-phosphates and evaluation of derived sugar nucleotides against GDP-mannose dehydrogenase. *Beilstein Journal of Organic Chemistry* **2022**, *18* (1), 1379-1384.

(3) Beswick, L.; Dimitriou, E.; Ahmadipour, S.; Zafar, A.; Rejzek, M.; Reynisson, J.; Field, R. A.; Miller, G. J. Inhibition of the GDP-d-Mannose Dehydrogenase from Pseudomonas aeruginosa Using Targeted Sugar Nucleotide Probes. *ACS Chemical Biology* **2020**, *15* (12), 3086-3092.

(4) Ahmadipour, S.; Pergolizzi, G.; Rejzek, M.; Field, R. A.; Miller, G. J. Chemoenzymatic Synthesis of C6-Modified Sugar Nucleotides to Probe the GDP-D-Mannose Dehydrogenase from Pseudomonas aeruginosa. *Organic letters* **2019**, *21* (12), 4415-4419.

(5) Richards, S.-J.; Keenan, T.; Vendeville, J.-B.; Wheatley, D. E.; Chidwick, H.; Budhadev, D.; Council, C. E.; Webster, C. S.; Ledru, H.; Baker, A. N. Introducing affinity and selectivity into galectin-targeting nanoparticles with fluorinated glycan ligands. *Chemical science* **2021**, *12* (3), 905-910.

(6) Dowlut, M.; Hall, D. G.; Hindsgaul, O. Investigation of nonspecific effects of different dyes in the screening of labeled carbohydrates against immobilized proteins. *The Journal of organic chemistry* **2005**, *70* (24), 9809-9813.

(7) Bernardes, G. J.; Kikkeri, R.; Maglinao, M.; Laurino, P.; Collot, M.; Hong, S. Y.; Lepenies, B.; Seeberger, P. H. Design, synthesis and biological evaluation of carbohydrate-functionalized cyclodextrins and liposomes for hepatocyte-specific targeting. *Organic & biomolecular chemistry* **2010**, *8* (21), 4987-4996.

(8) Nihira, T.; Suzuki, E.; Kitaoka, M.; Nishimoto, M.; Ohtsubo, K. i.; Nakai, H. Discovery of β-1, 4-D-mannosyl-N-acetyl-D-glucosamine phosphorylase involved in the metabolism of N-glycans. *Journal of Biological Chemistry* **2013**, *288* (38), 27366-27374.

(9) Pettersen, E. F.; Goddard, T. D.; Huang, C. C.; Couch, G. S.; Greenblatt, D. M.; Meng, E. C.; Ferrin, T. E. UCSF Chimera—a visualization system for exploratory research and analysis. *Journal of computational chemistry* **2004**, *25* (13), 1605-1612.
